# Lack of Genomic Heterogeneity at High-Resolution aCGH between Primary Breast Cancers and Their Paired Lymph Node Metastases

**DOI:** 10.1371/journal.pone.0103177

**Published:** 2014-08-01

**Authors:** Marieke A. Vollebergh, Christiaan Klijn, Philip C. Schouten, Jelle Wesseling, Danielle Israeli, Bauke Ylstra, Lodewyk F.A. Wessels, Jos Jonkers, Sabine C. Linn

**Affiliations:** 1 Division of Molecular Pathology, Netherlands Cancer Institute–Antoni van Leeuwenhoek Hospital, Amsterdam, the Netherlands; 2 Department of Pathology, Netherlands Cancer Institute–Antoni van Leeuwenhoek Hospital, Amsterdam, the Netherlands; 3 Department of Bioinformatics and Statistics, Netherlands Cancer Institute–Antoni van Leeuwenhoek Hospital, Amsterdam, the Netherlands; 4 Division of Medical Oncology, Netherlands Cancer Institute–Antoni van Leeuwenhoek Hospital, Amsterdam, the Netherlands; 5 Department of Pathology, Vrije Universiteit University Medical Center, Amsterdam, the Netherlands; 6 Faculty of Electrical Engineering, Mathematics and Computer Science, Delft University of Technology, Delft, the Netherlands; University Medical Centre Utrecht, Netherlands

## Abstract

Lymph-node metastasis (LNM) predict high recurrence rates in breast cancer patients. Systemic treatment aims to eliminate (micro)metastatic cells. However decisions regarding systemic treatment depend largely on clinical and molecular characteristics of primary tumours. It remains, however, unclear to what extent metastases resemble the cognate primary breast tumours, especially on a genomic level, and as such will be eradicated by the systemic therapy chosen. In this study we used high-resolution aCGH to investigate DNA copy number differences between primary breast cancers and their paired LNMs. To date, no recurrent LNM-specific genomic aberrations have been identified using array comparative genomic hybridization (aCGH) analysis. In our study we employ a high-resolution platform and we stratify on different breast cancer subtypes, both aspects that might have underpowered previously performed studies.To test the possibility that genomic instability in triple-negative breast cancers (TNBCs) might cause increased random and potentially also recurrent copy number aberrations (CNAs) in their LNMs, we studied 10 primary TNBC–LNM pairs and 10 ER-positive (ER+) pairs and verified our findings adding additionally 5 TNBC-LNM and 22 ER+-LNM pairs. We found that all LNMs clustered nearest to their matched tumour except for two cases, of which one was due to the presence of two distinct histological components in one tumour. We found no significantly altered CNAs between tumour and their LNMs in the entire group or in the subgroups. Within the TNBC subgroup, no absolute increase in CNAs was found in the LNMs compared to their primary tumours, suggesting that increased genomic instability does not lead to more CNAs in LNMs. Our findings suggest a high clonal relationship between primary breast tumours and its LNMs, at least prior to treatment, and support the use of primary tumour characteristics to guide adjuvant systemic chemotherapy in breast cancer patients.

## Introduction

Breast cancer is the result of a multistep process of genetic alterations in which normal breast cells are transformed into malignant cells and finally into cells with metastatic capacity [1], [2]. Lymph node metastasis (LNM) formation is a crucial step in disease progression as their presence implicates a poor prognosis and occurs irrespective of tumour size, grade or hormone receptor status. It has been speculated that breast cancer cells need to acquire a specific trait before they are able to metastasize [3]. However, it remains unclear to what extent LNMs differ from their primary breast tumours and whether this trait is acquired through genomic change. It is important to assess the genomic difference between LNMs and primary tumour for two reasons: 1). Any recurrent genomic difference alteration found in LNMs might hint to a molecular mechanism enabling metastasis, possibly leading to new avenues of treatment. 2). Systemic treatment of cancer aims to eradicate metastatic cells specifically. However, adjuvant systemic treatment decisions are made on characteristics of the primary tumour. In order for these systemic treatments to be successful, LNMs should be similar to the primary tumour. One way to check for similarity is to test whether the genomic constitution of LNMs is identical to their primary tumours.

Our study focuses on gross DNA copy number changes as genomic alterations in breast cancer. A fair number of studies have investigated genomic differences between primary breast tumours and lymph node or distant metastases by visualizing chromosomal losses and amplifications using either comparative genomic hybridization (CGH) or array-based CGH (aCGH) [4]–[10]. These studies either identified different regions of significant difference [5], [8], [10] or found no significant differences [4], [6], . However, most of these studies used low-resolution platforms detecting only large differences in copy number aberrations (CNAs). Therefore, smaller or more subtle changes, such as focal aberrations, could have been missed by these studies [11]. Furthermore, previous studies did not differentiate between subtypes of breast cancer. It has become clear that breast cancer is a heterogeneous disease with subtypes influencing prognosis and genomic profile [12]–[14]. Differences present in one subtype might have been diluted by the lack of difference in another subtype. In our study we differentiate between two distinct breast cancer subtypes: (1) the luminal subtype expressing the oestrogen receptor (ER) and (2) the basal-like subtype lacking ER, progesterone receptor (PgR) and HER2 expression (also known as triple-negative breast cancers or TNBCs) [12], [13]. On a genomic level the differences between these subtypes mainly manifests in the absolute number of CNAs, i.e. the number of gains and losses present in these subtypes. This is a frequently used and relatively good measure of genomic instability, even if it does not take into account mutations, small indels or copy number neutral rearrangements. Basal-like, TNBCs have been shown to have the highest number of CNAs, i.e. the highest level of genomic instability [15]–[18].

We hypothesized that using a high-resolution aCGH platform, and stratifying on different subtypes of breast cancer, we would be able to detect specific copy number changes between primary tumours and their LNMs if they occur. Furthermore, we speculated that LNMs of TNBCs would have more copy number aberrations than their primary tumours when compared to luminal tumours due to highest level of genomic instability. To explore these hypotheses, we analysed 10 TNBCs and 10 ER-positive tumours as well as their 20 paired LNMs on a 720 K Nimblegen aCGH platform and did an additional analysis by adding five TNBCs and 22 ER-positive tumour – LNM pairs on a 135 K Nimblegen aCGH platform. We found that regardless of subtype, metastases closely resembled their primary tumours, without specific recurrent copy number differences being present, suggesting that the potential to form metastases might not require specific recurrent CNAs.

## Methods

### Ethics Statement

All patients had given written informed consent to be included in the multicentre clinical trial described below, which had been approved by the institutional review committees at each of the participating centres (for details see Rodenhuis et al. [19]). According to Dutch law, this implied consent for the analysis of residual tissue specimens that had been obtained for diagnostic purposes and anonymized publication of the results according to Dutch guidelines. (http://www.federa.org/code-goed-gebruik-van-lichaamsmateriaal-2011).

### Patients

Patients were part of a multicentre adjuvant trial performed in the Netherlands (1993–1999), in which breast cancer patients with at least four tumour-positive axillary lymph nodes but no distant metastases participated [19]. No previous cancers were allowed and as such patients were chemotherapy-naïve before surgery. All subtypes were determined by protein expression measured by immunohistochemistry (IHC). For this study we initially randomly selected a first set consisting of ten patients with ER-, PR- and HER2-negative tumours, i.e. the TNBC group and ten patients with ER-positive, HER2-negative tumours, i.e. the ER+ group. Although ER+ tumours are generally classified into luminal A and luminal B subtypes using gene expression microarrays [11], [12], there was no gene-expression data available; as such this group will be referred to as ER-positive (ER+). To verify our results, we added a second set of twenty-two patients to the ER+ group and five patients to the TNBC group. Selection of patients was based upon availability of formalin-fixed paraffin-embedded (FFPE) tissue containing more than 60% tumour cells in both the primary tumour and their paired LNMs. If more than one LNM was available the LNM with the highest tumour cell content was chosen.

### Histopathology

Hematoxylin & Eosin (H&E) slides were scored for tumour percentages by a breast cancer pathologist (JW). ER, PR, P53, and HER2 were determined by immunohistochemistry as described previously [19], [20].

### DNA extraction and array comparative genomic hybridization

Genomic DNA was extracted from FFPE primary tumours and their LNMs as previously published [21]. DNA from Kreatech (Megapool reference DNA female, EA-100F) was used as reference DNA. Tumour and reference DNA was labelled as previously described [22], except for the amount of input which was increased from 500 ng DNA previously, to 1 ug in concordance with the larger hybridization area of the 720 K Nimblegen arrays. Labelled DNA of the first set of patients was hybridized on a 720 K Nimblegen CGH array (human CGH 3×720 k whole-genome tiling v3.1 arrays, GEO platform GPL 14965), containing 719690 unique in situ synthesized 60-mer oligonucleotides. Due to default of the Nimblegen company, additional 720 K arrays were no longer available. Therefore DNA of the second set of patients were analysed using a 135 K Nimblegen array (human CGH HX12 135 K whole-genome tiling v3.1 arrays), containing 134937 unique synthesized 60-mer oligonucleotides. The input for these smaller arrays was decreased to 500 ng for tumour as well as reference DNA (Promega, G1521 Female DNA); Labelling protocol was according to our previous report [23]. Hybridization on both arrays was performed according to manufacturer's protocol (Roche Nimblegen, Madison, USA). Slides of the arrays were scanned using a G2505C microarray scanner (Agilent Technologies). Images were analysed using DNAcopy, part of Nimblescan version 2.5.26 feature extraction software (Roche Nimblegen, Madison, USA). Oligonucleotides were mapped according to the human genome build NCBI36. The data discussed in this manuscript have been deposited in NCBI's Gene Expression Omnibus and are accessible through GEO Series accession number GSE38888 and GSE56765.

### Bioinformatics analyses

The primary first set of patients was analysed separately and results were verified in an analysis including the additional patients assayed on the low-resolution platform. For this second analysis the data of the 720 K arrays was downscaled to 135 K by using only the probes that are present on the 135 K arrays (all 134937 probes). Background corrected, non-segmented data output by the NimbleScan software were analysed using the R statistical programming language. All analyses were performed in this language (R-scripts available on: https://github.com/SeeLittle/LNCGH) unless otherwise noted. The aCGH data reported in this manuscript have been deposited in the GEO database (accession numbers GSE38888 and GSE56765).

### Heatmap

We computed a smoothed profile for each sample using the comparative module in the *KC-smart* R package [24], as implemented in the Bioconductor toolbox [25]. We set the kernel size for smoothing at σ = 1 Mb. We calculated the correlation distance (1 – Pearson's correlation) between all smoothed tumour profiles and used hierarchical clustering (average linkage) to construct the heatmap.

### Comparative analysis of CNAs

Using the comparative module in the *KC-smart* package, designed to identify significantly differential recurrent CNAs between two groups of samples, we analysed all samples for significant copy number differences between the primary tumour and the matched LNM [25]. We also analysed the TNBC and ER+ groups separately. We used a kernel size of σ = 1 Mb, significance cut-off at p<0.05 and 1000 permutations.

### Pairwise analyses of primary tumour and lymph node

As any group-wise analysis might miss low frequency or specific pair-wise changes, we analysed each tumour-LNM pair separately for samples assayed on the high resolution platform. To allow for direct comparison of tumour and LNM samples we applied quantile normalization on the dataset. As the distributions of the data were not significantly different this makes the samples directly comparable by profile subtraction without losing information. After normalization we subtracted the tumour profile from the LNM profile, creating a so-called ‘delta-profile’. These delta profiles were then segmented by the *DNAcopy* package as implemented in the *Bioconductor* toolbox (version 2.8). Segmentation parameters were standard, except that we used the option to undo a breakpoint call if the two segments adjacent to the breakpoint did not have mean probe values that were at least two standard deviations apart. When the breakpoint was undone the adjacent segments were merged. We tested whether the number of segments of the delta profiles per tumour – LNM pair was significant using the Wilcoxon rank sum test. To find recurrent pair-wise differential CNAs we searched for overlapping segments among all delta profiles. As a measure of genomic instability-induced change between the primary tumour and the LNM we counted the number of segments in the delta profiles that showed an absolute difference between the primary tumour and the LNM above 0.2 on a log2 scale. For these segments we also determined the copy number state in the primary tumour and the LNM sample. We called the regions in the individual samples ‘gain’ if the segmentation mean was above 0.2 and ‘loss’ if it was below -0.2. We did not perform these analyses on the whole group, since i) a lower resolution platform is less suitable for the identification of small or subtle changes, such as focal aberrations, the aim of this analysis and ii) quantile normalization requires a similar distribution of measurements. The low-resolution profiles demonstrated differential noise (not hybridized on the same array, visually more noisy) in the measurements, which would be amplified in the quantile normalization process rendering them unusable for this approach.

## Results

**Figure 1:**
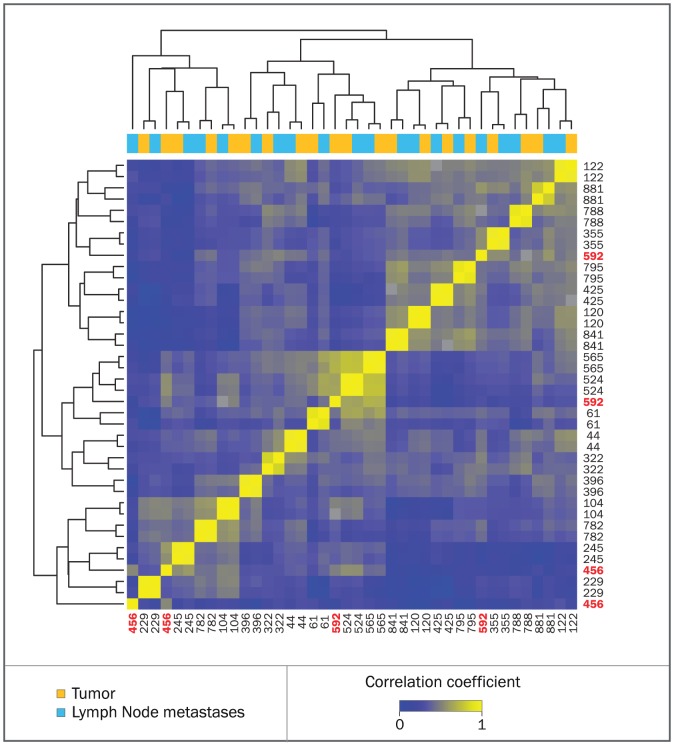
Heatmap of a hierarchical clustering based on copy number profile. Samples are hierarchically clustered with correlation as distance on their smoothed copy number profiles. The intensity of correlation is shown from blue to yellow. The colour bar above the heatmaps denotes the origin of the sample, either a primary tumour or a lymph node metastasis. The numbers on the y-axis are the patient numbers. Patient numbers in red show primary tumours and lymph node samples that do not cluster together.

We hybridized DNA from 20 tumours and paired LNM on the high-resolution 720 K Nimblegen platform, resulting in 40 aCGH profiles (File S1). Patient characteristics are shown in Table S1 in File S4. Hierarchical clustering of the 40 genomic profiles revealed that all LNMs clustered nearest to their matched tumour, except for two (Figure 1).

**Figure 2:**
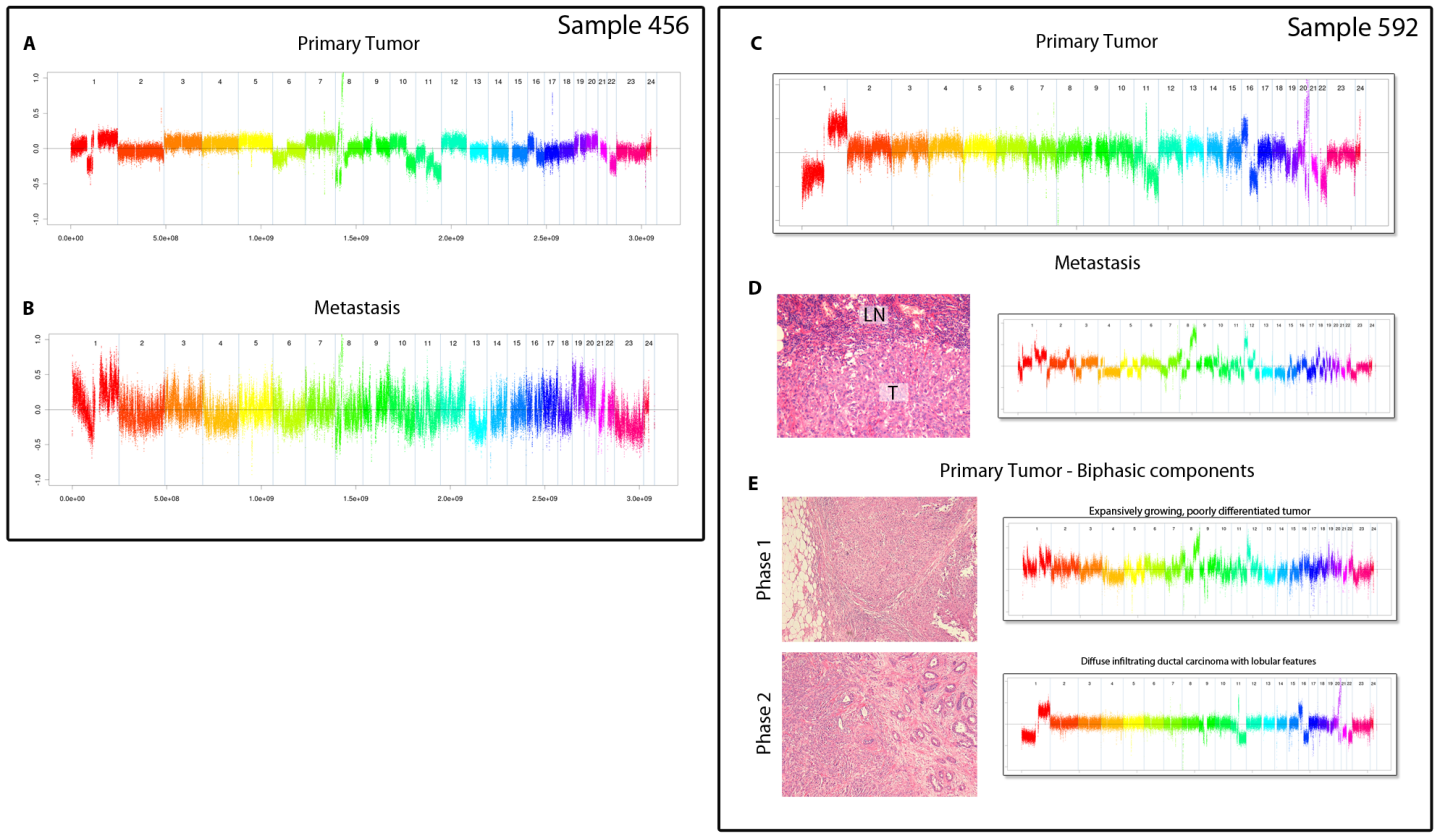
Investigation into discordant samples. Two samples that did not cluster together based on correlation were investigated for their discordance. A and B) Primary tumour and metastasis of patient 456 showed visually similar profiles, but the metastasis was a highly noisy hybridization. C and D) Primary tumour and metastasis profiles for patient 592 showed two highly different profiles. E) Two different histological phases were detected in the primary tumour, of which DNA was isolated separately and hybridized again.

The aCGH profiles of these two non-concordant pairs were evaluated to determine why the LNMs did not resemble their primary tumour. For patient 456 we found that although visually the profiles were similar, the hybridization was of such poor quality that the correlation distance was quite high (Figure 2, A and B). In contrast, the LNM of patient 592 had no quality issues and appeared to have originated from a different tumour (Figure 2, C and D). By reviewing an H&E staining of the tumour, it was determined to consist of at least two components: an expansively growing, poorly differentiated part and a diffusely infiltrating part exhibiting lobular features (Figure 2E). DNA from both components was isolated and hybridized separately (Figure 2E). Using the same clustering method, including the profiles of these two separately isolated components, we found that the 592 expansively growing component clustered most closely with the 592 LNM This indicates that this component was most likely the origin of the metastasis (Figure S1).

We excluded both non-resembling pairs (patient 456 and 592) from further analyses, since they would introduce artificial differences (either due to noise or due to a primary tumour composed of two, molecularly distinct components).

**Figure 3:**
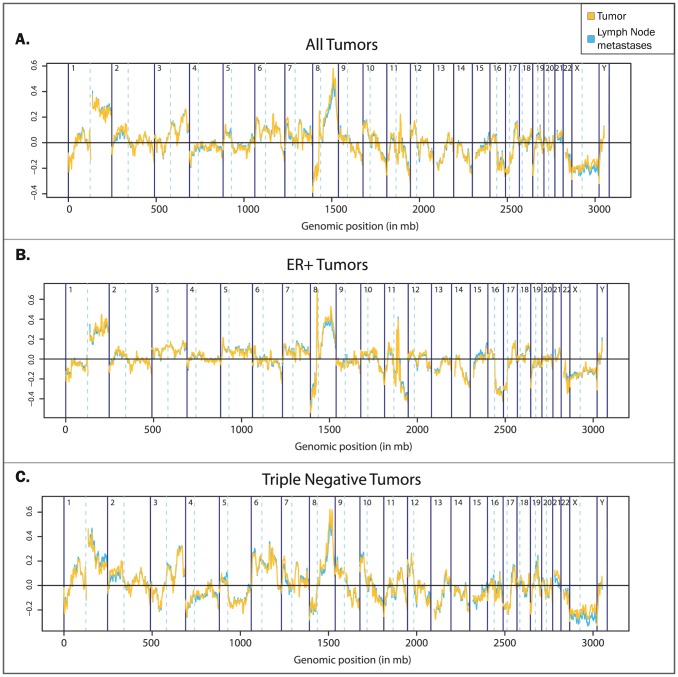
Group-wise comparison of primary tumour copy number profiles with lymph node metastasis copy number profiles. Overall profiles were generated using the comparative module of the *KC-smart* package on either (A) the whole group, (B) just the ER+ tumours and (C) the triple negative tumours.

We searched for any recurrent CNAs differing between the primary tumour and its LNM using the remaining samples. The smoothed *KC-smart* profiles were highly similar and no significant differences were detected when all samples were analysed simultaneously (Figure 3A). However, subtype-specific differences might have been obscured due to pooling of data instead of taking into account the specific breast cancer subtypes. We therefore analysed the pairs within the ER+ and TNBC subtypes separately, but again found no significantly different regions between the pairs within either subtype (Figure 3B and 3C).

**Figure 4:**
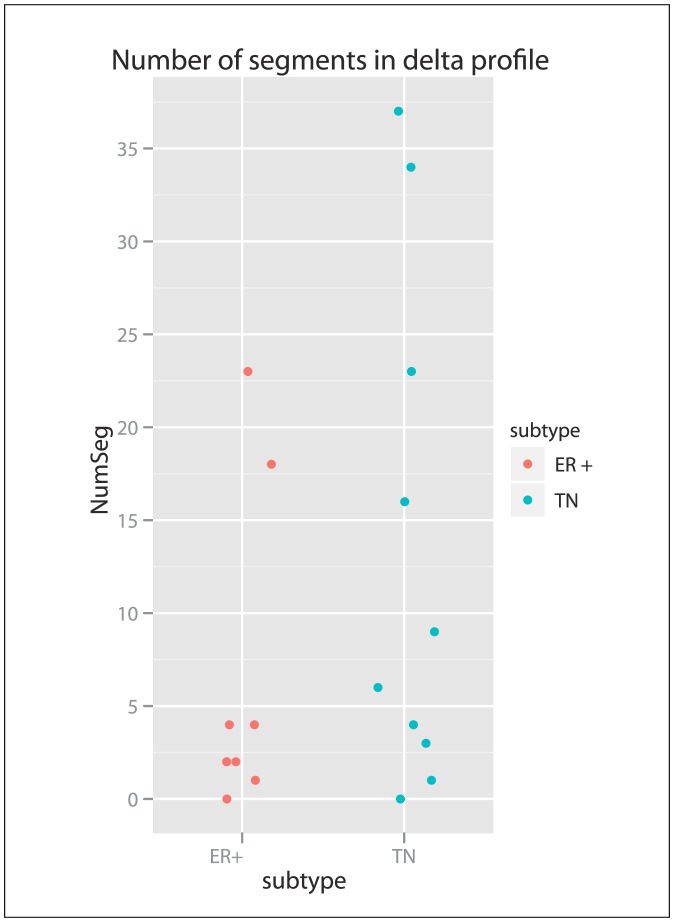
Quantification of copy number differences between the tumour and lymph node metastasis. For each sample pair (Tumour-Lymph Node) we constructed a delta profile, which contains only the copy number differences between the two. For each delta profile we counted the unique segments that showed a difference above .2 log2 or below -.2 log2 and we subsequently counted the segments that contained 10 or more probes only. We split the tumours into the TNBC and ER+ subgroups.

We next tested our hypothesis whether LNMs of TNBCs showed more genomic differences than their primary tumours when compared to ER+ tumours. To do this we constructed ‘delta-profiles’, normalized pair-wise subtractions of the tumour profile from its paired LNM profile. By counting the number of segments of the delta profiles per tumour – LNM pair (delta-segments) we found no significant difference between the TNBC and ER+ subtype (Figure 4).

We also checked whether we could identify overlapping delta segments between patients, indicating a recurrent change between tumour and LNM. Although we found a number of overlapping delta segments between tumour and LNM (Table S2 in File S4), none of these showed a consistent direction of change between the overlapping CNAs, indicative of random change instead of a driving alteration (File S2).

To ascertain whether our findings would be sustained in a larger dataset, 22 patients were added to the ER+ group (total n = 32) and five patients to the TNBC group (total n = 15. Patient characteristics of the whole dataset are shown in Table S3 in File S4 and aCGH profiles in File S3). No substantial differences were found analysing this larger dataset; LNMs clustered nearest to their matched tumour on hierarchical clustering (Figure S2A), no significant differences were seen between the smoothed KC-smart profiles of LNMs and the primary tumours within the whole group (Figure S2B), nor within the different subtypes (Figure S2C and S2D).

## Discussion

Eradication of disseminated tumour cells is the primary goal of adjuvant systemic treatment in breast cancers patients. Decisions regarding systemic treatment in all stages of breast cancer (early to metastatic) are largely based on histopathological and molecular characteristics of the primary tumour rather than the disseminated tumour cells. In this study we investigated the genomic differences between primary breast cancers and their paired LNMs by using a high-resolution aCGH platform. We hypothesized that metastasis-specific recurrent DNA alterations have not been identified in the past due to the use of low-resolution BAC-based platforms and the lack of stratification on breast cancer subtypes (specifically, TNBC). Furthermore, we speculated that the genomic instability of TNBCs might result in an increase in CNAs in LNMs, simply due to random occurrence in this subgroup. Therefore, we studied a total of 15 primary breast tumour–LNM pairs of the TNBC subtype and 32 pairs of the ER+ tumours. We found that 1) most LNMs closely resembled their primary tumour with only small focal, non-recurrent CNA differences that were not statistically significant; 2) lack of resemblance was explained by poor quality hybridization or the presence of two histological components within one tumour; 3) no significantly regional differences occurred between primary tumours and LNMs overall or within the specific subtypes; and 4) the number of CNAs within the LNM compared to the primary tumour did not differ between TNBCs and ER+ tumours. These findings were confirmed in a larger dataset on a platform with a slightly lower resolution. Although TNBC tumours show more CNAs in total and are known to be more genomically unstable, the absolute amount of CNAs in the LNMs and their matched primary tumour are equal. The same holds for the ER+ tumours. Although one might expect additional random damage in a metastatic cell compared to its primary tumour simply due to high genomic instability, our results do not show this.

This is the first study in which a high-resolution aCGH platform was used to detect differences between primary breast tumours and paired LNMs. Furthermore, this is the first study taking into account breast cancer subtypes. Although our study did not include all subtypes, we speculated that by stratifying on the two most extreme subtypes with regard to number of CNAs [18], we would be most likely to find potential differences between primary tumours and their LNMs. In accordance with most previous studies [4], [7],[9], we observed in most cases strong resemblance between aCGH profiles of primary breast tumours and their LNMs. This suggests that metastasis formation does not require selection of a vastly different cell population. Comparable results were found when primary breast cancers and their metastases (both lymph node and distant) were compared using gene expression arrays [12], [26] and immunohistochemistry [27]. In our study only 2% (1/47) of the LNM truly did not resemble the cognate primary tumour, suggesting a strong clonal relationship between primary breast cancers and their LNMs. However, this one case does show that rarely a primary tumour can have very disparate populations, of which only one might lead to metastasis.

Recently, two studies have investigated the clonality within breast cancer by profiling genomes of cells sorted on ploidy of different subsections within the same tumour [28] and by quantifying copy number using next generation sequencing of single breast cancer cells [29]. It was shown that half of the breast tumours consisted of a homogenous clonal population, while the other half had around three clonal subpopulations. Even within these subpopulation the majority of chromosomal breakpoints were shared and only small differences based on focal amplifications or deletions were seen [28], [29]. This corresponds to our findings of focal differences between primary breast tumours and their LNMs found in 16 of the 20 pairs on high-resolution arrays (720 K) (Table S2 in File S4). Remarkably, we observed that even genomically unstable tumours such as TNBCs show a high similarity between primary tumours and their LNMs. Of course, copy number neutral changes, such as point mutations or balanced translocations and inversions, were not explored and primary tumours might therefore still differ more from their LNMs for those aberrations.

From previous studies it is known that TNBCs resemble *BRCA1*-mutated breast cancers closely with regard to their genomic instability [15]–[18]. BRCA1 is involved in DNA-repair of double-strand breaks (DSBs) by homologous recombination (HR). Failure of HR will result in error-prone repair of DSBs, resulting in gross chromosomal rearrangements and consequently genomic instability. These TNBCs resemble breast cancers of *BRCA1*-mutation carriers not only with respect to their level of genomic instability but also in terms of their gene expression patterns and their lack of expression of hormone receptors [30]. Furthermore, a subgroup of TNBCs also shares the characteristic genomic pattern of specific CNAs associated with *BRCA1*-mutated breast cancer [31], [32]. This suggests that this subgroup of sporadic TNBCs with a BRCA1-like aCGH profile might share the HR-deficient phenotype of *BRCA1*-mutated breast cancers. This hypothesis was strengthened by the fact that some of these BRCA1-like tumours showed *BRCA1*-promoter methylation [31], [32]. One could envision that in the absence of HR, repair of DSBs depends on error-prone DNA repair pathways resulting in random CNAs and genomic instability. Nevertheless, we found that even within TNBCs no increase of CNAs was seen in their LNMs, suggesting that even in genomically unstable tumours with large numbers of CNAs, the CNAs present are not randomly introduced and remain present in the population. This concept is supported by the CGH findings in mouse models of BRCA1-associated breast cancer, showing a highly similar pattern of CNAs in different BRCA1-deficient mouse mammary tumours, which was highly reproducible over different studies [33], [34].

In our study we found that biphasic tumours composed of two distinct molecular subtypes can cause discordance between primary tumours and lymph nodes. This finding is very interesting with regard to systemic treatment response, as it could explain the mixed responses (mostly cases where the primary tumour responds to treatment, but the LNM does not) that are sometimes observed within breast cancer patients upon neoadjuvant systemic treatment. Our results suggest that it may be useful to histologically evaluate the primary tumour and (if possible) the LNMs for the presence of multiple components in order to select the optimal systemic treatment.

Aside from this isolated, two-component case, there was little difference between primary tumours and their LNM. This apparent lack of difference suggests that intrinsic mechanisms of therapy response/resistance may be similar for both tumour and lymph node metastases, at least with regard to non-targeted therapies (i.e. most chemotherapeutics). Hence, neoadjuvant systemic therapy that leads to pathologic complete remission of the primary tumour might also be most effective for eradication of residual (disseminated) tumour cells in the adjuvant setting. The high level of similarity of genomic profiles further implicates that biomarkers based on genomic aberrations can also be tested on DNA from the LNMs instead of the primary tumour, which can be helpful in case insufficient primary tumour tissue is available.

In summary we found a high level of similarity between primary breast tumours and their LNMs, suggesting a strong clonal relationship between both tumour cell compartments. This finding argues that, at least at the DNA copy number level, there is no early evolutionary split between primary tumour cells and (locally) metastatic cells. Our findings argue against the notion that primary tumour cells need to accumulate many additional copy number changes to acquire local metastatic capacity and generally support the use of primary tumour characteristics for guiding the choice of adjuvant systemic therapy. However, our data also show that the existence of biphasic tumours composed of two distinct molecular subtypes may trigger differential responses to systemic therapy, which justifies molecular and histopathological characterization of both primary tumours and their LNMs.

## Supporting Information

Figure S1 Heatmap of a hierarchical clustering based on copy number profile. Samples are hierarchically clustered with correlation as distance on their smoothed copy number profiles. The intensity of correlation is shown from blue to yellow. The colored bar above the heatmaps denotes the origin of the sample, either a primary tumour or a lymph node metastasis. The numbers on the y-axis are the patient numbers. Patient numbers in red show primary tumours and lymph node samples that do not cluster together. Patient numbers in green show samples that were reassessed and visualized in Figure 2.
(EPS)

Figure S2 Follow-up analyses. A. Hierarchical clustering of 135 K aCGH profiles. Samples are hierarchically clustered with correlation as distance on their smoothed copy number profiles. The intensity of correlation is shown from blue to yellow. The colored bar above the heatmaps denotes the origin of the sample, either a primary tumour or a lymph node metastasis. The numbers on the y-axis are the patient numbers. Patient numbers in red show primary tumours and lymph node samples that do not cluster together. (B-D) Group-wise comparison of primary tumour copy number profiles with lymph node metastasis copy number profiles. Overall profiles were generated using the comparative module of the *KC-smart* package on either (B) the whole group, just the triple negative tumours (C) and just the ER+ tumours (D).
(PDF)

File S1 Visualizations of the 720 K copy number profile of all primary tumour – lymph node metastasis pairs that were included in all analyses. Each page shows the profiles for a single patient.
(PDF)

File S2 Visualizations of the delta segments that overlapped and exceeded an absolute log2 value of 0.1. Each page shows all delta segments found on a single chromosome. The upper row shows the raw data for a segment in the tumour sample. The middle row shows the raw data for a segment in the lymph node sample. The lower panel shows all delta segments and the values of the probes in the delta profile. Each patient is coloured differently.
(PDF)

File S3 Visualizations of the 135 K copy number profile of all primary tumour – lymph node metastasis pairs that were included in follow-up analyses. Each page shows the profiles for a single patient.
(PDF)

File S4 Tables S1-S3. **Table S1**, Patient characteristics of patients with 720 K aCGH data available. **Table S2**, Overview of all overlapping delta segments. **Table S3**, Patient characteristics of all patients.
(DOCX)
