# Supplementary material for: Lack of Genomic Heterogeneity at High-Resolution aCGH between Primary Breast Cancers and Their Paired Lymph Node Metastases
Source: PLoS One. 2014 Aug 1;9(8):e103177. doi: 10.1371/journal.pone.0103177 (PMC4118860; doi:10.1371/journal.pone.0103177)
Supplement: Figure S2 — Follow-up analyses. A. Hierarchical clustering of 135 K aCGH profiles. Samples are hierarchically clustered with correlation as distance on their smoothed copy number profiles. The intensity of correlation is shown from blue to yellow. The colored bar above the heatmaps denotes the origin of the sample, either a primary tumour or a lymph node metastasis. The numbers on the y-axis are the patient numbers. Patient numbers in red show primary tumours and lymph node samples that do not cluster together. (B-D) Group-wise comparison of primary tumour copy number profiles with lymph node metastasis copy number profiles. Overall profiles were generated using the comparative module of the KC-smart package on either (B) the whole group, just the triple negative tumours (C) and just the ER+ tumours (D). (PDF) [file pone.0103177.s002.pdf]

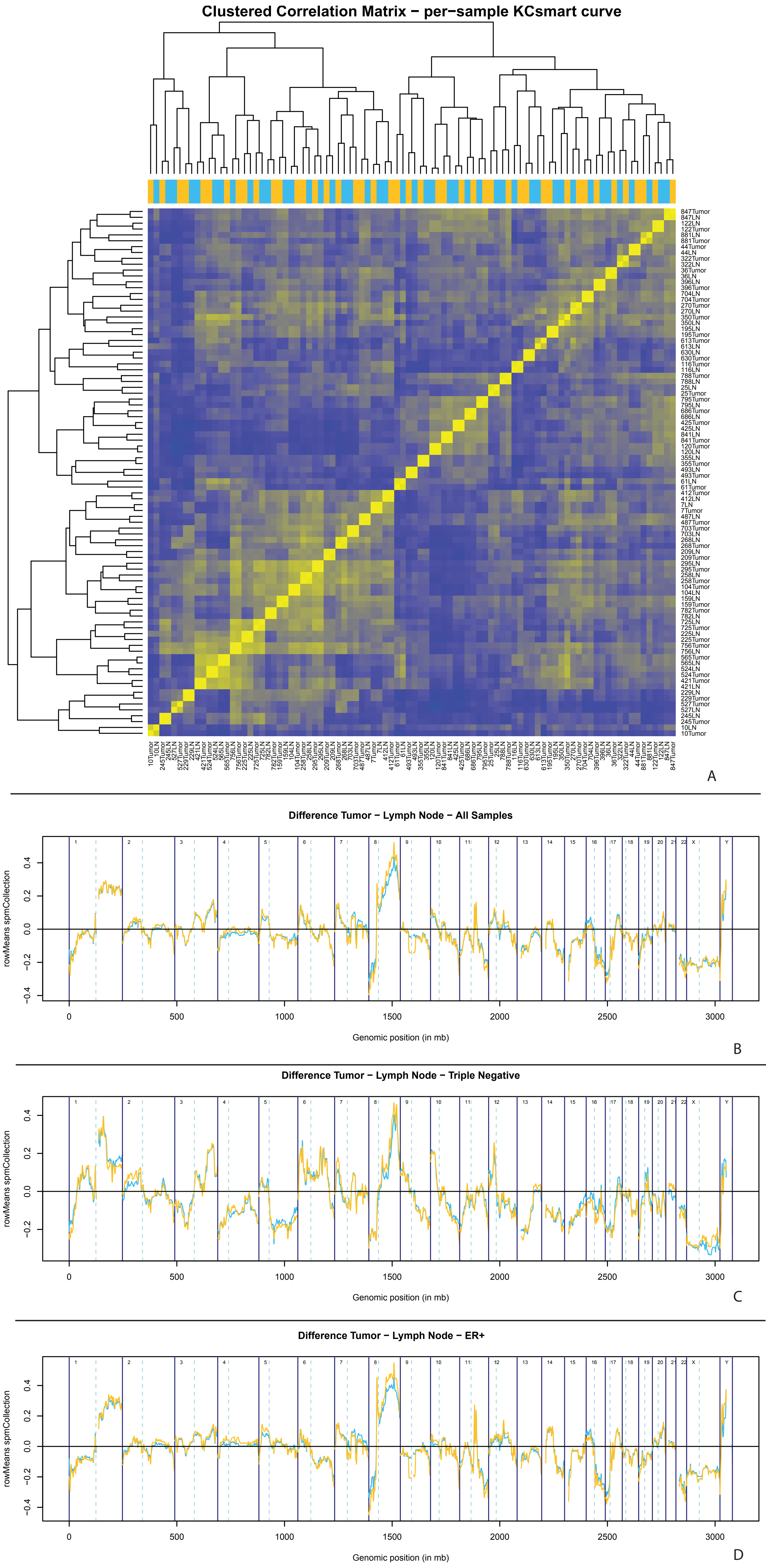

Clustered Correlation Matrix − per−sample KCsmart curve
A
Difference Tumor − Lymph Node − All Samples
rowMeans spmCollection
Genomic position (in mb)
B
Difference Tumor − Lymph Node − Triple Negative
rowMeans spmCollection
Genomic position (in mb)
C
Difference Tumor − Lymph Node − ER+
rowMeans spmCollection
Genomic position (in mb)
D
