# Supplementary figures and images for: Lack of Genomic Heterogeneity at High-Resolution aCGH between Primary Breast Cancers and Their Paired Lymph Node Metastases
Source: PLoS One. 2014 Aug 1;9(8):e103177. doi: 10.1371/journal.pone.0103177 (PMC4118860; doi:10.1371/journal.pone.0103177)

## Supplementary File 1

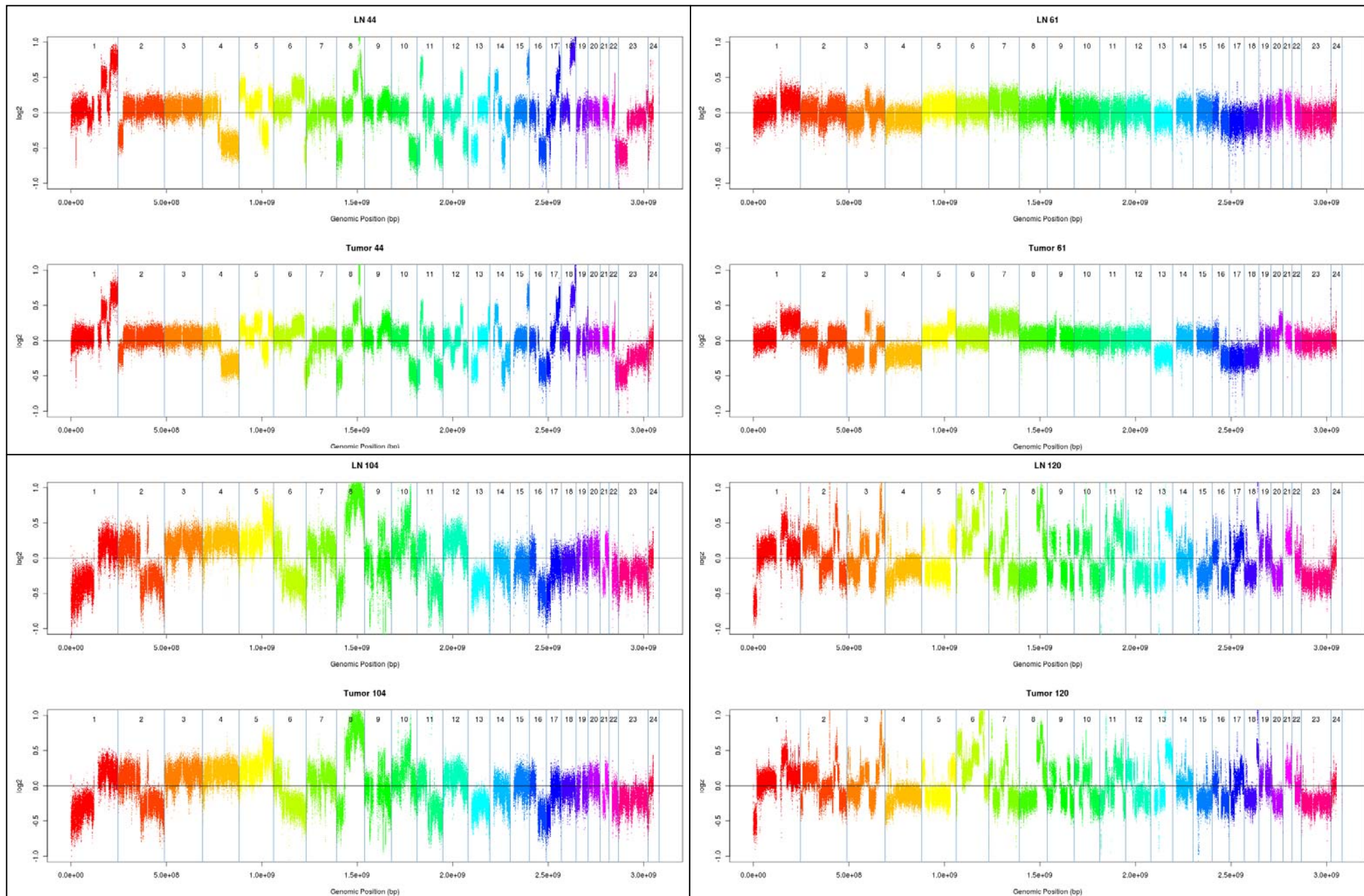

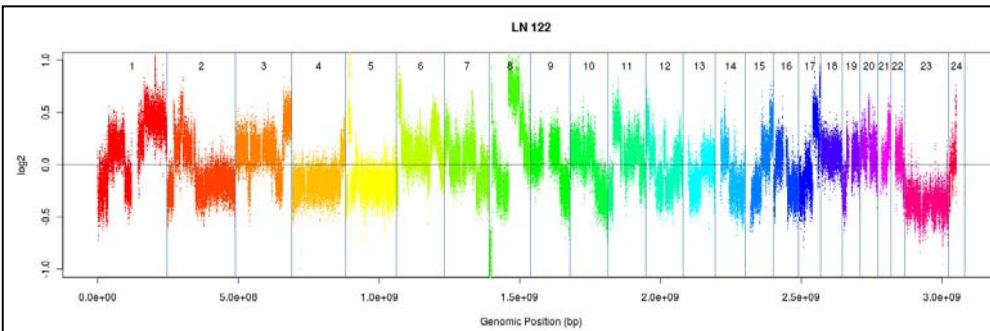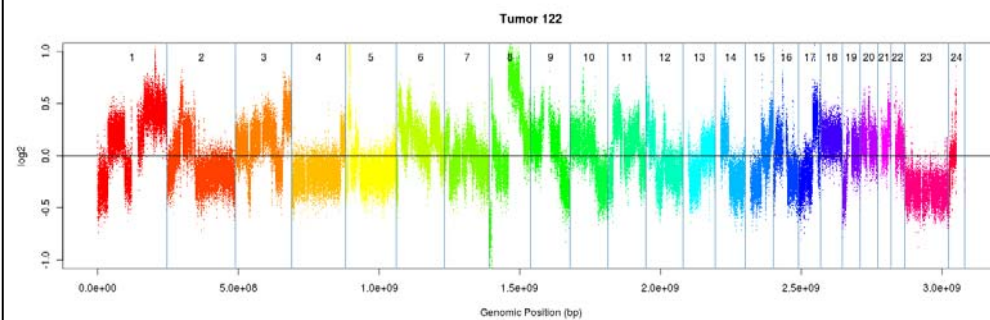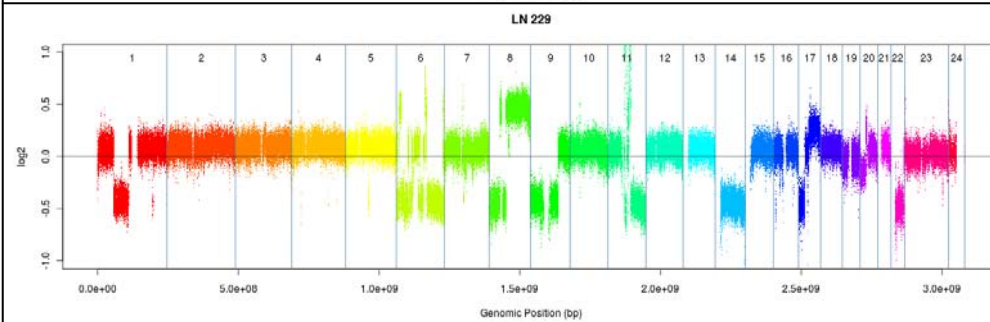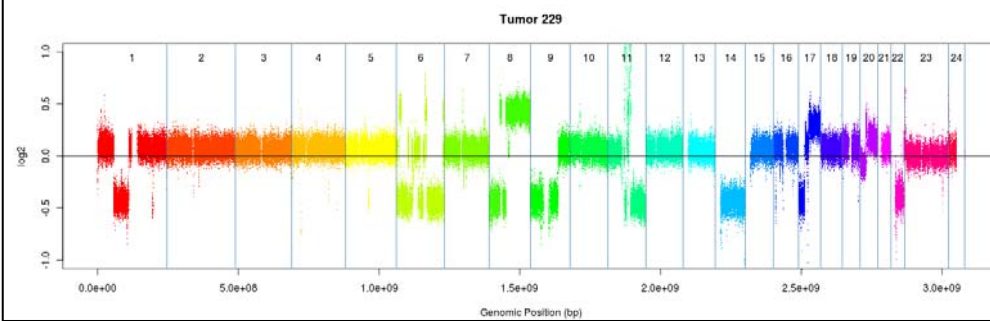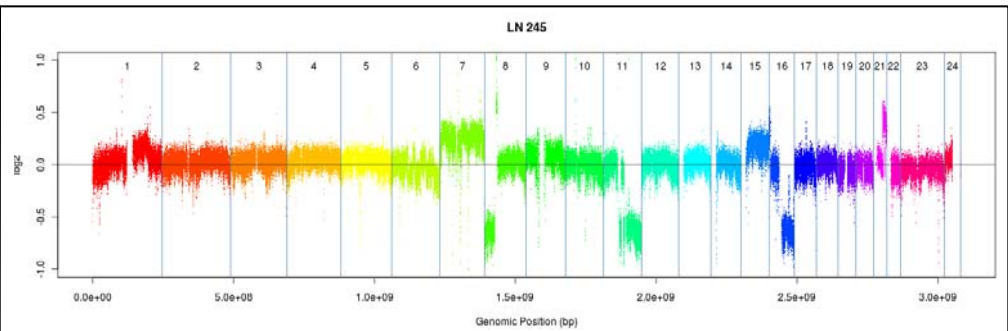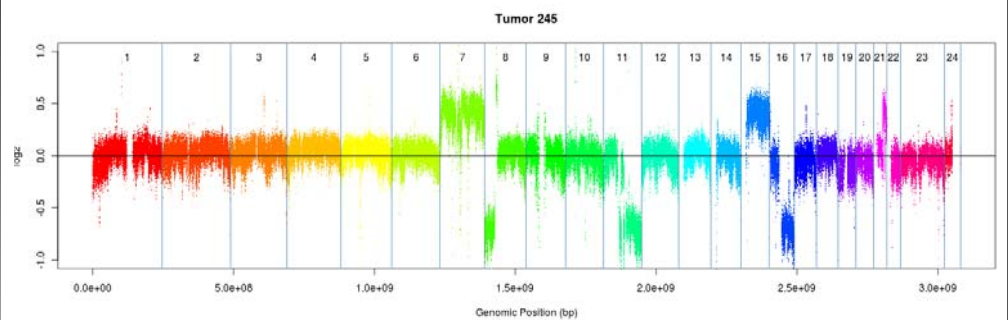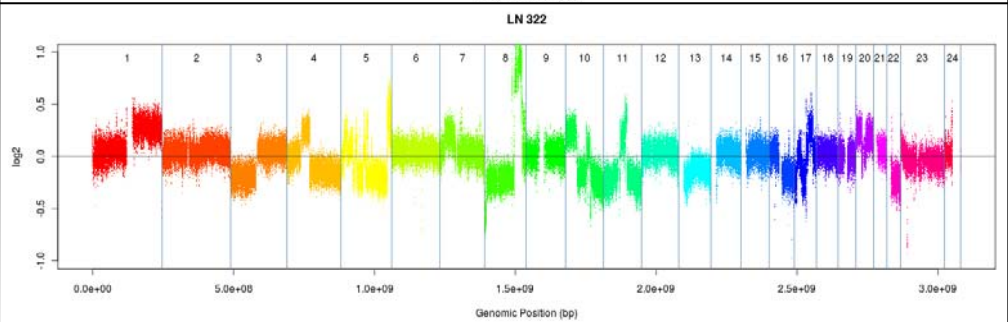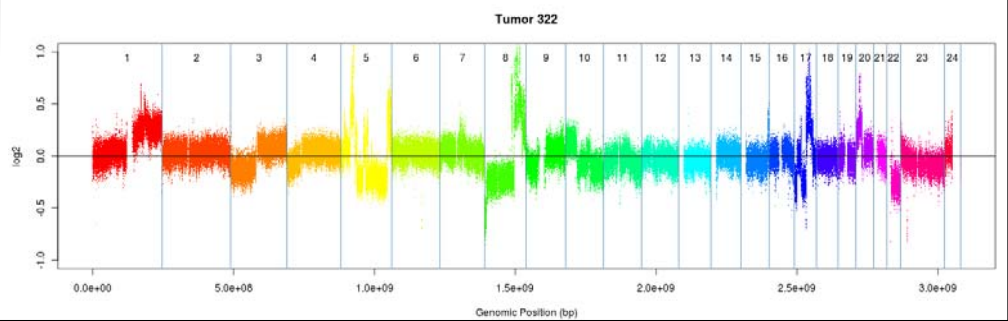

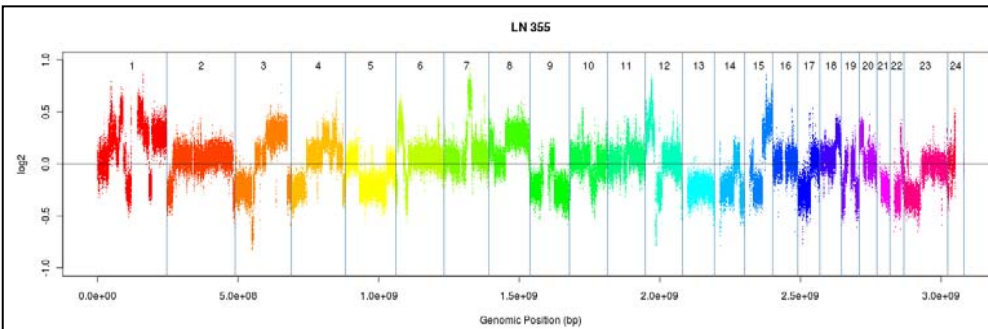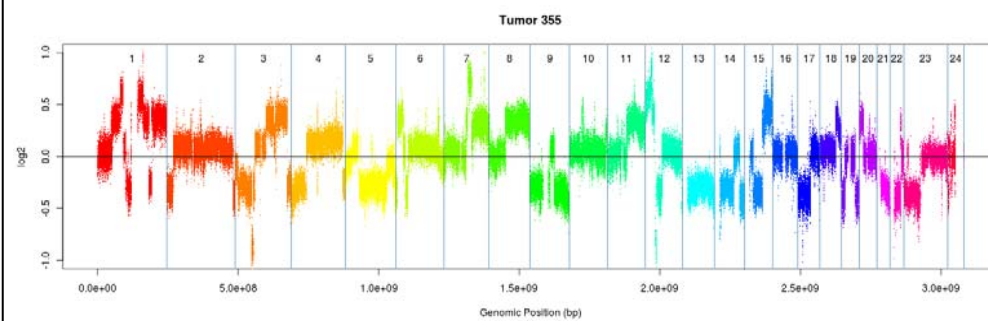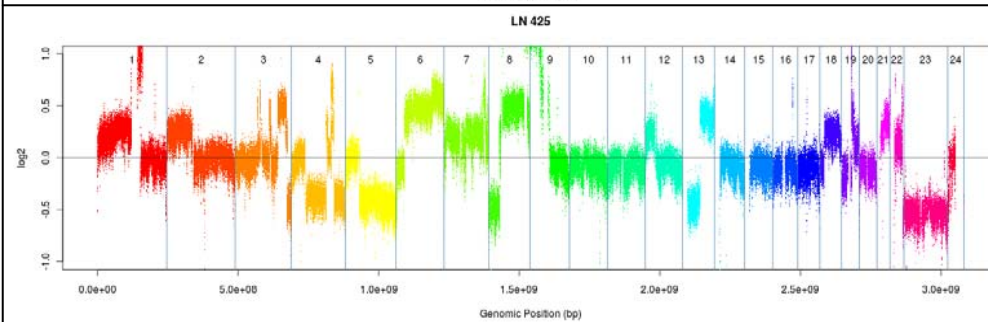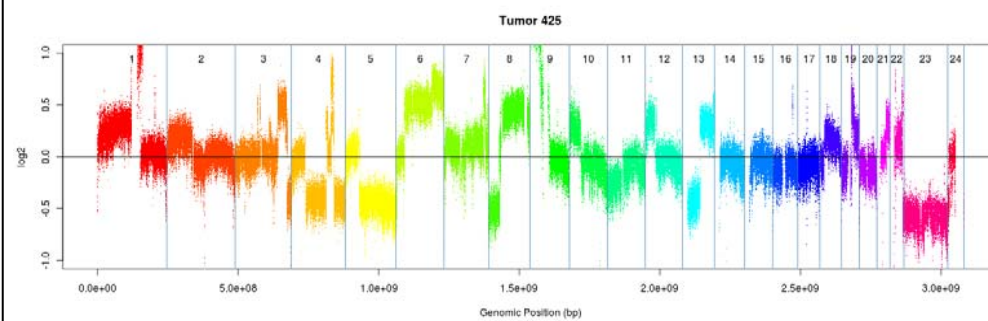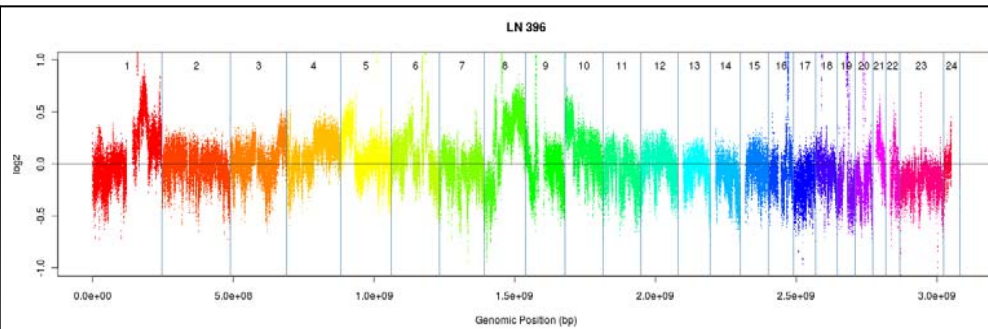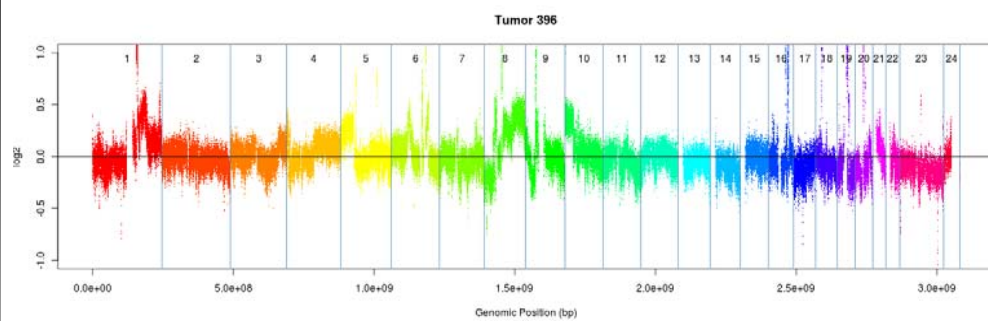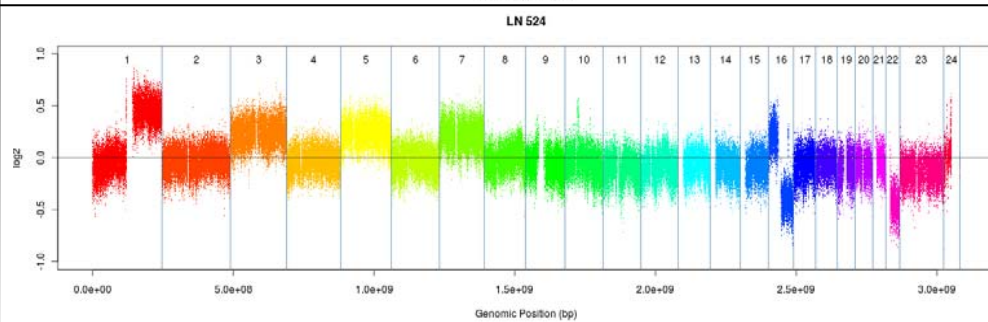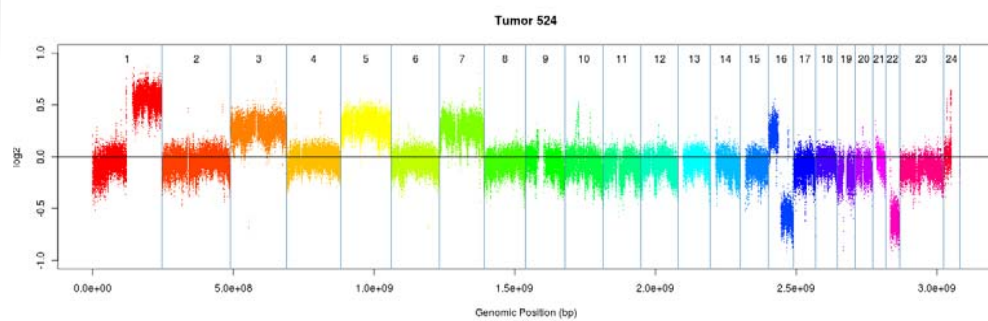



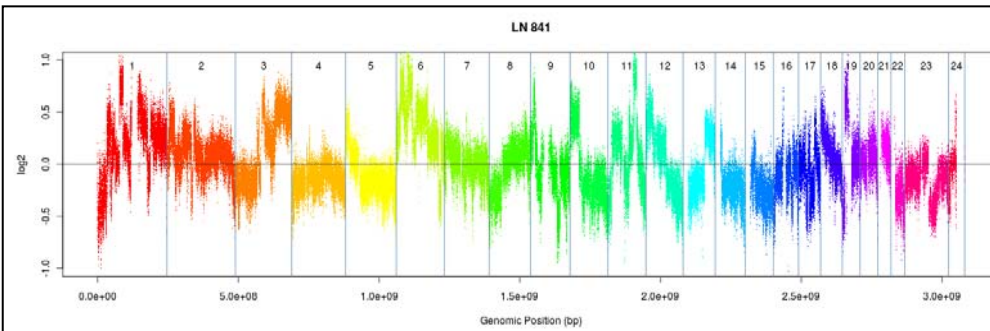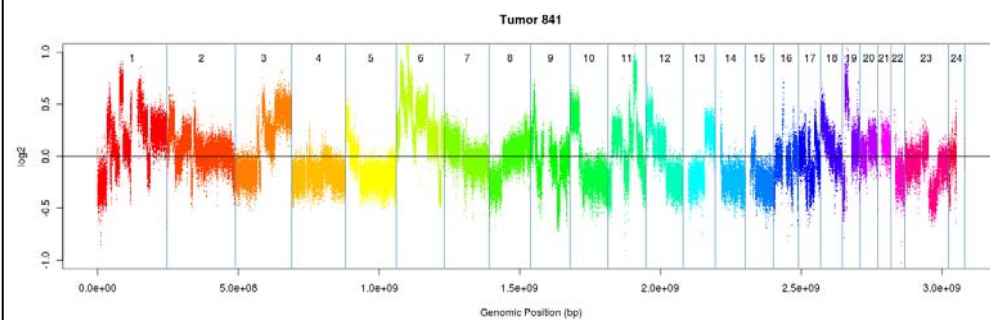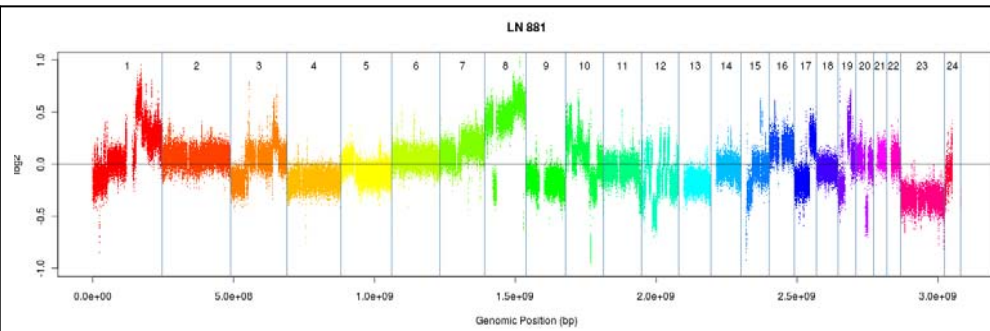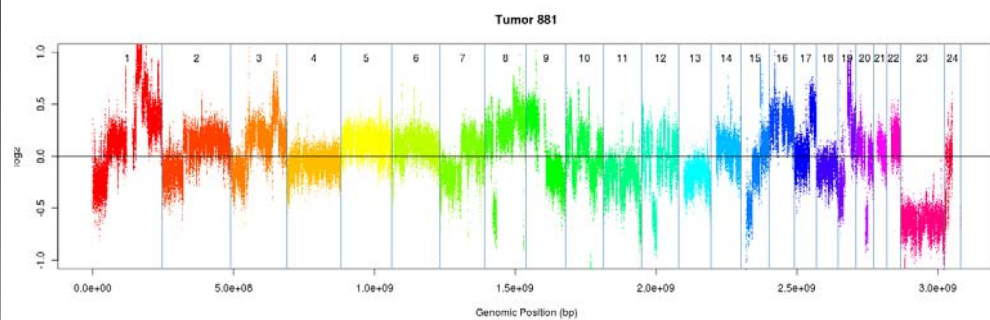

Supplement: File S1 — Visualizations of the 720 K copy number profile of all primary tumour – lymph node metastasis pairs that were included in all analyses. Each page shows the profiles for a single patient. (PDF) [file pone.0103177.s003.pdf]
