# Supplementary material for: Lack of Genomic Heterogeneity at High-Resolution aCGH between Primary Breast Cancers and Their Paired Lymph Node Metastases
Source: PLoS One. 2014 Aug 1;9(8):e103177. doi: 10.1371/journal.pone.0103177 (PMC4118860; doi:10.1371/journal.pone.0103177)
Supplement: File S2 — Visualizations of the delta segments that overlapped and exceeded an absolute log2 value of 0.1. Each page shows all delta segments found on a single chromosome. The upper row shows the raw data for a segment in the tumour sample. The middle row shows the raw data for a segment in the lymph node sample. The lower panel shows all delta segments and the values of the probes in the delta profile. Each patient is coloured differently. (PDF) [file pone.0103177.s004.pdf]

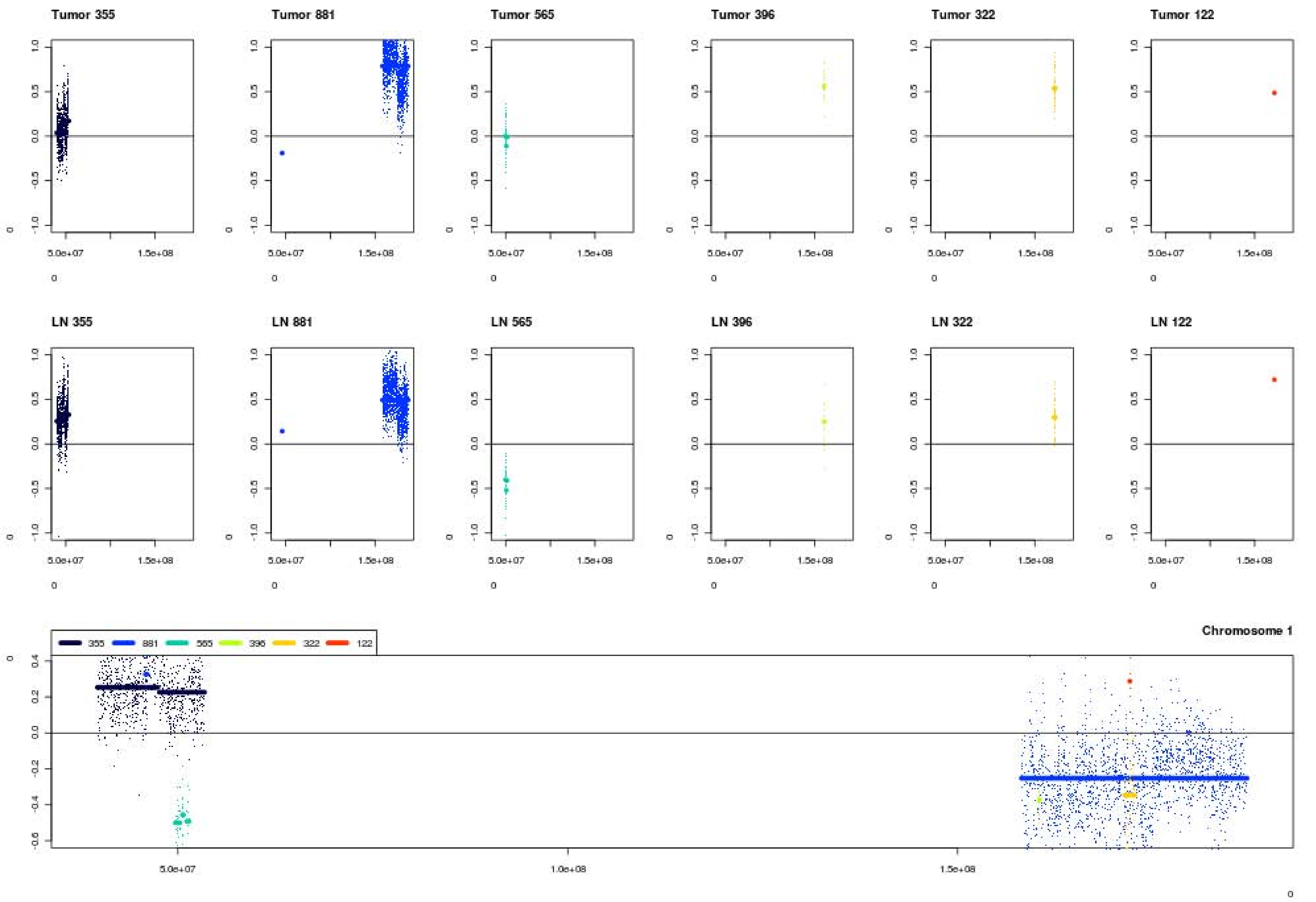
Tumor 355
Tumor 881
Tumor 565
Tumor 396
Tumor 322
Tumor 122
LN 355
LN 881
LN 565
LN 396
LN 322
LN 122
-1.0 -0.5 0.0 0.5 1.0
5.0e+07 1.5e+08
0
Chromosome 1
355 881 565 396 322 122
-0.6 -0.4 -0.2 0.0 0.2 0.4
5.0e+07 1.0e+08 1.5e+08

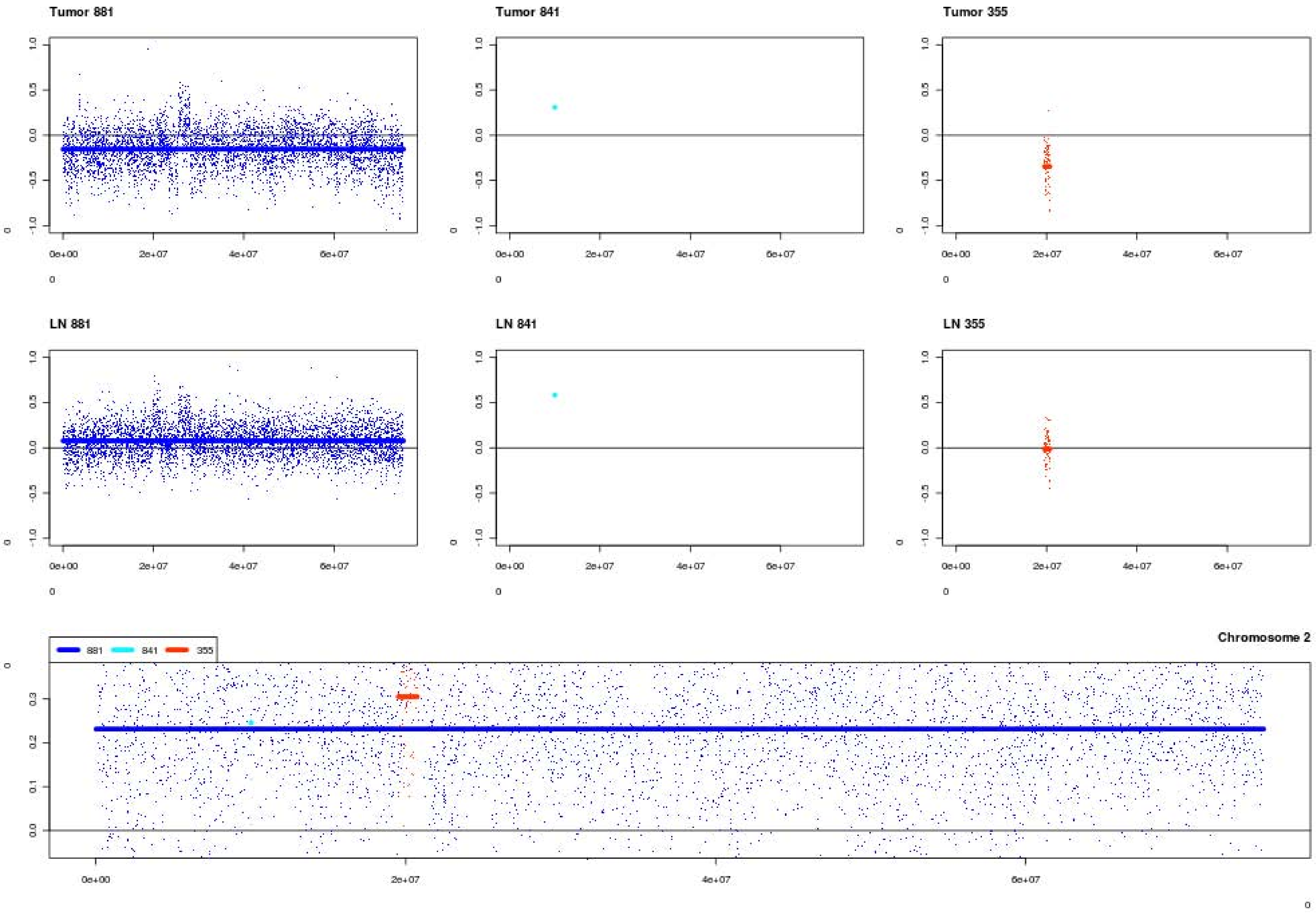

Tumor 881
Tumor 841
Tumor 355
LN 881
LN 841
LN 355
881
841
355
Chromosome 2

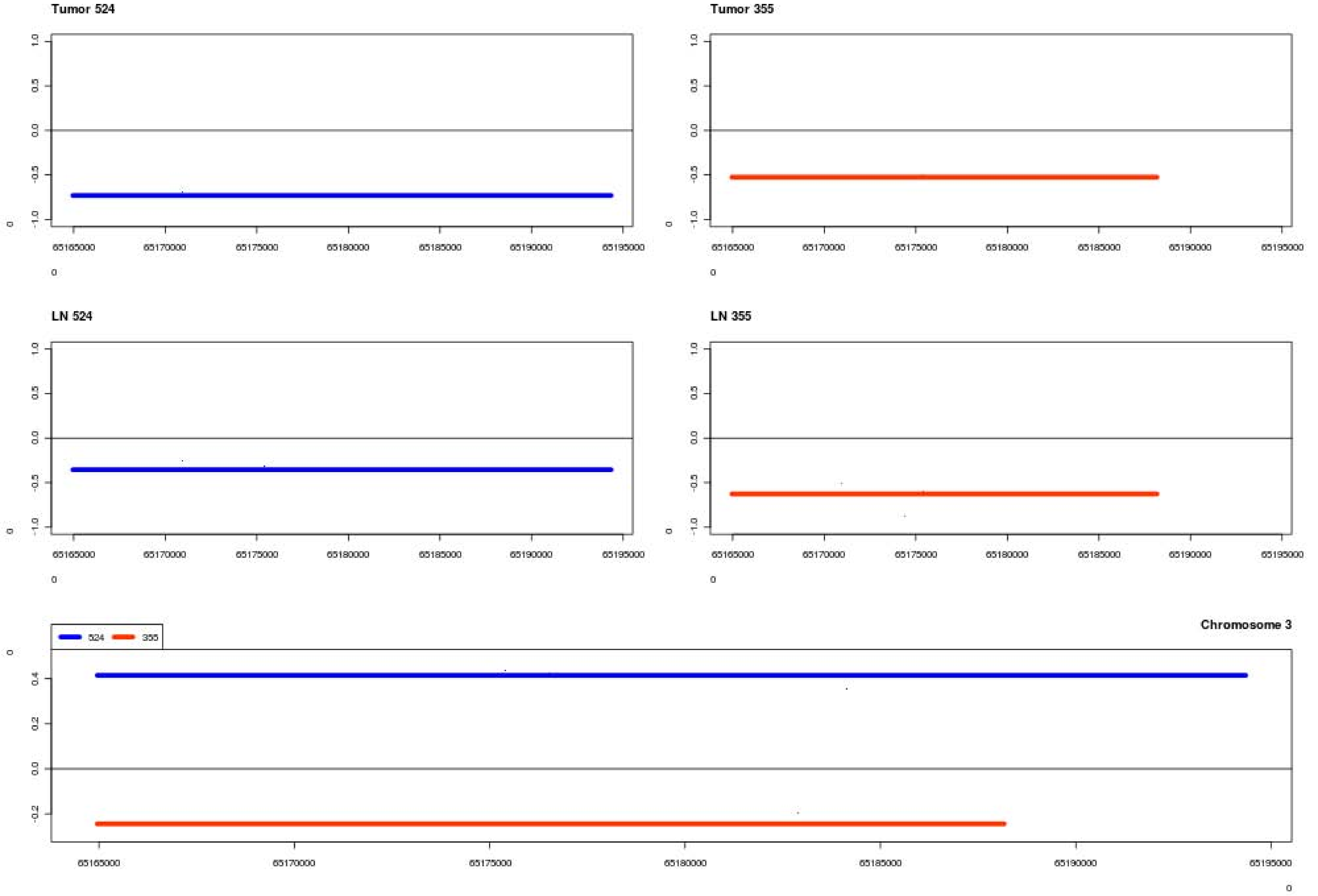
Tumor 524
Tumor 355
LN 524
LN 355
524
355
Chromosome 3
1.0
0.5
0.0
-0.5
-1.0
0.4
0.2
-0.2
65165000
65170000
65175000
65180000
65185000
65190000
65195000

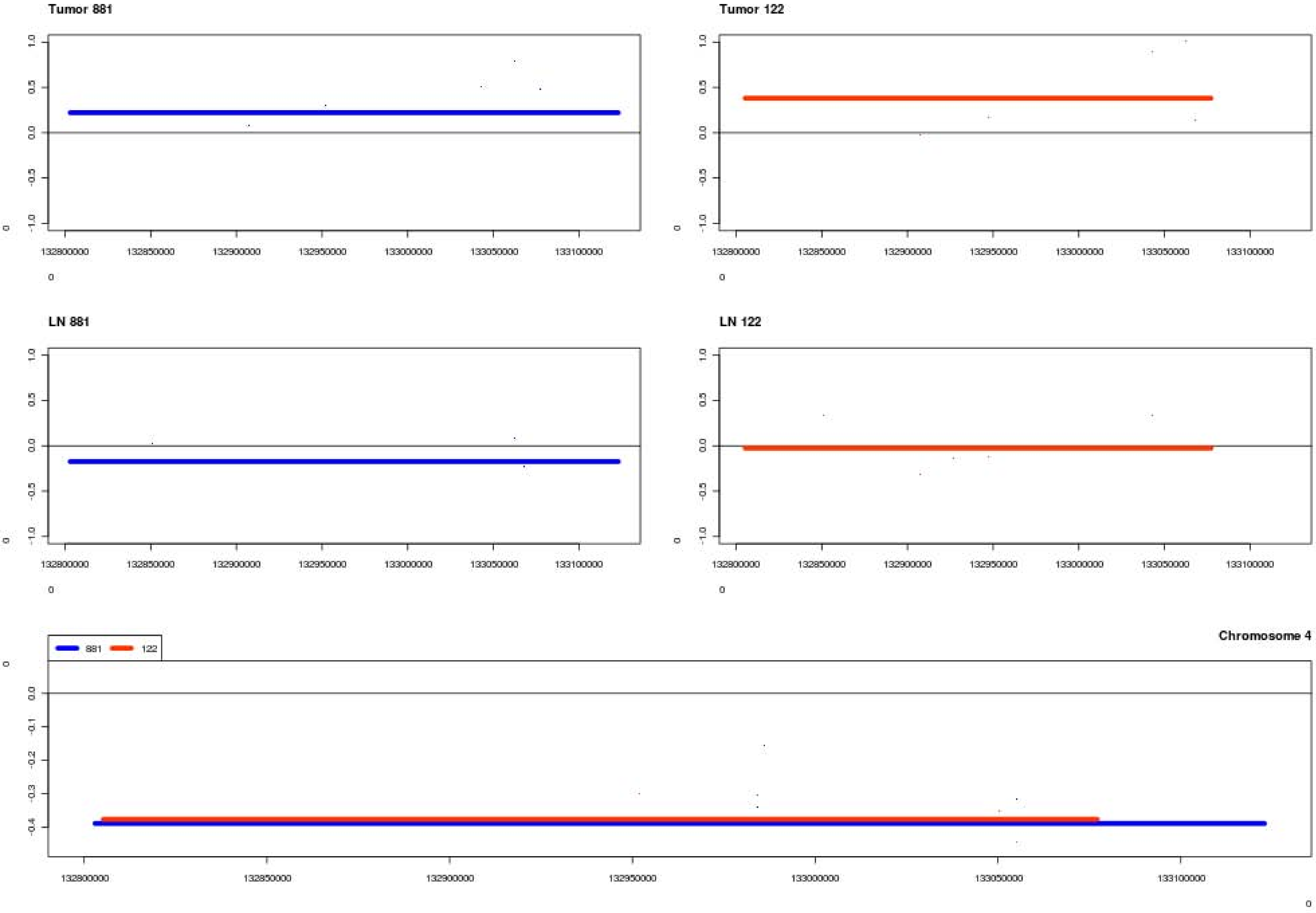

Tumor 881
Tumor 122
LN 881
LN 122
881
122
Chromosome 4
1.0
0.5
0.0
-0.5
-1.0
-0.1
-0.2
-0.3
-0.4
132800000
132850000
132900000
132950000
133000000
133050000
133100000

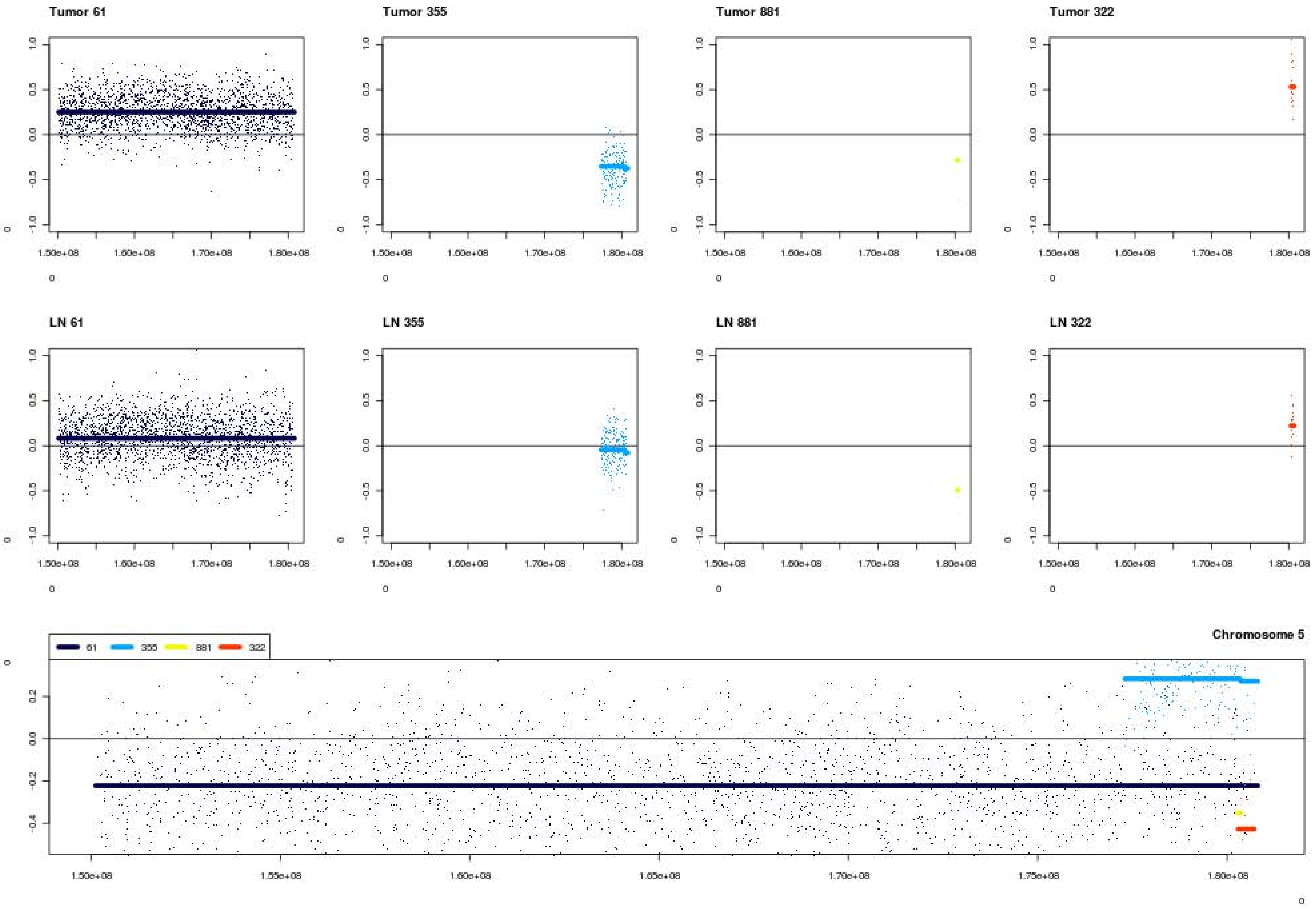
Tumor 61
Tumor 355
Tumor 881
Tumor 322
LN 61
LN 355
LN 881
LN 322
61
355
881
322
Chromosome 5
1.50e+08
1.55e+08
1.60e+08
1.65e+08
1.70e+08
1.75e+08
1.80e+08

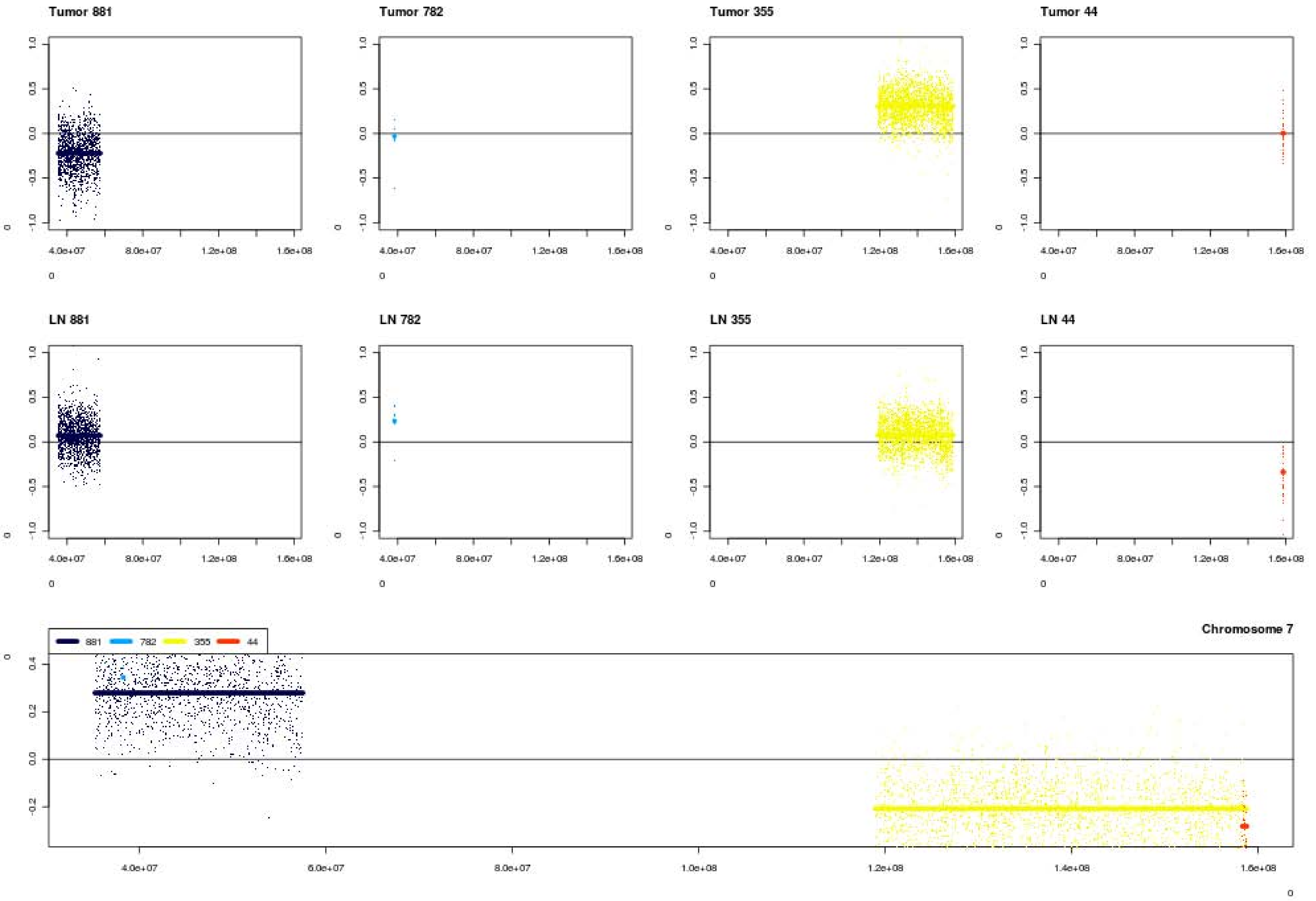

Tumor 881
Tumor 782
Tumor 355
Tumor 44
LN 881
LN 782
LN 355
LN 44
Chromosome 7
881
782
355
44

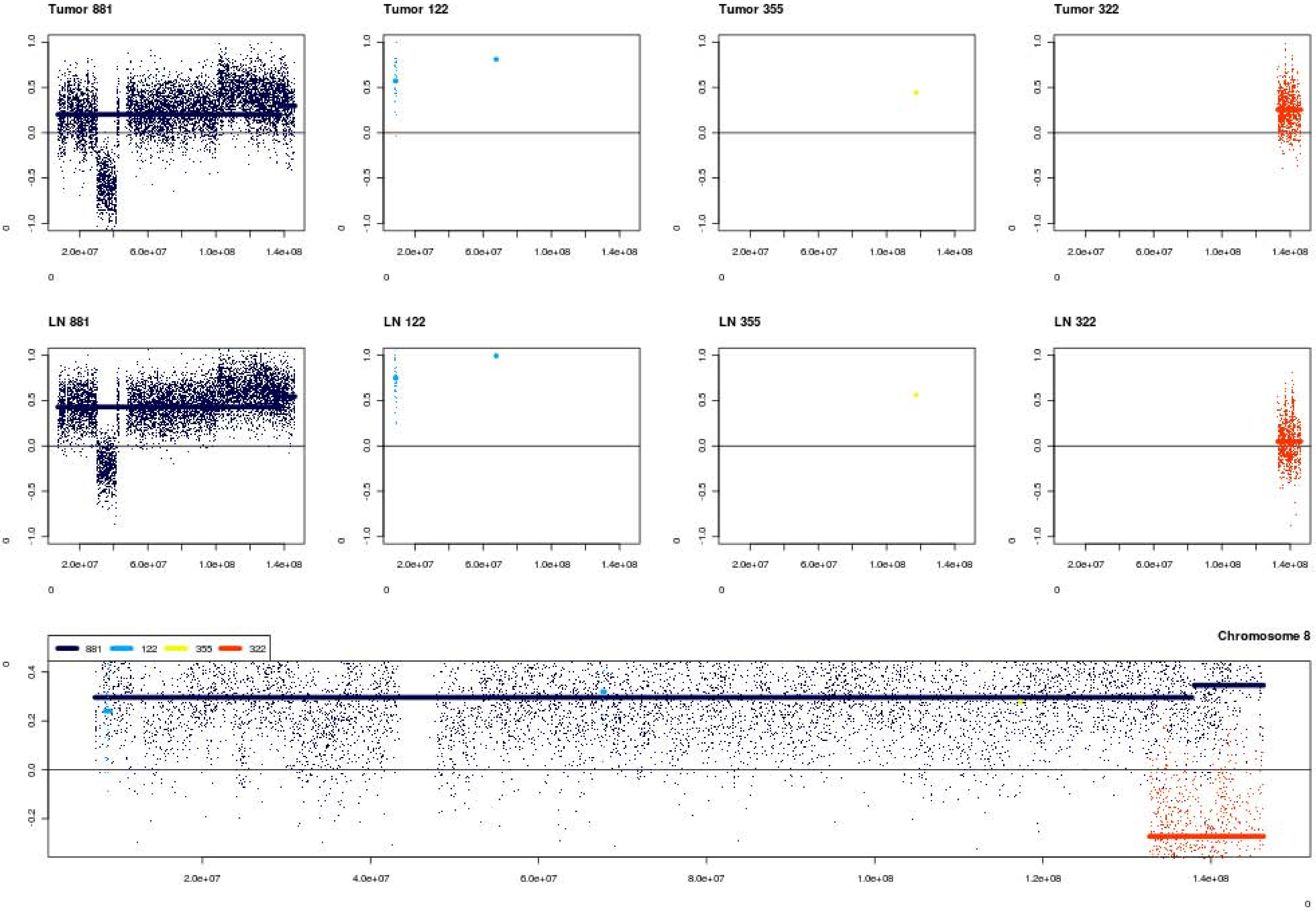

Tumor 881
Tumor 122
Tumor 355
Tumor 322
LN 881
LN 122
LN 355
LN 322
881
122
355
322
Chromosome 8

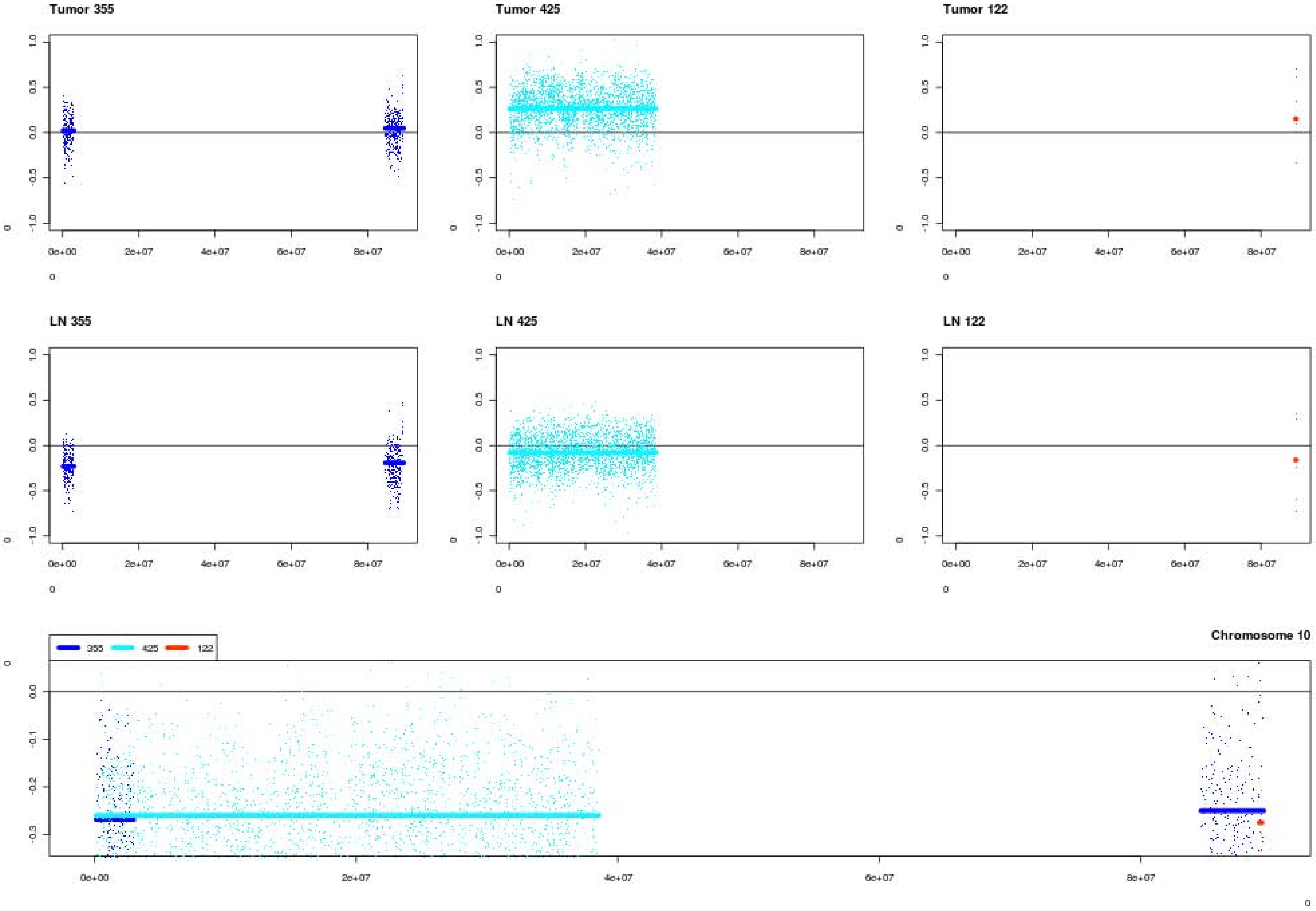
Tumor 355
Tumor 425
Tumor 122
LN 355
LN 425
LN 122
355
425
122
Chromosome 10

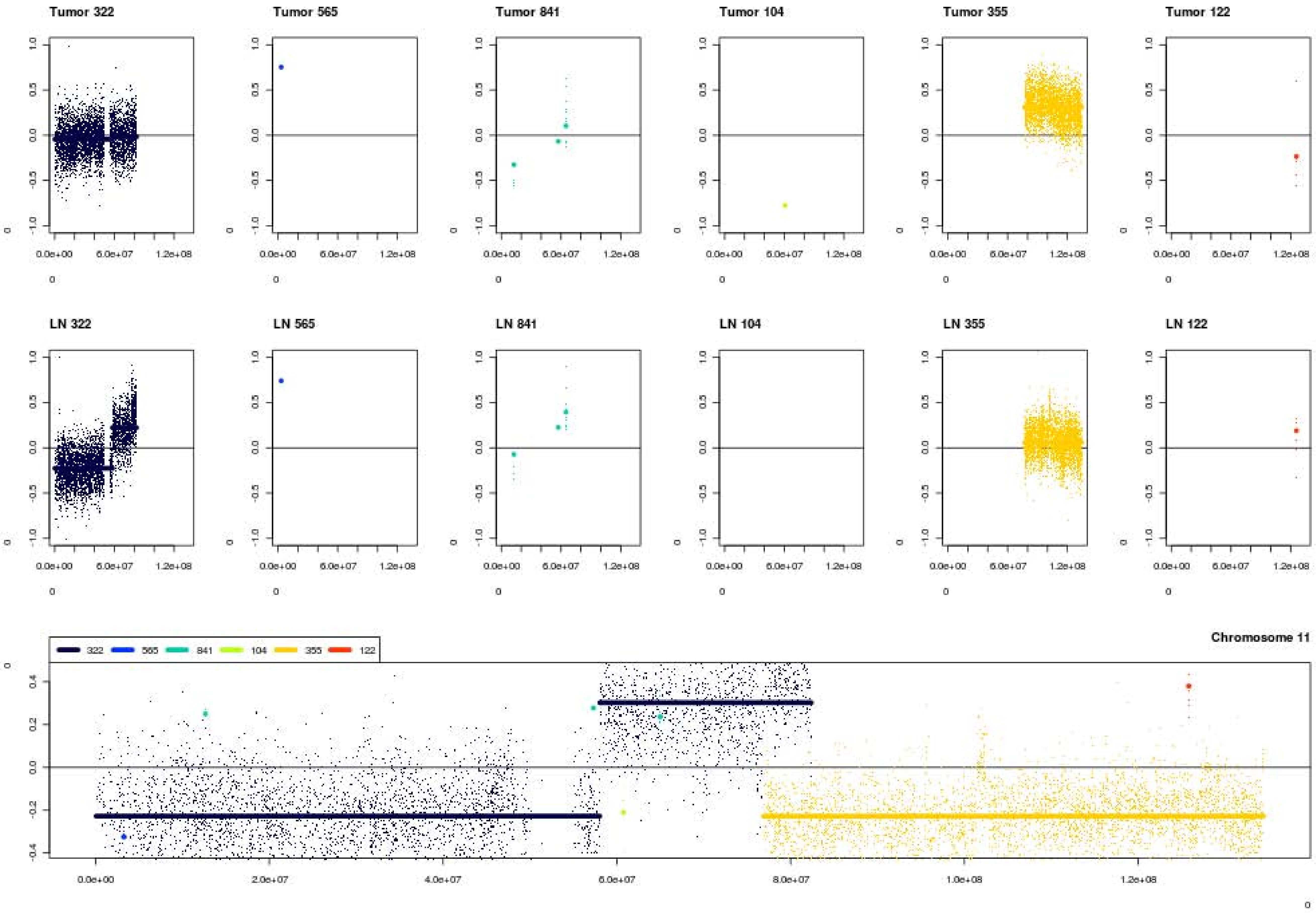
Tumor 322
Tumor 565
Tumor 841
Tumor 104
Tumor 355
Tumor 122
LN 322
LN 565
LN 841
LN 104
LN 355
LN 122
Chromosome 11
322
565
841
104
355
122

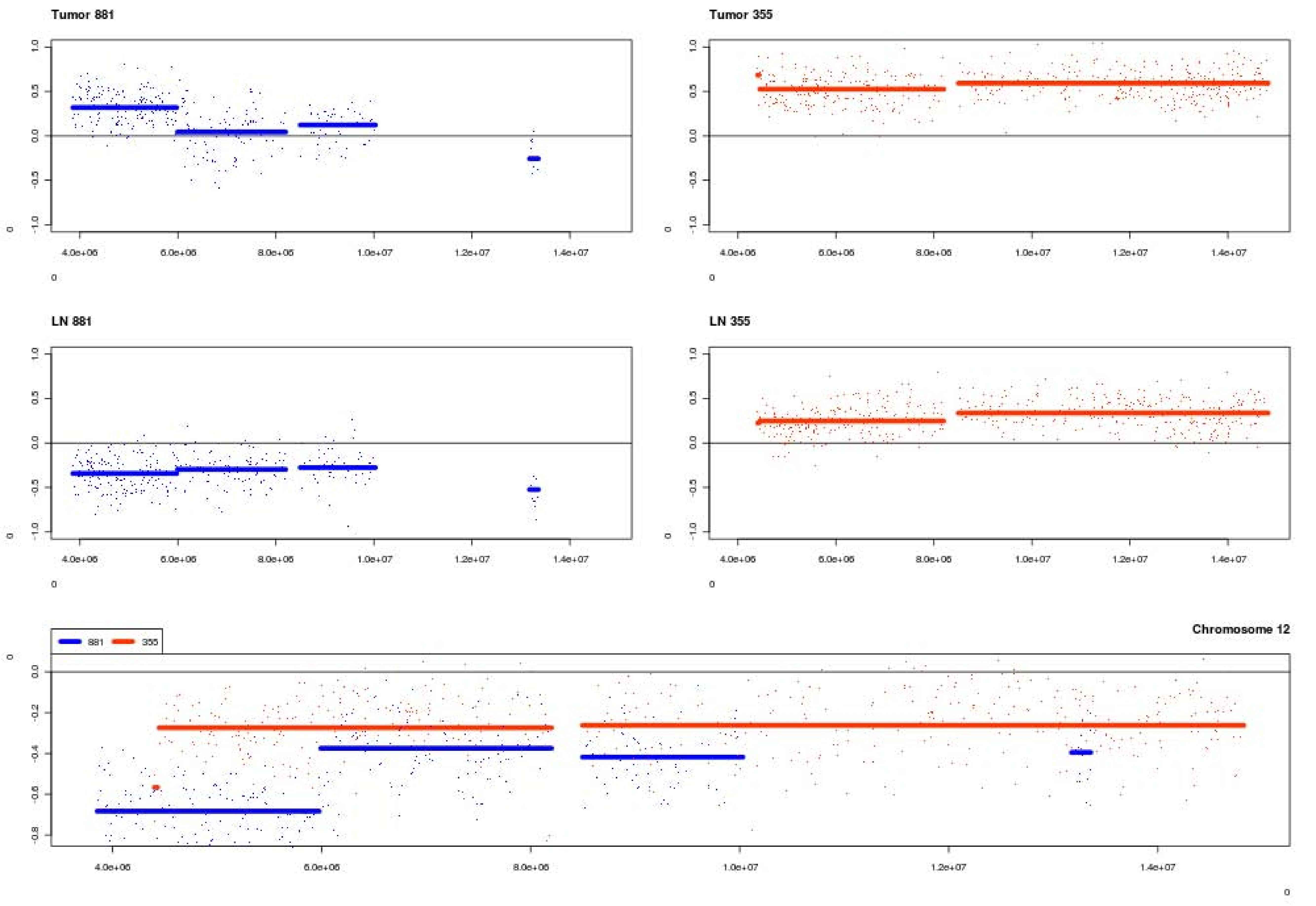

Tumor 881
Tumor 355
LN 881
LN 355
881
355
Chromosome 12
4.0e+06
6.0e+06
8.0e+06
1.0e+07
1.2e+07
1.4e+07

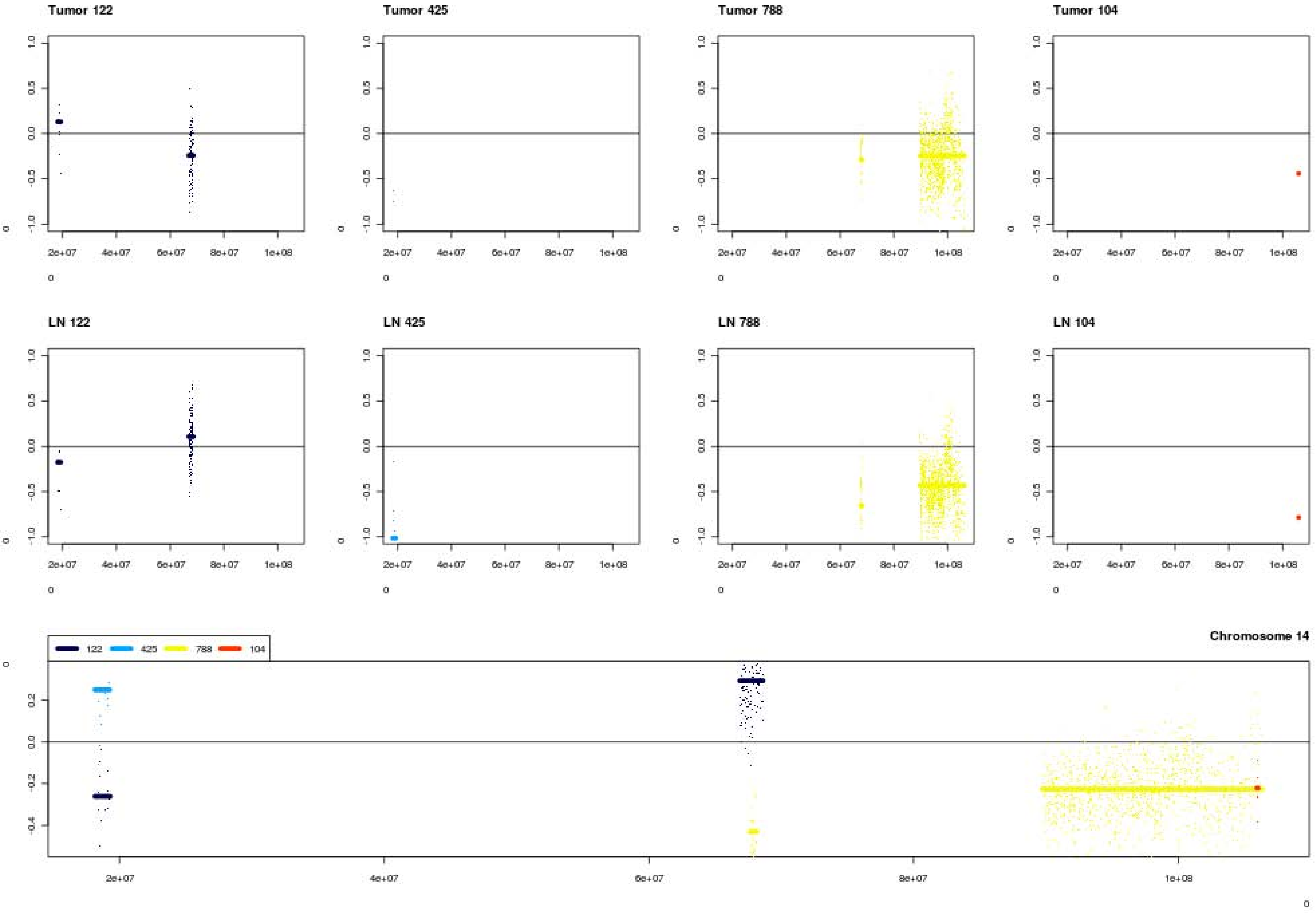

Tumor 122
Tumor 425
Tumor 788
Tumor 104
LN 122
LN 425
LN 788
LN 104
1.0
0.5
0.0
-0.5
-1.0
2e+07
4e+07
6e+07
8e+07
1e+08
122
425
788
104
Chromosome 14
0.2
0.0
-0.2
-0.4

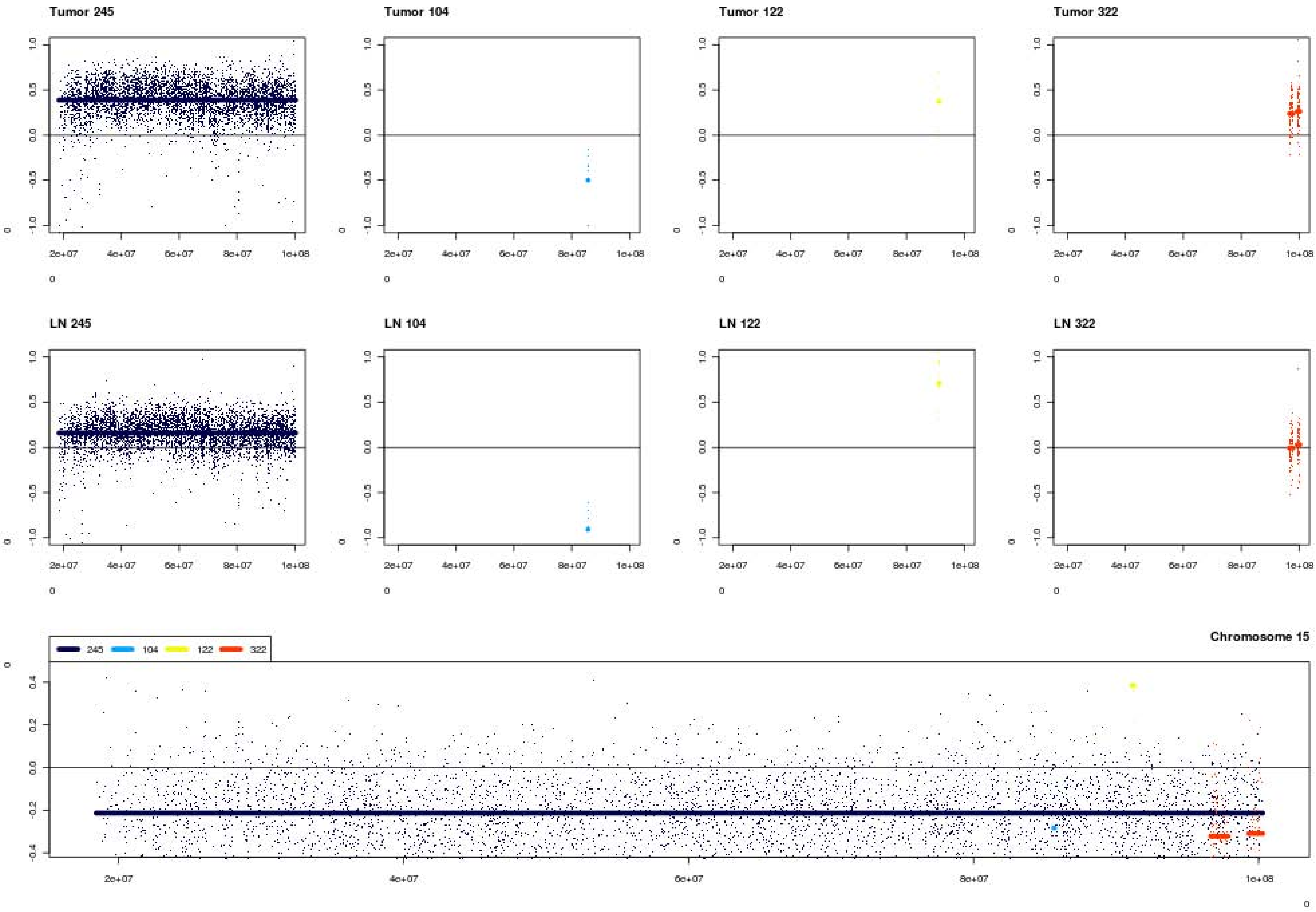

Tumor 245
Tumor 104
Tumor 122
Tumor 322
LN 245
LN 104
LN 122
LN 322
245
104
122
322
Chromosome 15

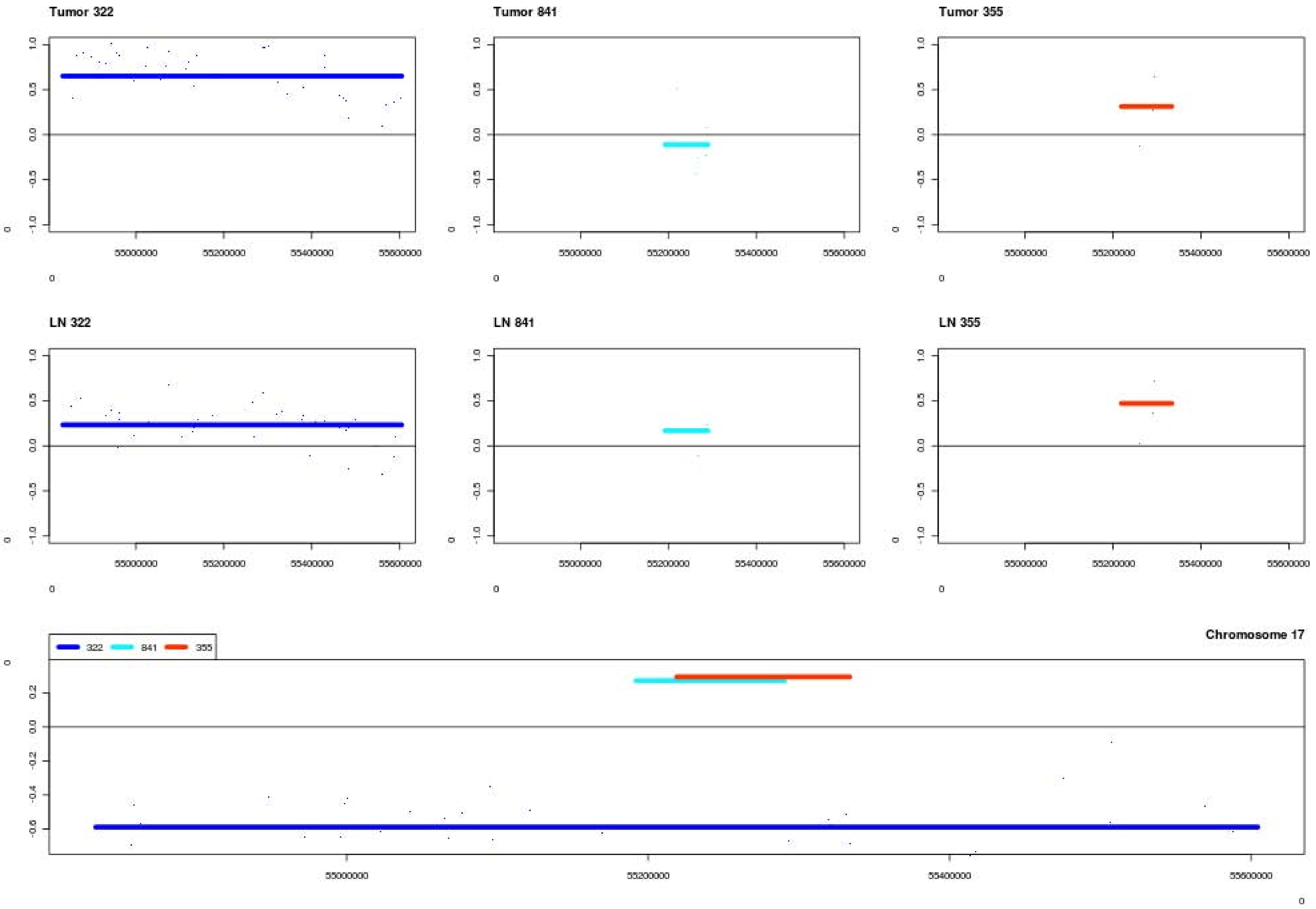

Tumor 322
Tumor 841
Tumor 355
LN 322
LN 841
LN 355
322
841
355
Chromosome 17
55000000
55200000
55400000
55600000

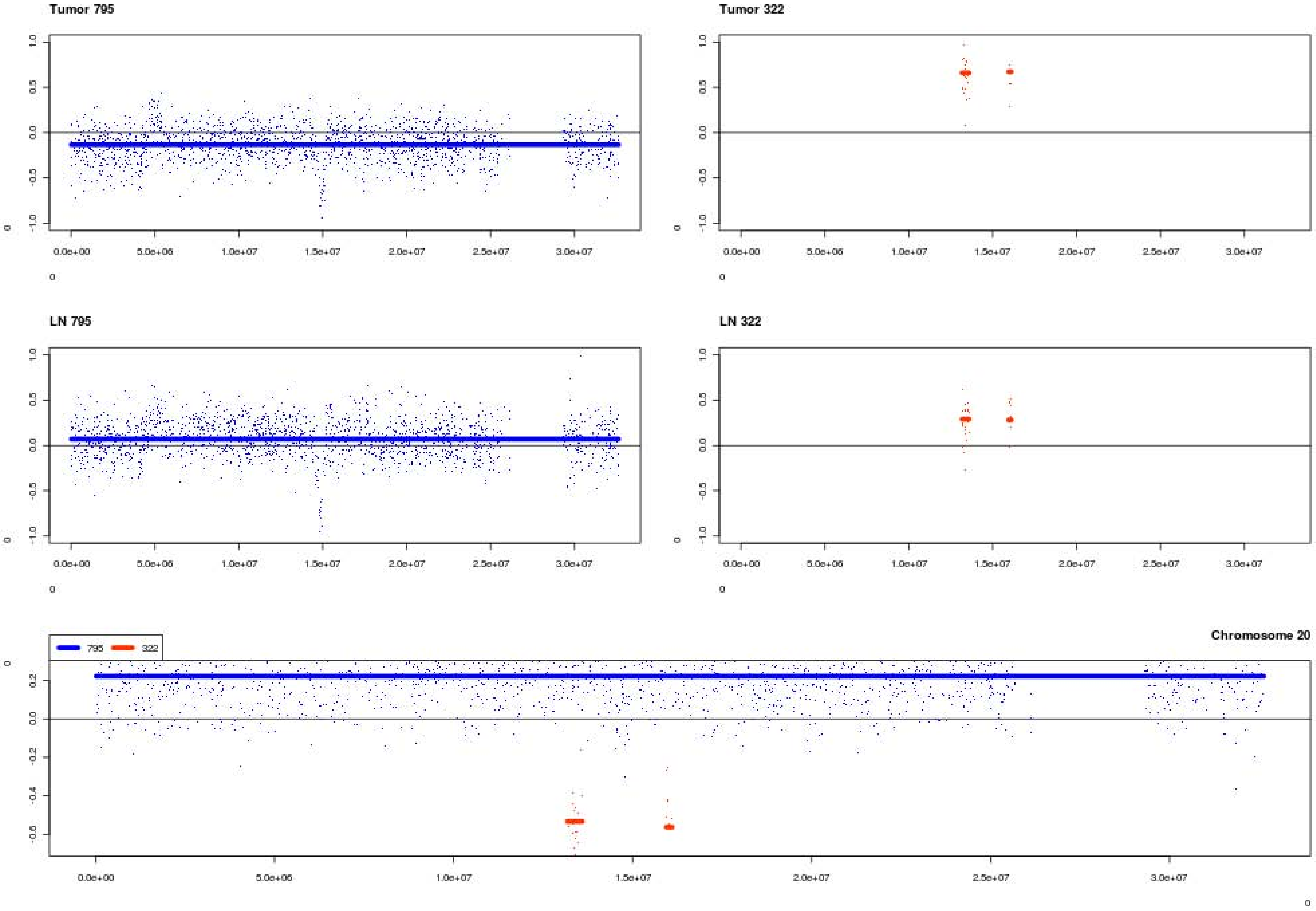

Tumor 795
Tumor 322
LN 795
LN 322
Chromosome 20
795
322
1.0
0.5
0.0
-0.5
-1.0
0.2
-0.2
-0.4
-0.6
0.0e+00
5.0e+06
1.0e+07
1.5e+07
2.0e+07
2.5e+07
3.0e+07

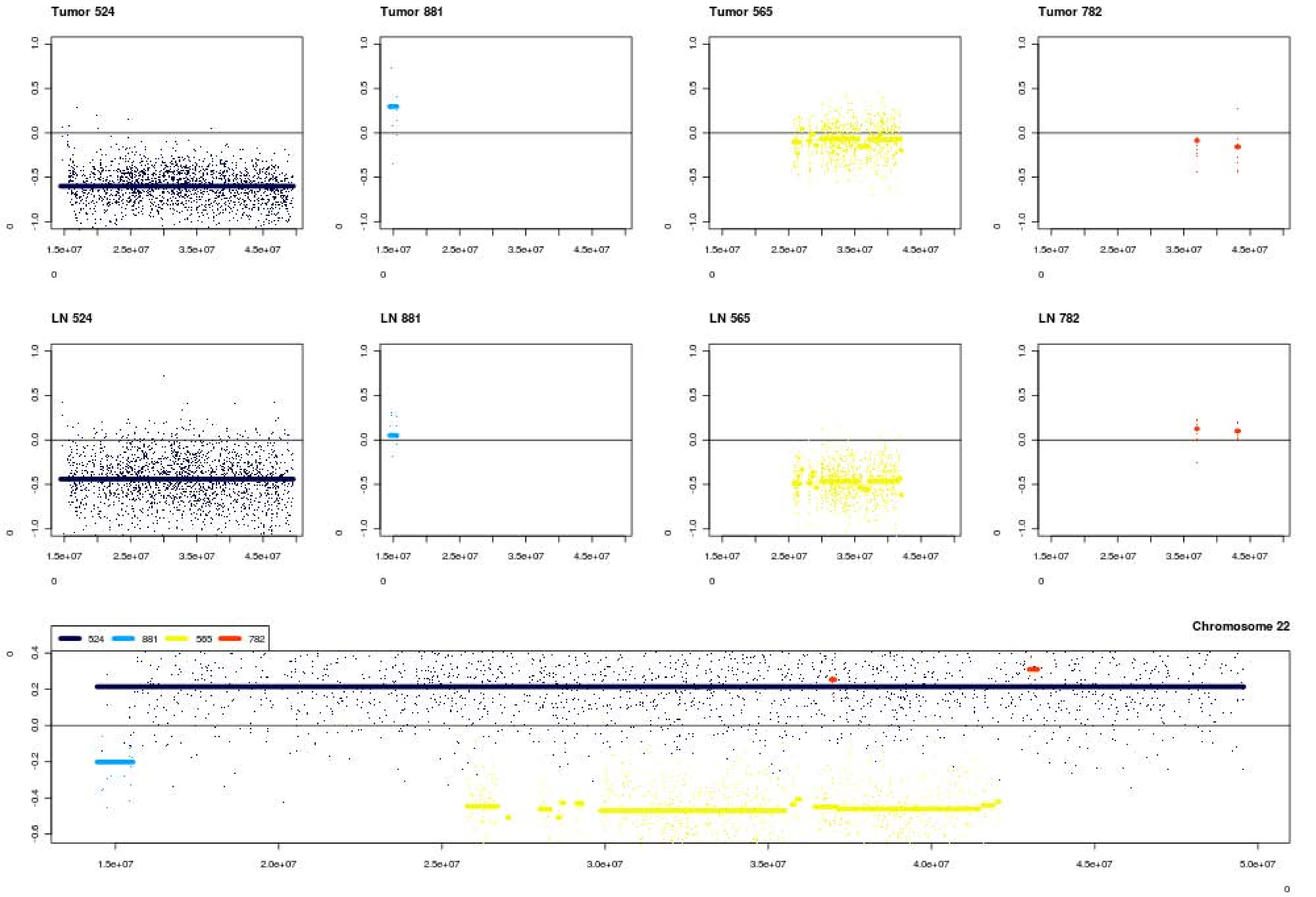
Tumor 524
Tumor 881
Tumor 565
Tumor 782
LN 524
LN 881
LN 565
LN 782
1.0
0.5
0.0
-0.5
-1.0
1.5e+07
2.5e+07
3.5e+07
4.5e+07
0
524
881
565
782
Chromosome 22
0.4
0.2
0.0
-0.2
-0.4
-0.6
1.5e+07
2.0e+07
2.5e+07
3.0e+07
3.5e+07
4.0e+07
4.5e+07
5.0e+07

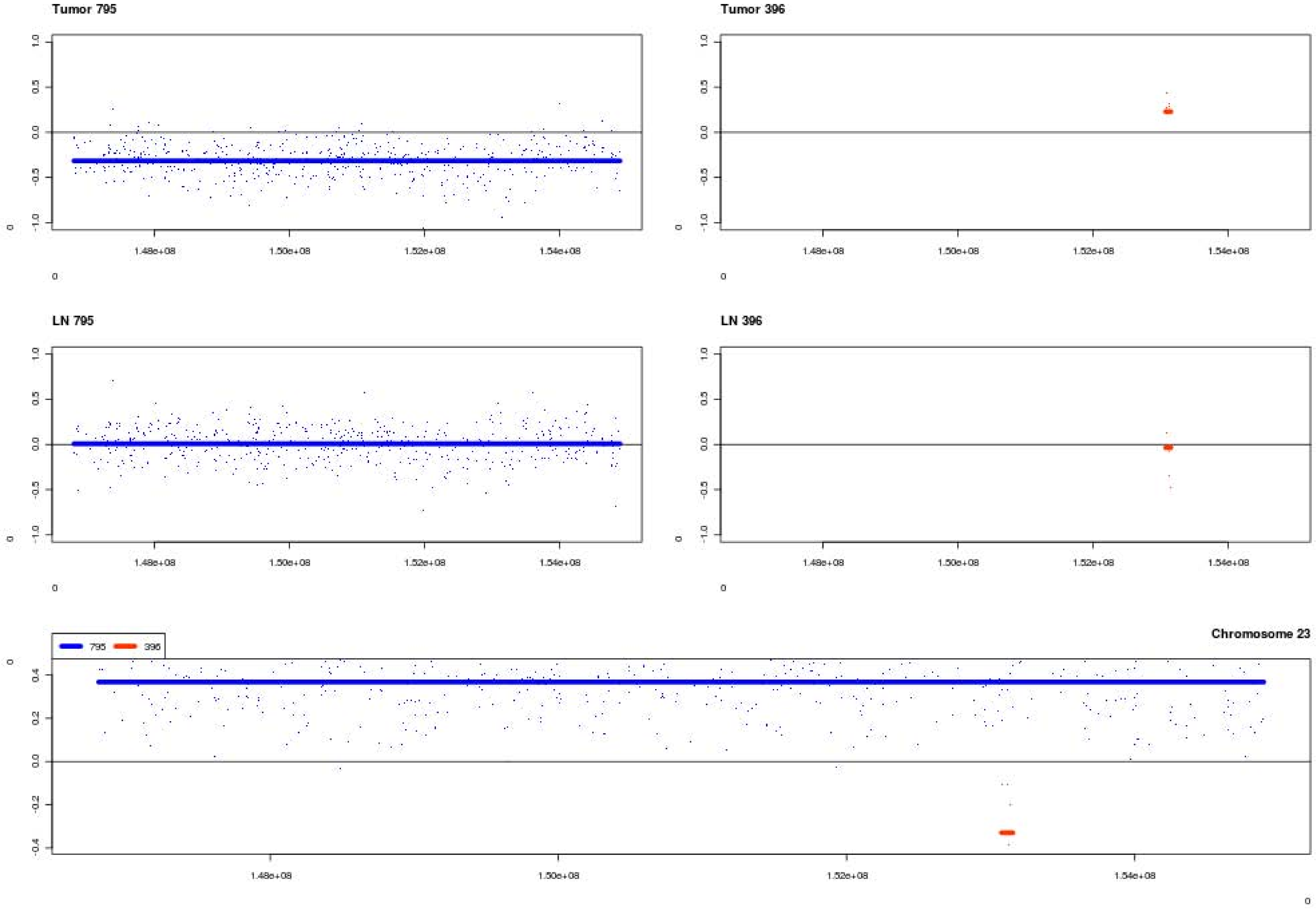
Tumor 795
Tumor 396
LN 795
LN 396
Chromosome 23
795
396
1.48e+08
1.50e+08
1.52e+08
1.54e+08
1.0
0.5
0.0
-0.5
-1.0
0.4
0.2
-0.2
-0.4

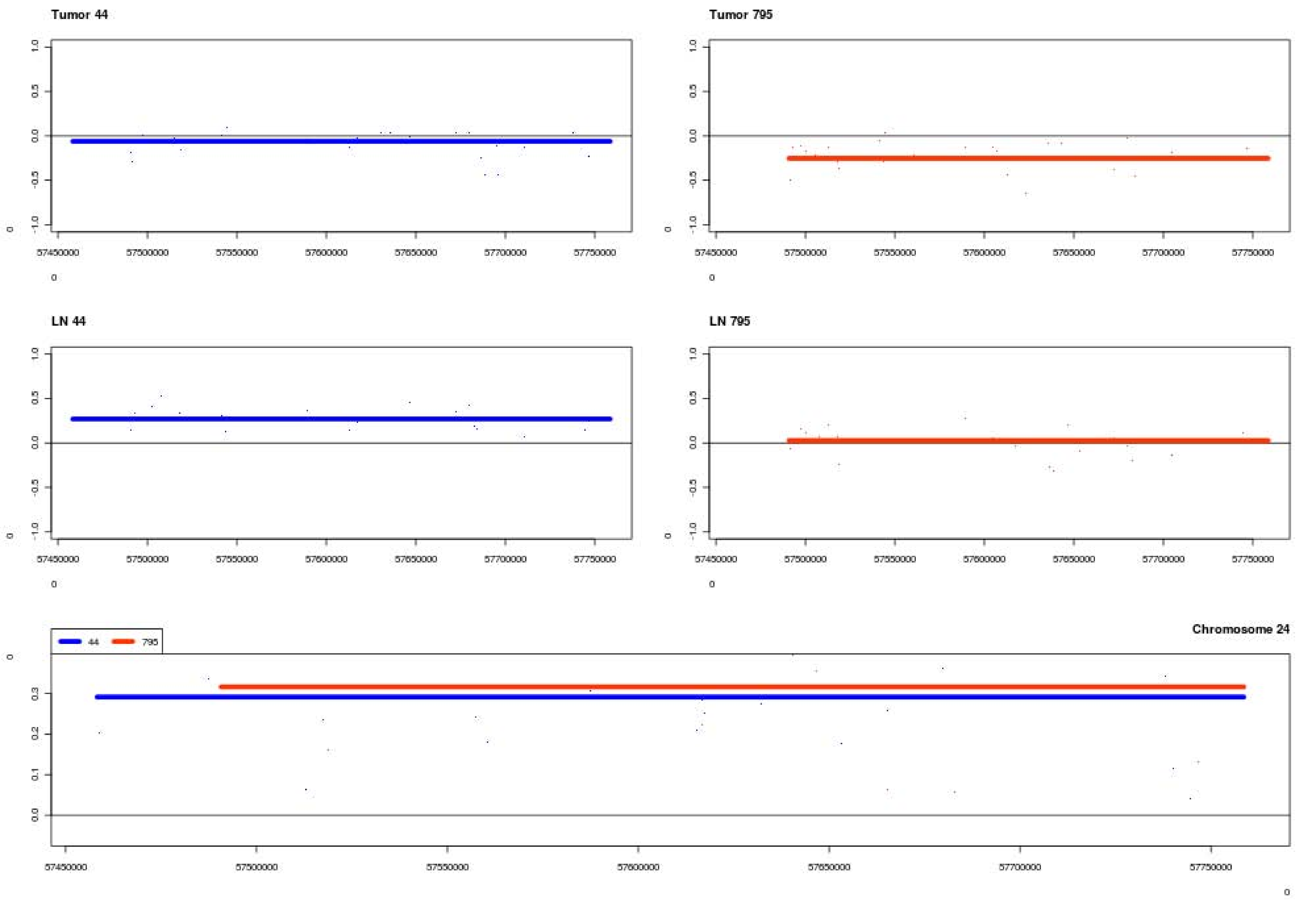
Tumor 44
Tumor 795
LN 44
LN 795
44
795
Chromosome 24
