# Supplementary material for: Lack of Genomic Heterogeneity at High-Resolution aCGH between Primary Breast Cancers and Their Paired Lymph Node Metastases
Source: PLoS One. 2014 Aug 1;9(8):e103177. doi: 10.1371/journal.pone.0103177 (PMC4118860; doi:10.1371/journal.pone.0103177)
Supplement: File S3 — Visualizations of the 135 K copy number profile of all primary tumour – lymph node metastasis pairs that were included in follow-up analyses. Each page shows the profiles for a single patient. (PDF) [file pone.0103177.s005.pdf]

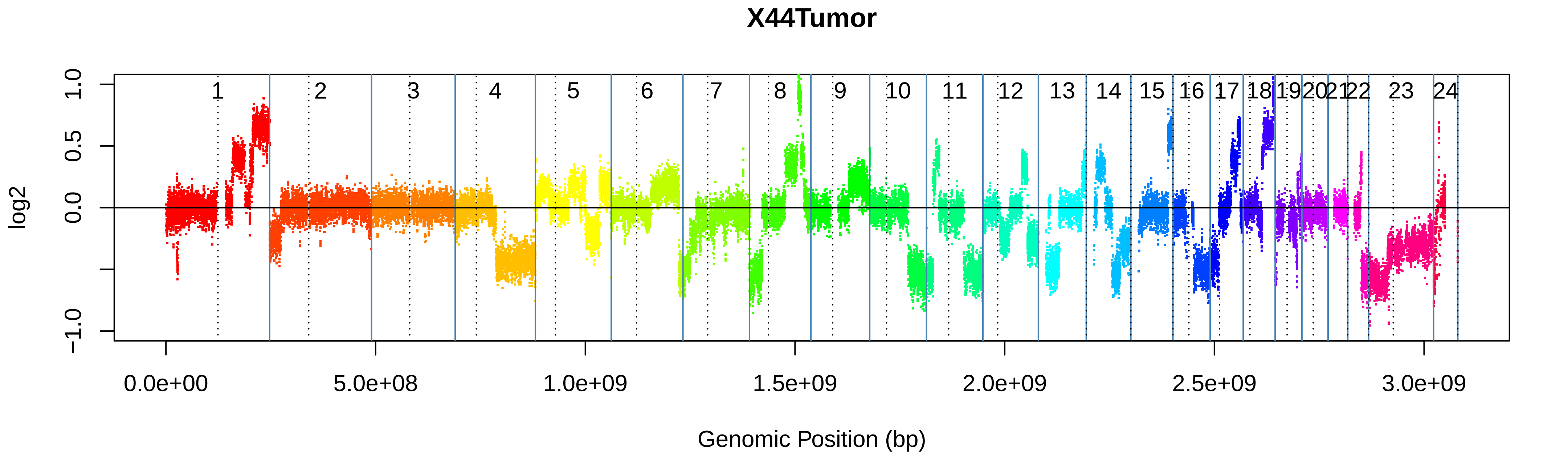
X44Tumor
log2
1.0
0.5
0.0
−1.0
1 2 3 4 5 6 7 8 9 10 11 12 13 14 15 16 17 18 19 20 21 22 23 24
0.0e+00
5.0e+08
1.0e+09
1.5e+09
2.0e+09
2.5e+09
3.0e+09
Genomic Position (bp)

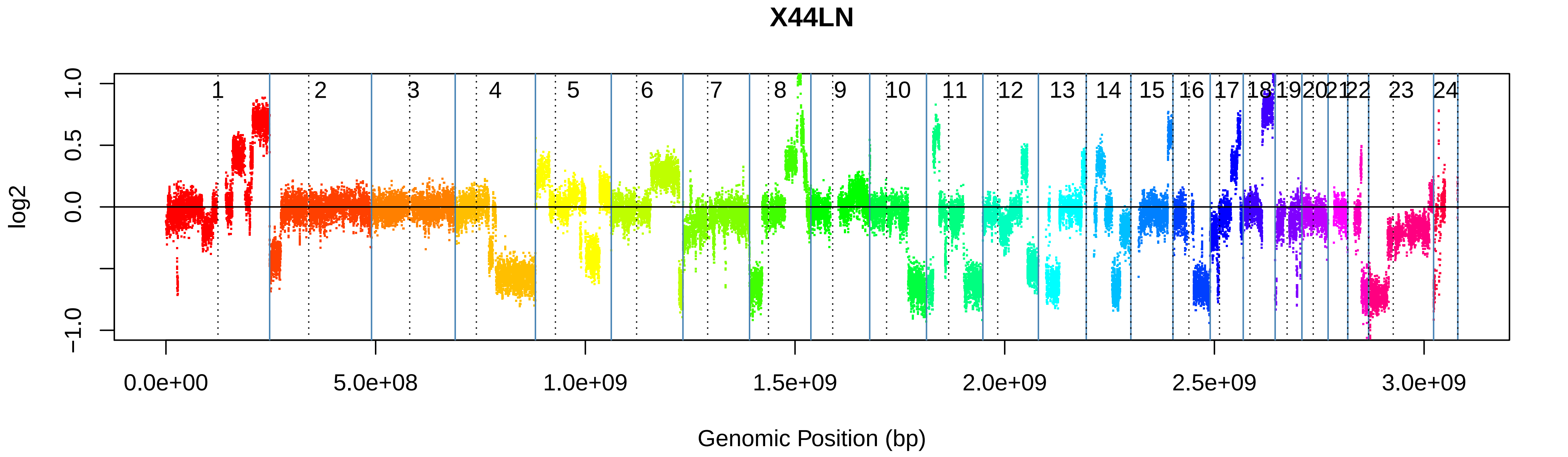
X44LN
log2
1.0
0.5
0.0
−1.0
1 2 3 4 5 6 7 8 9 10 11 12 13 14 15 16 17 18 19 20 21 22 23 24
0.0e+00
5.0e+08
1.0e+09
1.5e+09
2.0e+09
2.5e+09
3.0e+09
Genomic Position (bp)

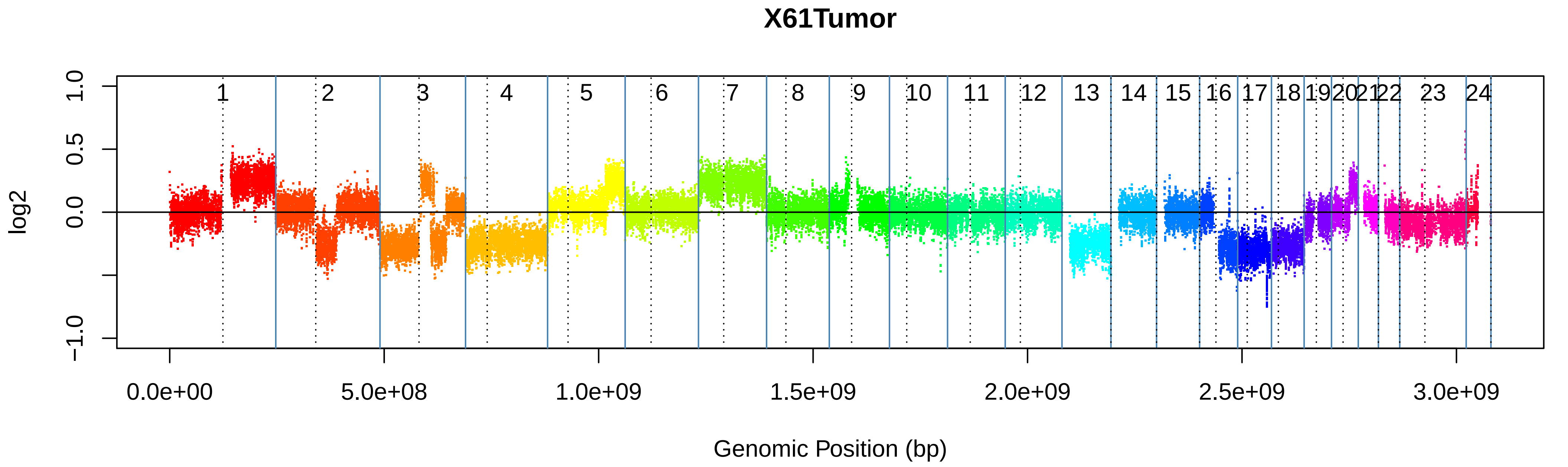
X61Tumor
log2
1.0
0.5
0.0
−1.0
1
2
3
4
5
6
7
8
9
10
11
12
13
14
15
16
17
18
19
20
21
22
23
24
0.0e+00
5.0e+08
1.0e+09
1.5e+09
2.0e+09
2.5e+09
3.0e+09
Genomic Position (bp)

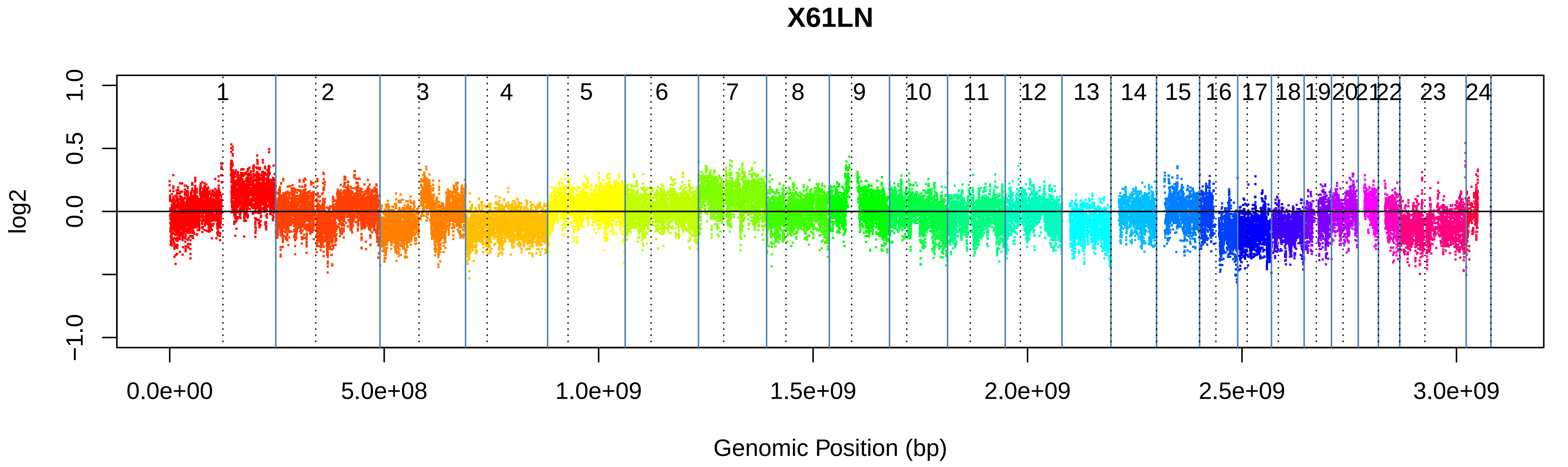
X61LN
log2
1.0
0.5
0.0
−1.0
1
2
3
4
5
6
7
8
9
10
11
12
13
14
15
16
17
18
19
20
21
22
23
24
0.0e+00
5.0e+08
1.0e+09
1.5e+09
2.0e+09
2.5e+09
3.0e+09
Genomic Position (bp)

X104Tumor

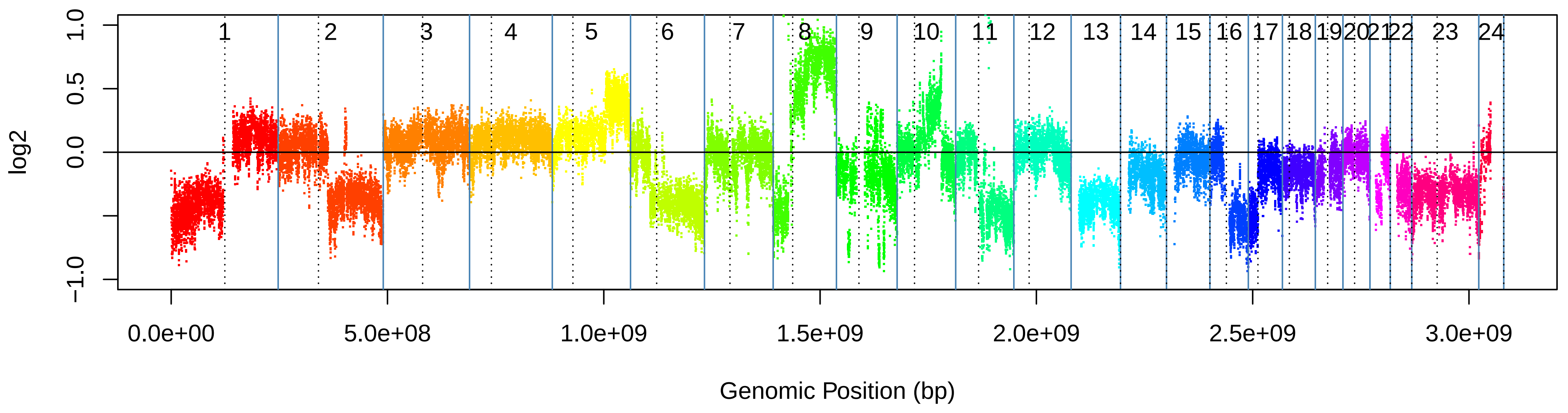

X104LN

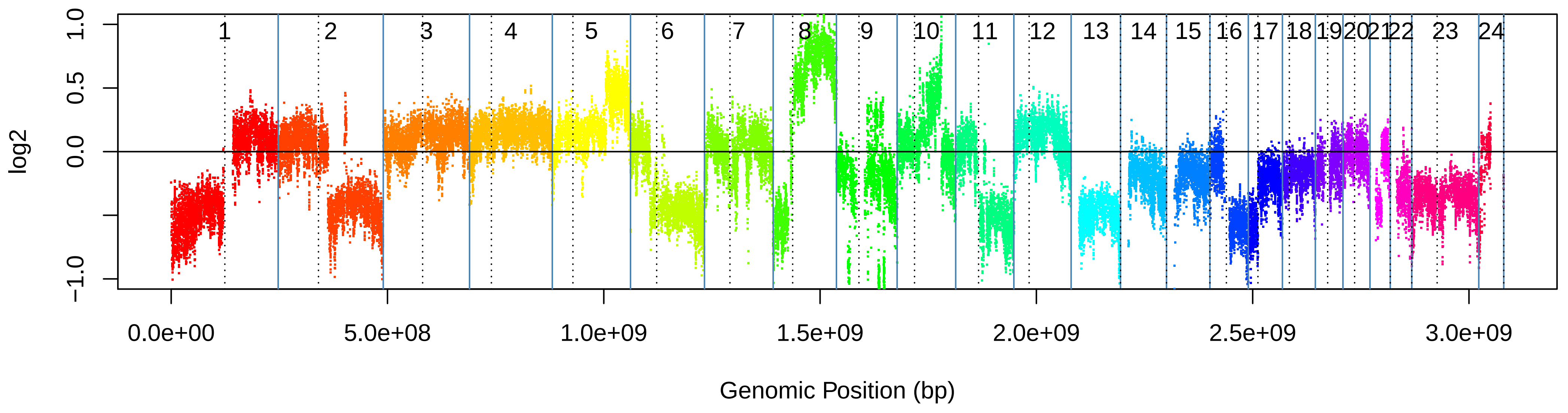

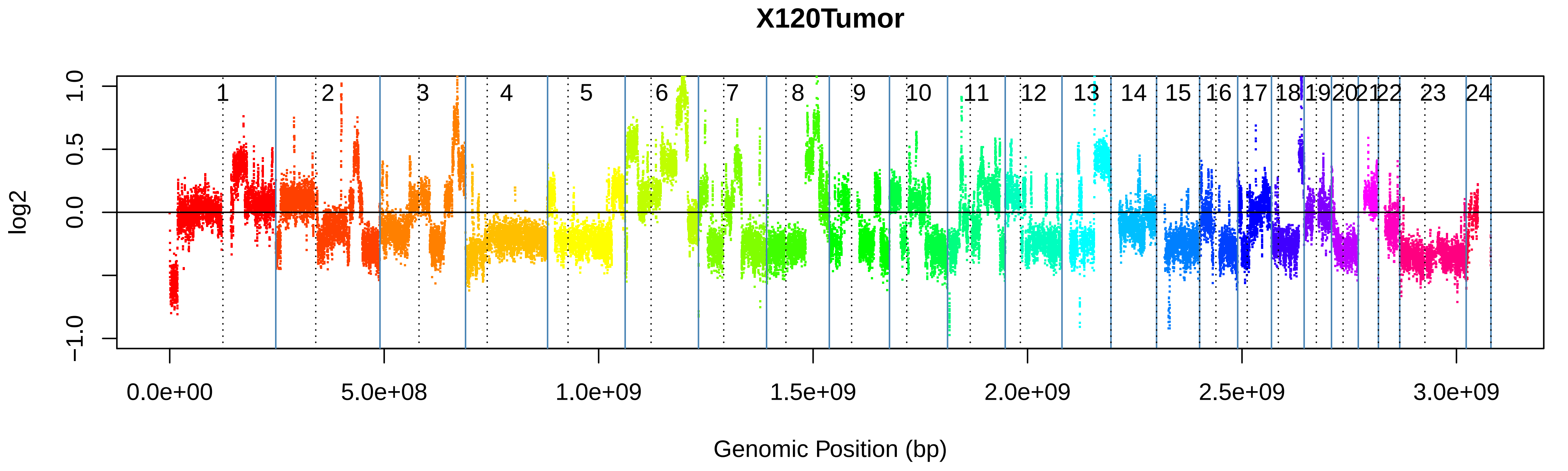
X120Tumor
log2
1.0
0.5
0.0
−1.0
1
2
3
4
5
6
7
8
9
10
11
12
13
14
15
16
17
18
19
20
21
22
23
24
0.0e+00
5.0e+08
1.0e+09
1.5e+09
2.0e+09
2.5e+09
3.0e+09
Genomic Position (bp)

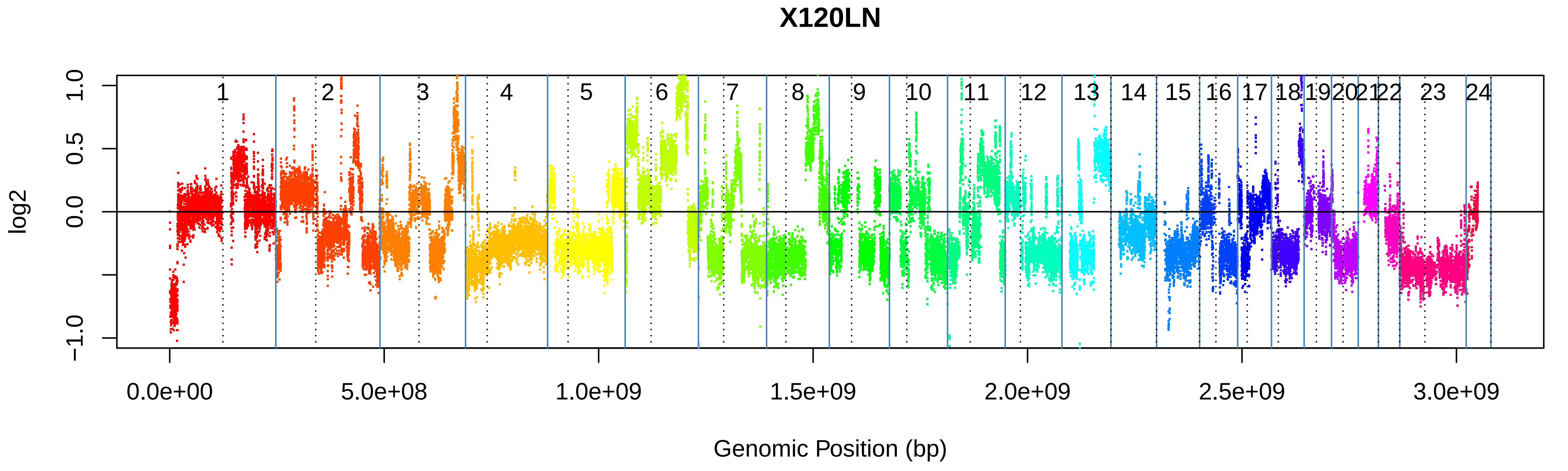
X120LN
log2
1.0
0.5
0.0
−1.0
1
2
3
4
5
6
7
8
9
10
11
12
13
14
15
16
17
18
19
20
21
22
23
24
0.0e+00
5.0e+08
1.0e+09
1.5e+09
2.0e+09
2.5e+09
3.0e+09
Genomic Position (bp)

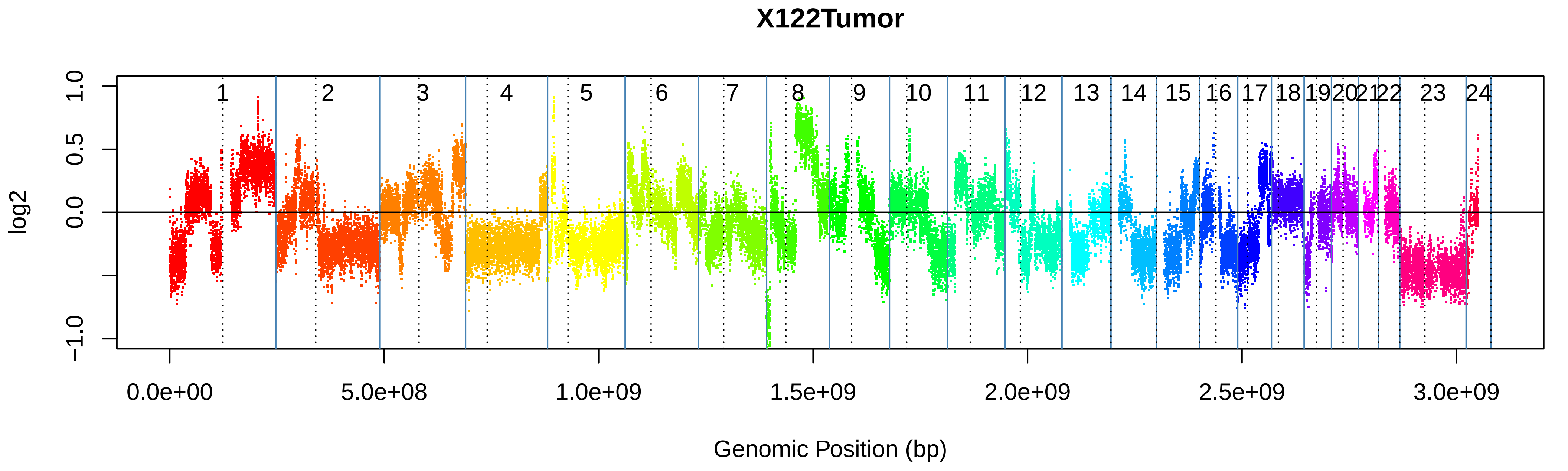
X122Tumor
log2
1.0
0.5
0.0
−1.0
1
2
3
4
5
6
7
8
9
10
11
12
13
14
15
16
17
18
19
20
21
22
23
24
0.0e+00
5.0e+08
1.0e+09
1.5e+09
2.0e+09
2.5e+09
3.0e+09
Genomic Position (bp)

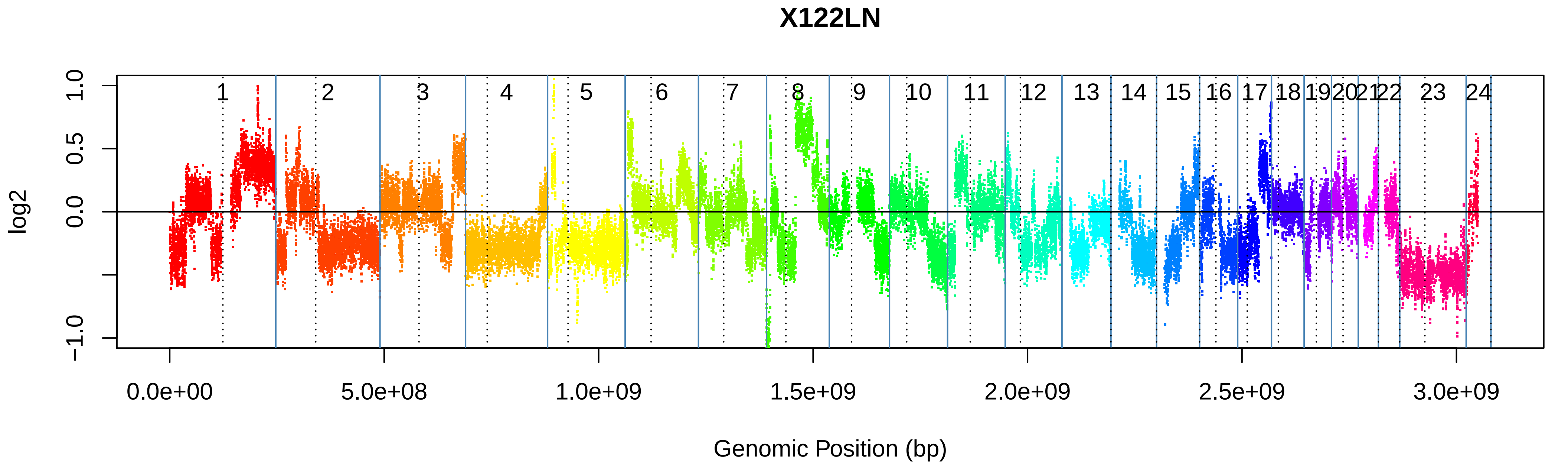
X122LN
log2
1.0
0.5
0.0
−1.0
1
2
3
4
5
6
7
8
9
10
11
12
13
14
15
16
17
18
19
20
21
22
23
24
0.0e+00
5.0e+08
1.0e+09
1.5e+09
2.0e+09
2.5e+09
3.0e+09
Genomic Position (bp)

X229Tumor

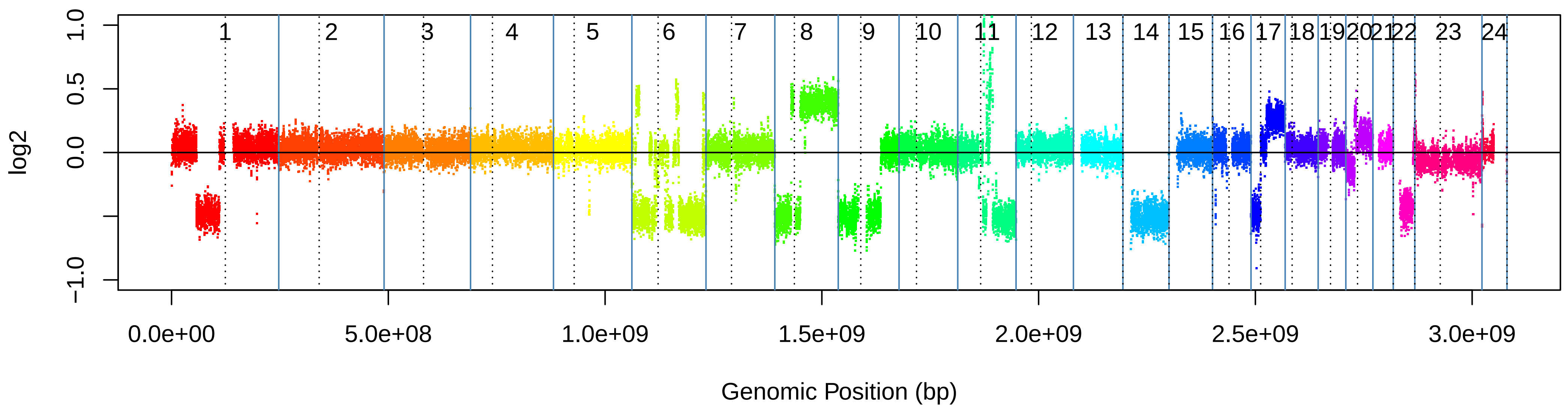

X229LN

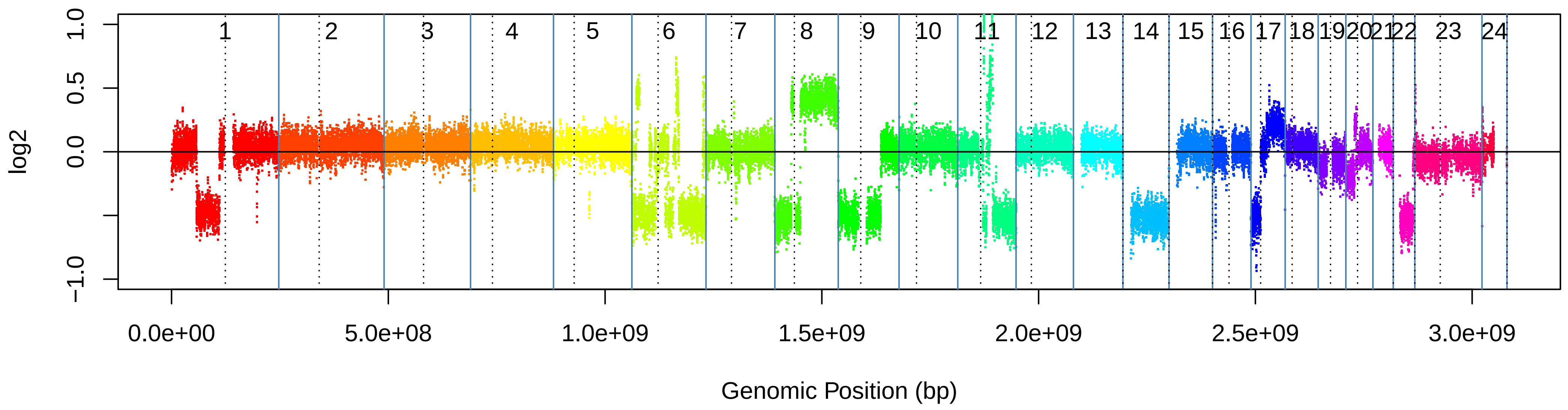

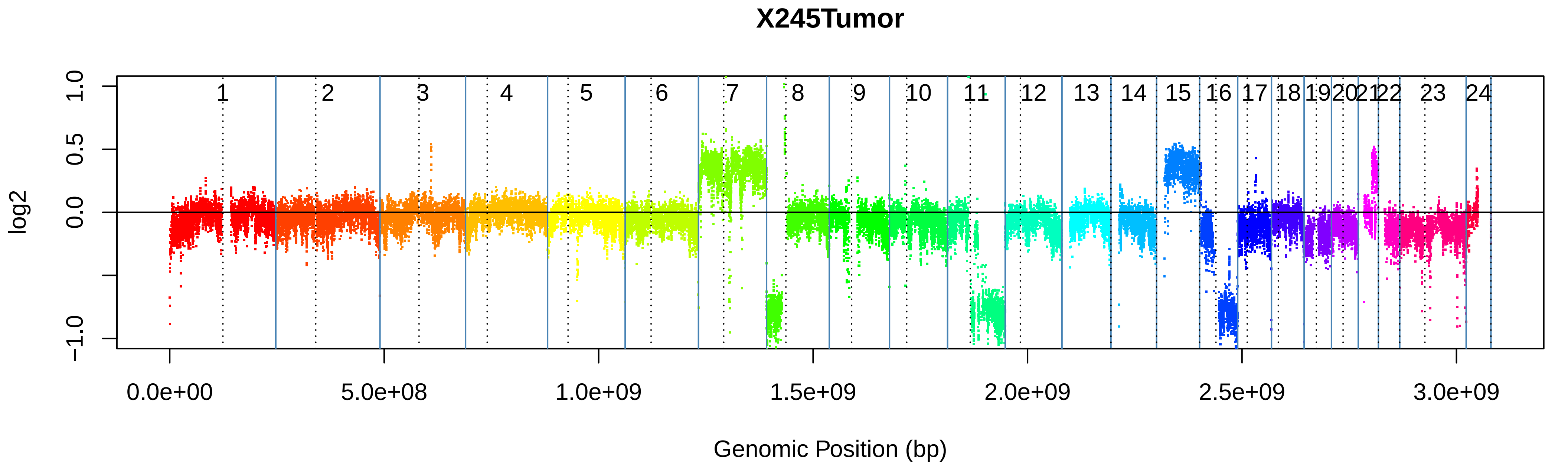
X245Tumor
log2
1.0
0.5
0.0
−1.0
1
2
3
4
5
6
7
8
9
10
11
12
13
14
15
16
17
18
19
20
21
22
23
24
0.0e+00
5.0e+08
1.0e+09
1.5e+09
2.0e+09
2.5e+09
3.0e+09
Genomic Position (bp)

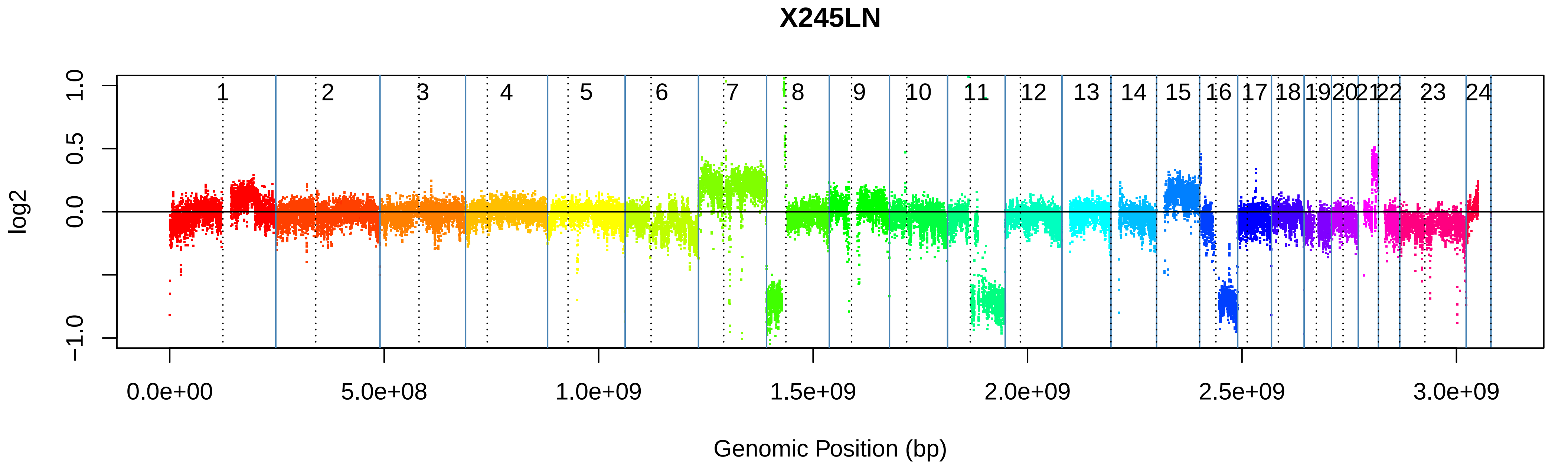
X245LN
log2
1.0
0.5
0.0
−1.0
1
2
3
4
5
6
7
8
9
10
11
12
13
14
15
16
17
18
19
20
21
22
23
24
0.0e+00
5.0e+08
1.0e+09
1.5e+09
2.0e+09
2.5e+09
3.0e+09
Genomic Position (bp)

X322Tumor

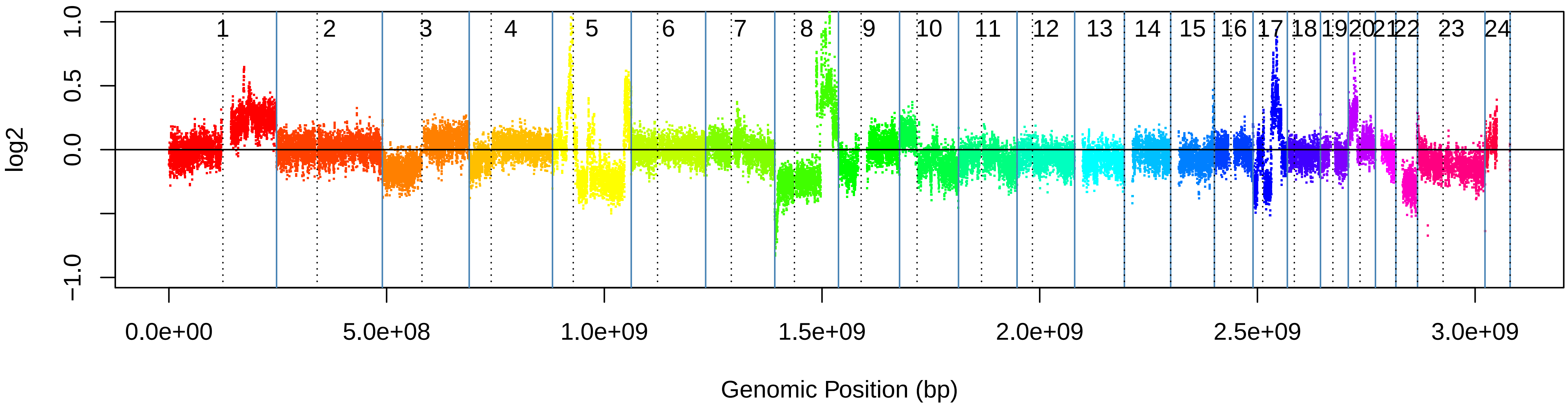

X322LN

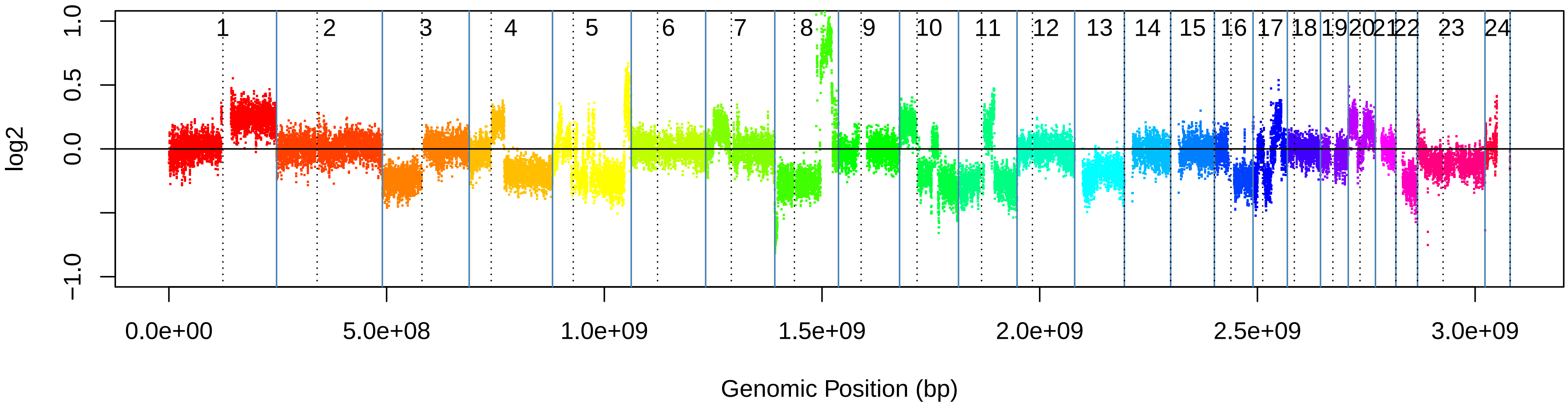

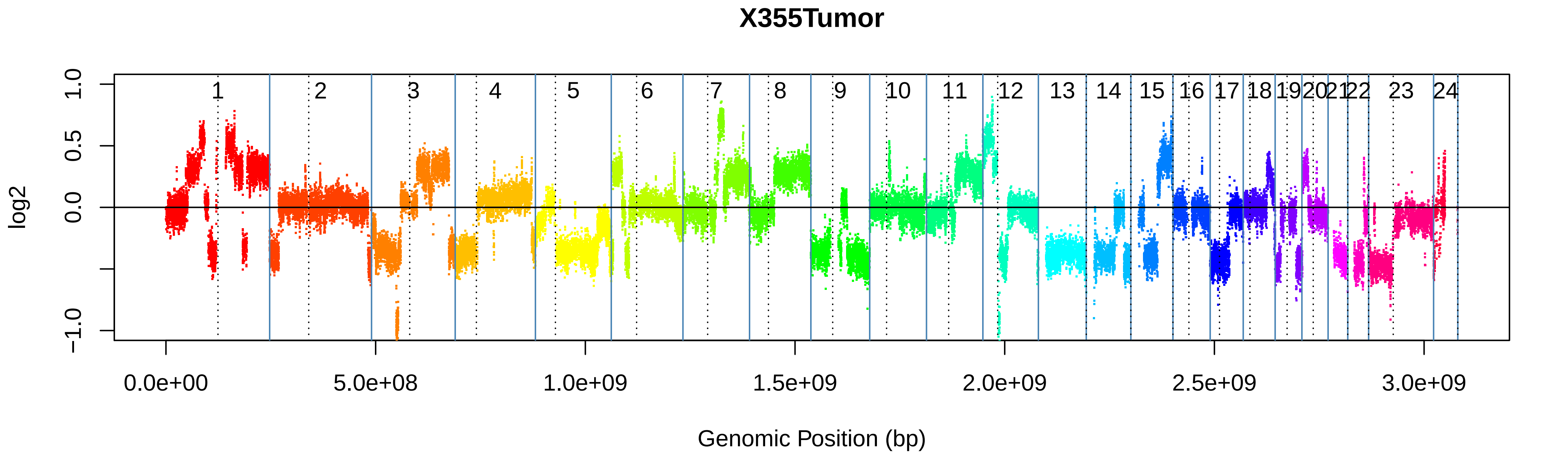
X355Tumor
log2
1.0
0.5
0.0
−1.0
1
2
3
4
5
6
7
8
9
10
11
12
13
14
15
16
17
18
19
20
21
22
23
24
0.0e+00
5.0e+08
1.0e+09
1.5e+09
2.0e+09
2.5e+09
3.0e+09
Genomic Position (bp)

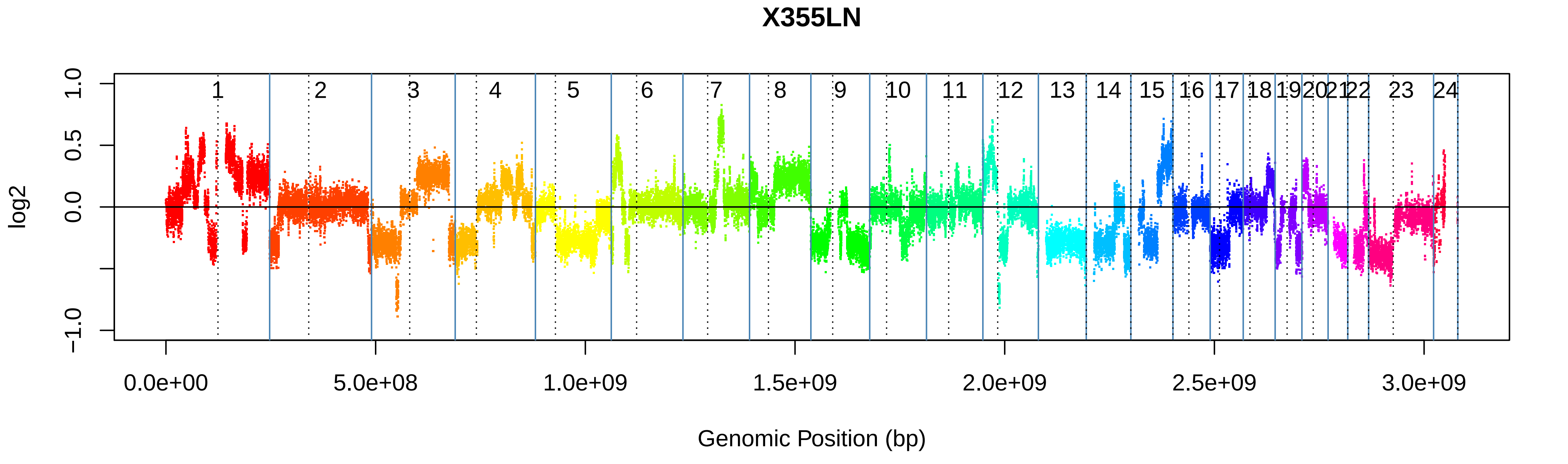
X355LN
log2
1.0
0.5
0.0
−1.0
1
2
3
4
5
6
7
8
9
10
11
12
13
14
15
16
17
18
19
20
21
22
23
24
0.0e+00
5.0e+08
1.0e+09
1.5e+09
2.0e+09
2.5e+09
3.0e+09
Genomic Position (bp)

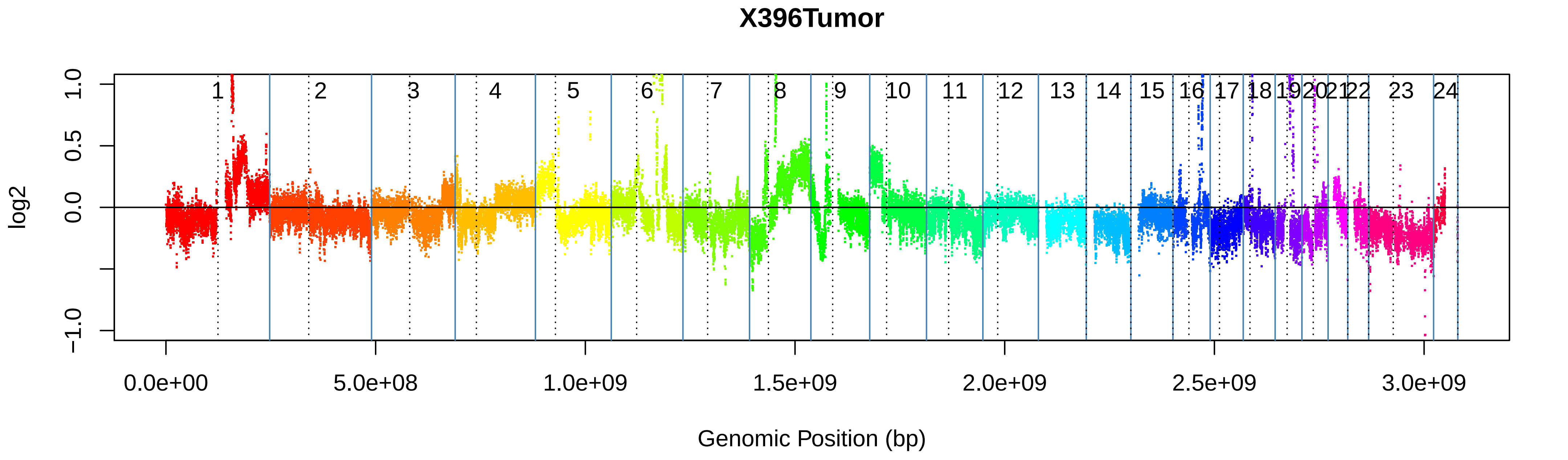
X396Tumor
log2
1.0
0.5
0.0
−1.0
1
2
3
4
5
6
7
8
9
10
11
12
13
14
15
16
17
18
19
20
21
22
23
24
0.0e+00
5.0e+08
1.0e+09
1.5e+09
2.0e+09
2.5e+09
3.0e+09
Genomic Position (bp)

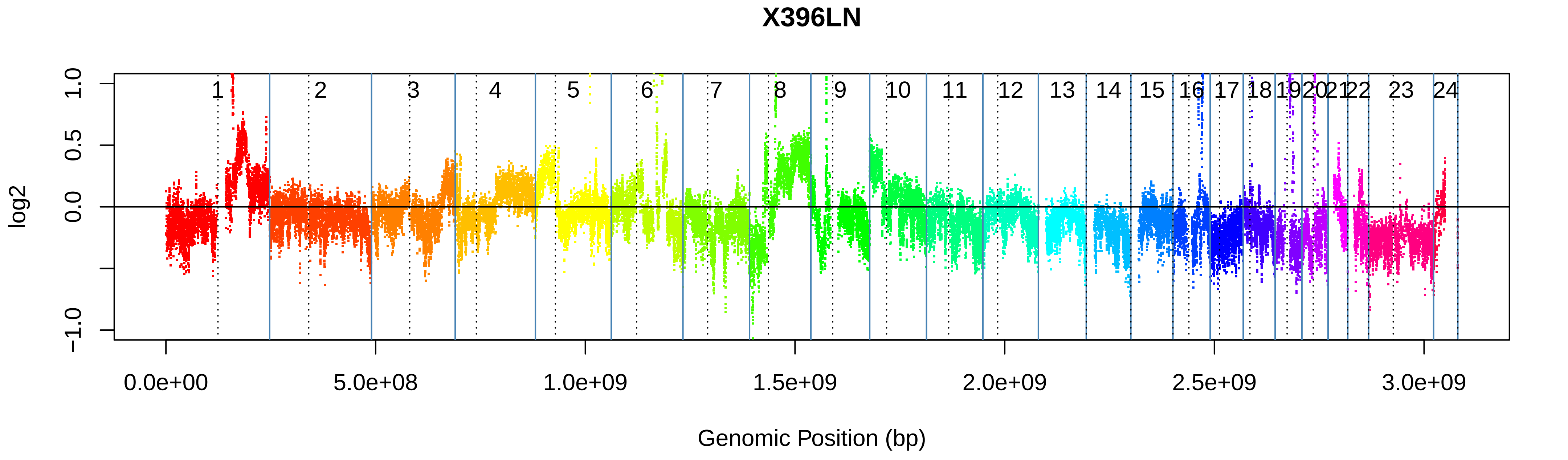
X396LN
log2
1.0
0.5
0.0
−1.0
1
2
3
4
5
6
7
8
9
10
11
12
13
14
15
16
17
18
19
20
21
22
23
24
0.0e+00
5.0e+08
1.0e+09
1.5e+09
2.0e+09
2.5e+09
3.0e+09
Genomic Position (bp)

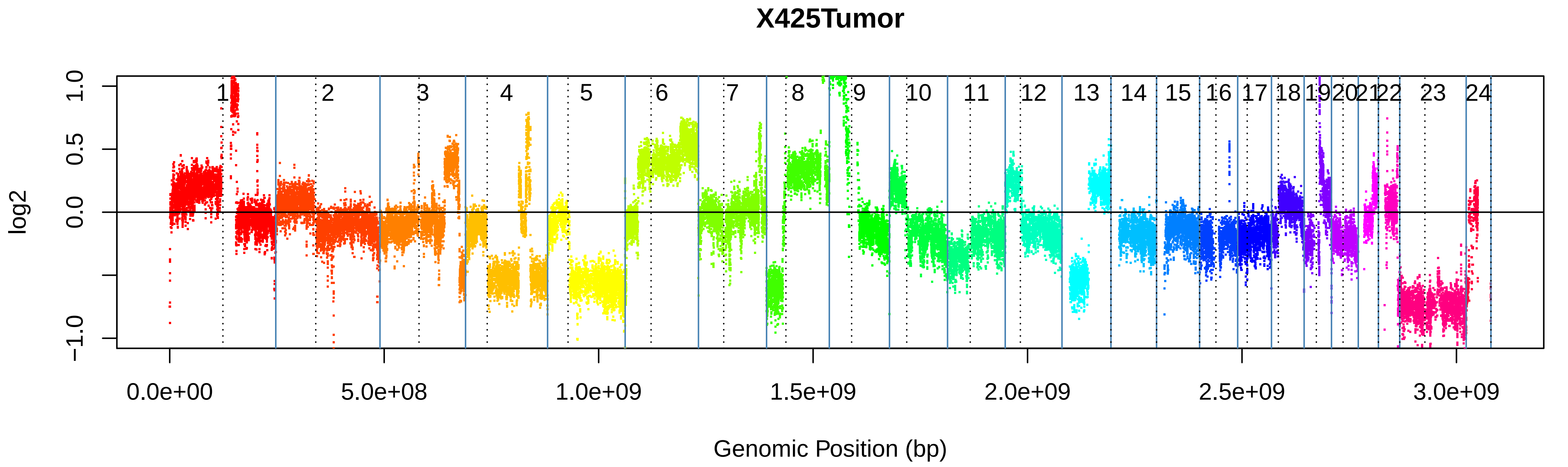
X425Tumor
log2
1.0
0.5
0.0
−1.0
1 2 3 4 5 6 7 8 9 10 11 12 13 14 15 16 17 18 19 20 21 22 23 24
0.0e+00
5.0e+08
1.0e+09
1.5e+09
2.0e+09
2.5e+09
3.0e+09
Genomic Position (bp)

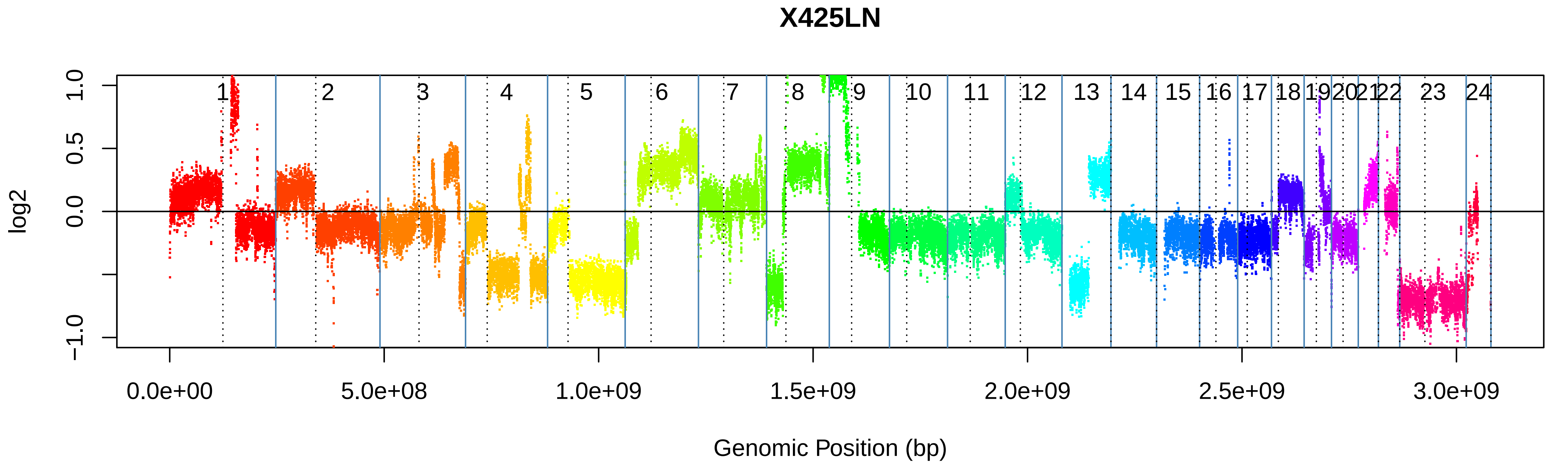
X425LN
log2
1.0
0.5
0.0
−1.0
1 2 3 4 5 6 7 8 9 10 11 12 13 14 15 16 17 18 19 20 21 22 23 24
0.0e+00
5.0e+08
1.0e+09
1.5e+09
2.0e+09
2.5e+09
3.0e+09
Genomic Position (bp)

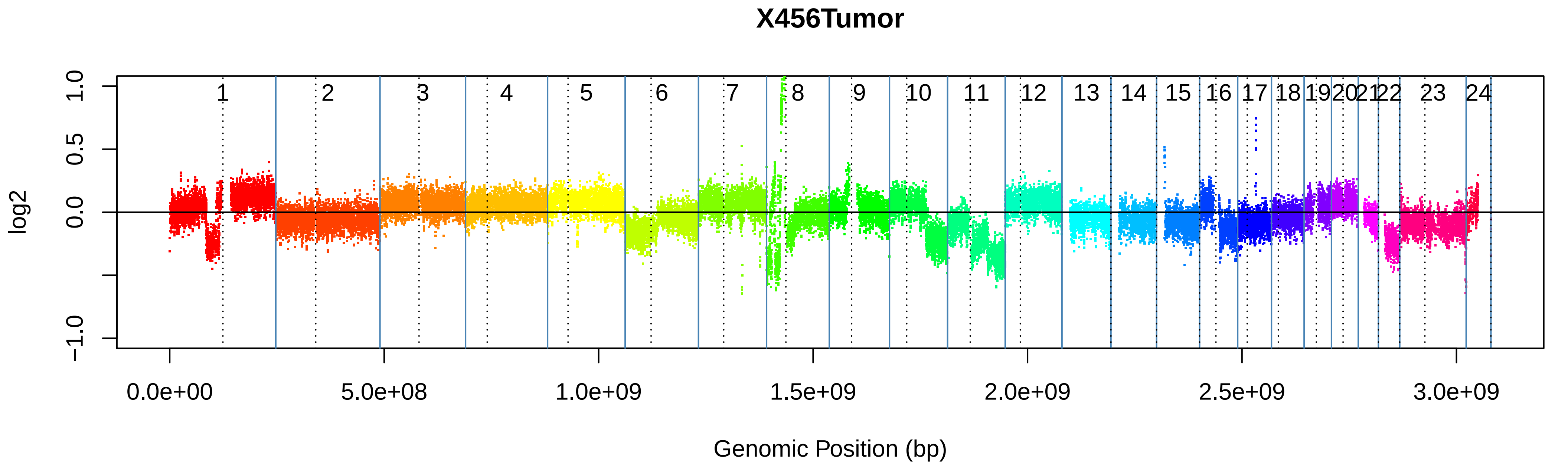
X456Tumor
log2
1.0
0.5
0.0
−1.0
1 2 3 4 5 6 7 8 9 10 11 12 13 14 15 16 17 18 19 20 21 22 23 24
0.0e+00
5.0e+08
1.0e+09
1.5e+09
2.0e+09
2.5e+09
3.0e+09
Genomic Position (bp)

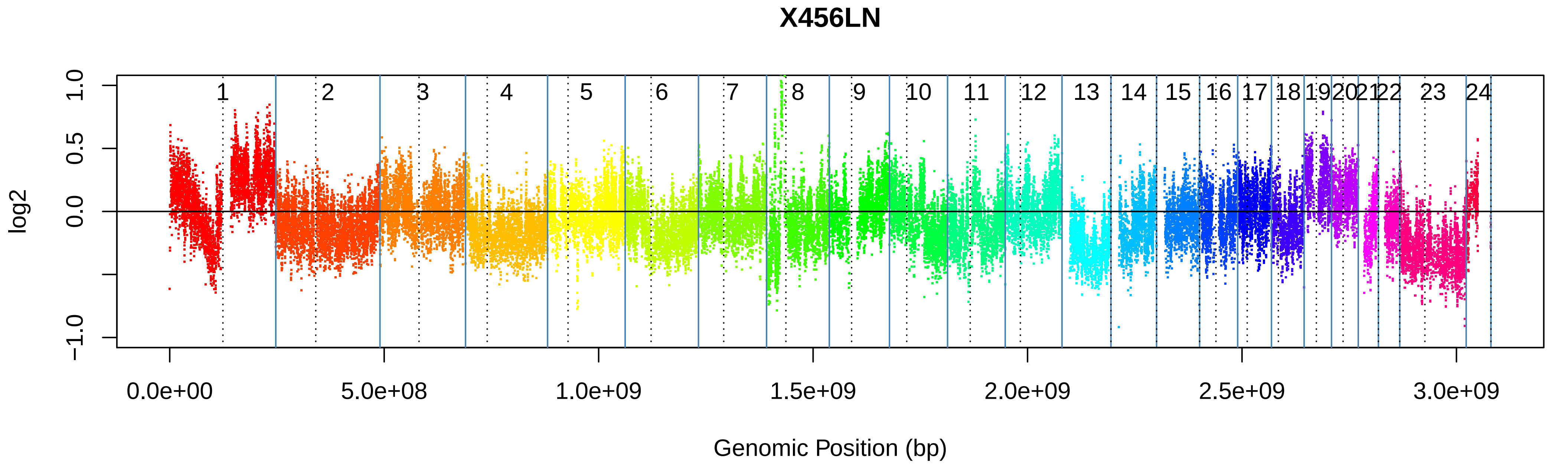
X456LN
log2
1.0
0.5
0.0
−1.0
1 2 3 4 5 6 7 8 9 10 11 12 13 14 15 16 17 18 19 20 21 22 23 24
0.0e+00
5.0e+08
1.0e+09
1.5e+09
2.0e+09
2.5e+09
3.0e+09
Genomic Position (bp)

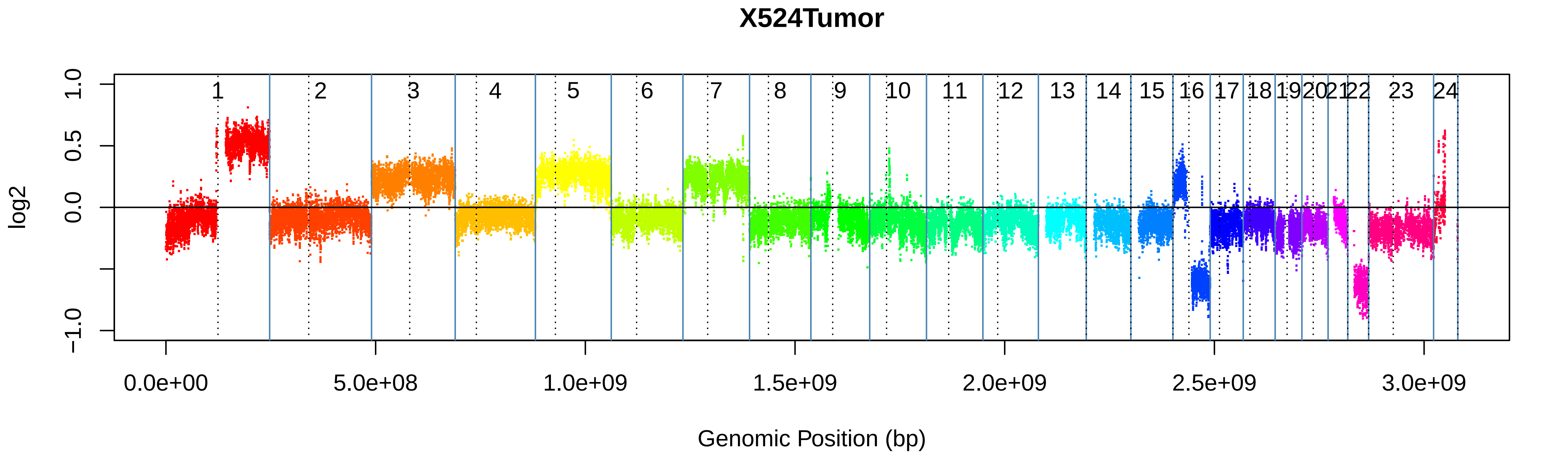
X524Tumor
log2
1.0
0.5
0.0
−1.0
1
2
3
4
5
6
7
8
9
10
11
12
13
14
15
16
17
18
19
20
21
22
23
24
0.0e+00
5.0e+08
1.0e+09
1.5e+09
2.0e+09
2.5e+09
3.0e+09
Genomic Position (bp)

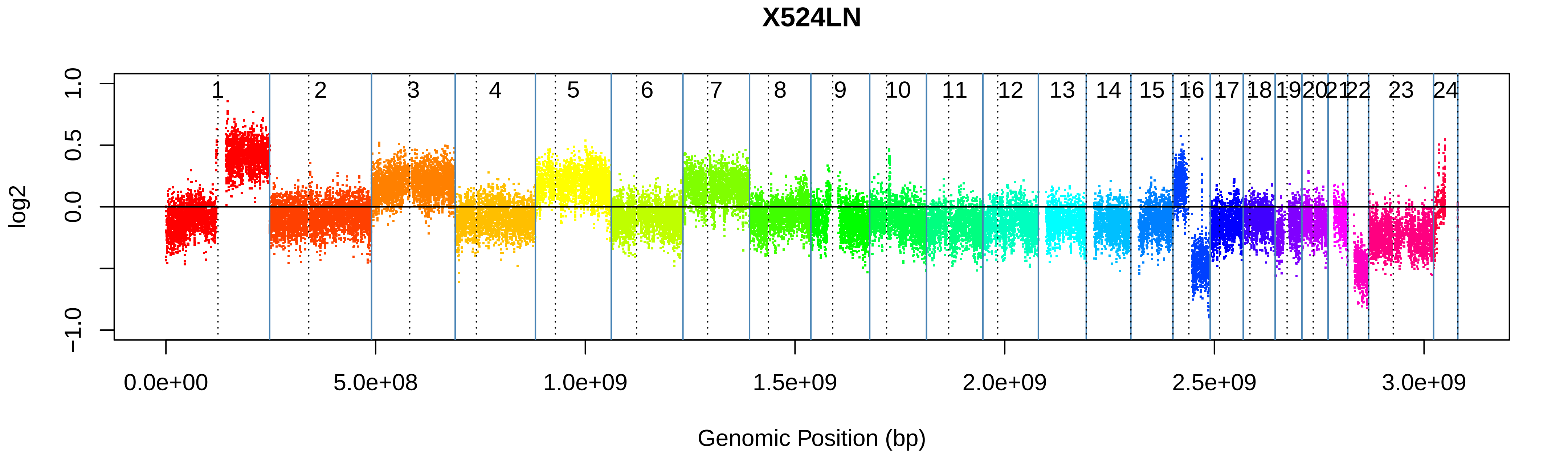
X524LN
log2
1.0
0.5
0.0
−1.0
1
2
3
4
5
6
7
8
9
10
11
12
13
14
15
16
17
18
19
20
21
22
23
24
0.0e+00
5.0e+08
1.0e+09
1.5e+09
2.0e+09
2.5e+09
3.0e+09
Genomic Position (bp)

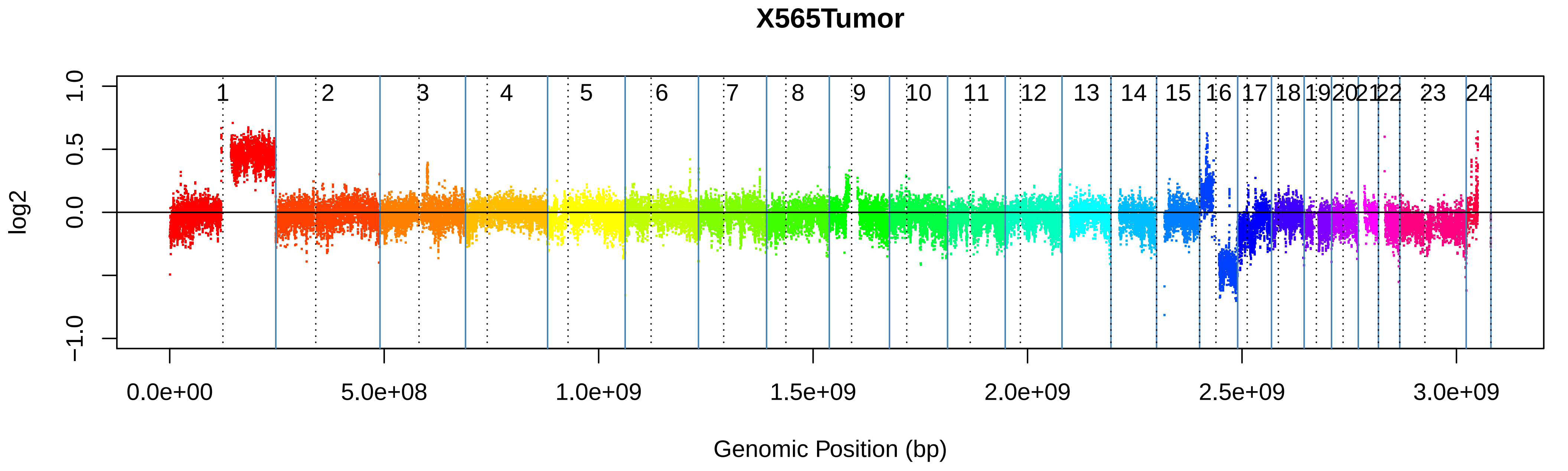
X565Tumor
log2
1.0
0.5
0.0
−1.0
1 2 3 4 5 6 7 8 9 10 11 12 13 14 15 16 17 18 19 20 21 22 23 24
0.0e+00
5.0e+08
1.0e+09
1.5e+09
2.0e+09
2.5e+09
3.0e+09
Genomic Position (bp)

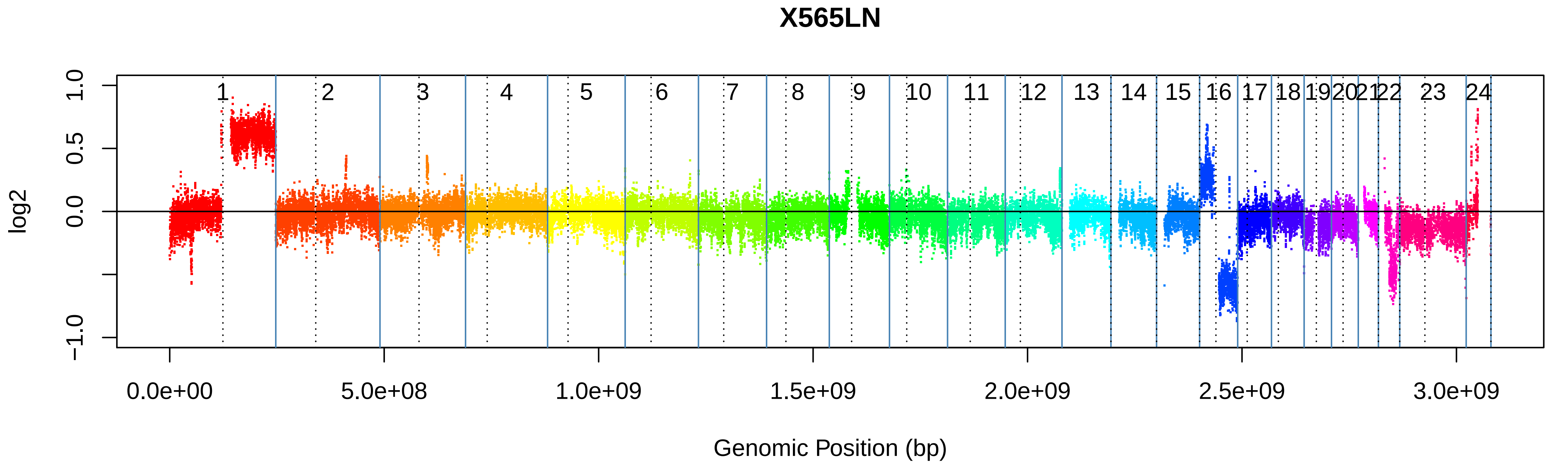
X565LN
log2
1.0
0.5
0.0
−1.0
1 2 3 4 5 6 7 8 9 10 11 12 13 14 15 16 17 18 19 20 21 22 23 24
0.0e+00
5.0e+08
1.0e+09
1.5e+09
2.0e+09
2.5e+09
3.0e+09
Genomic Position (bp)

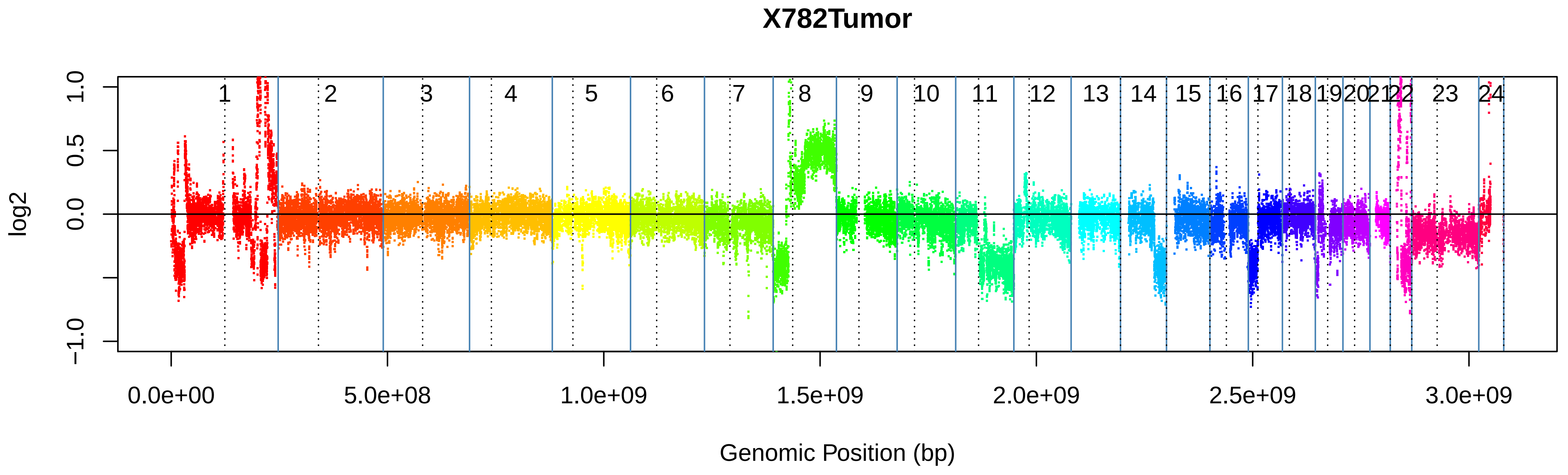
X782Tumor
log2
1.0
0.5
0.0
−1.0
1
2
3
4
5
6
7
8
9
10
11
12
13
14
15
16
17
18
19
20
21
22
23
24
0.0e+00
5.0e+08
1.0e+09
1.5e+09
2.0e+09
2.5e+09
3.0e+09
Genomic Position (bp)

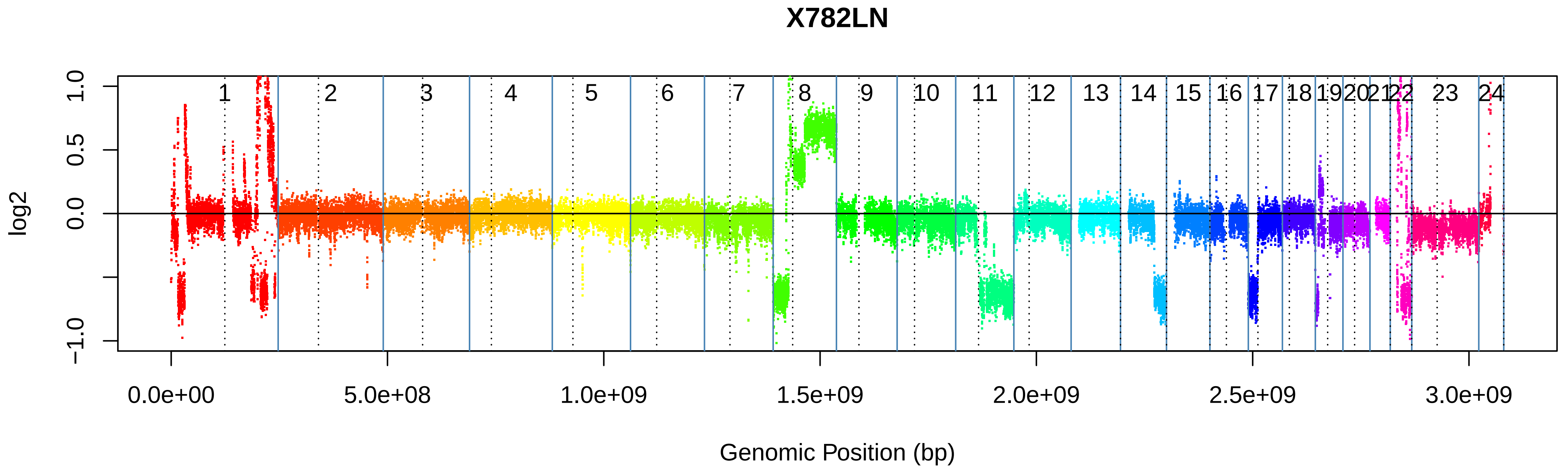
X782LN
log2
1.0
0.5
0.0
−1.0
1
2
3
4
5
6
7
8
9
10
11
12
13
14
15
16
17
18
19
20
21
22
23
24
0.0e+00
5.0e+08
1.0e+09
1.5e+09
2.0e+09
2.5e+09
3.0e+09
Genomic Position (bp)

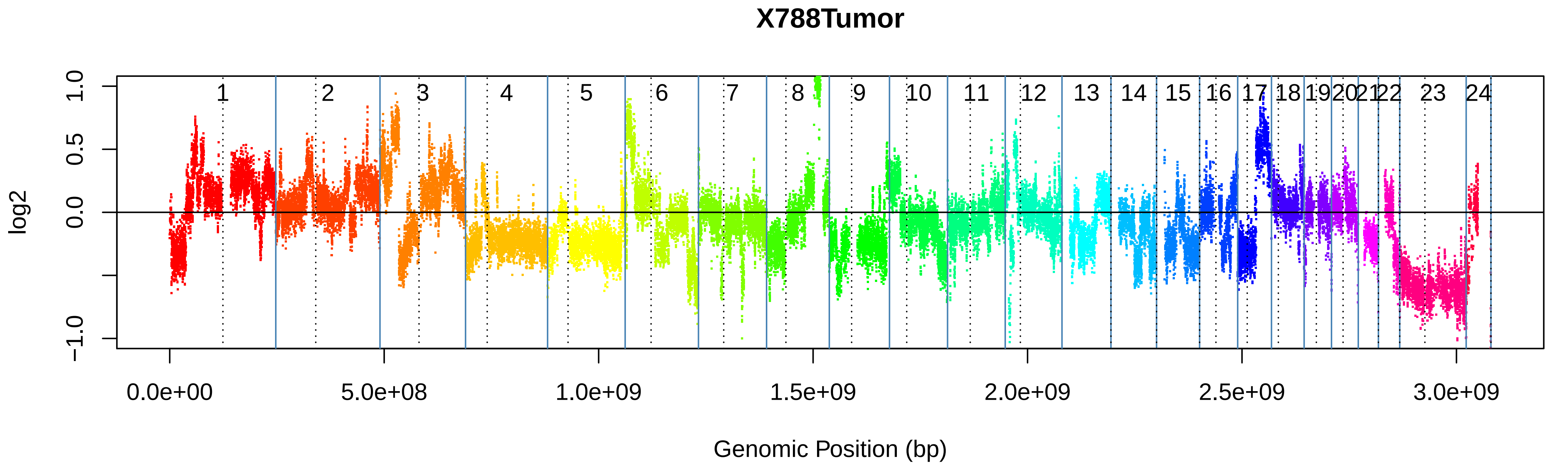
X788Tumor
log2
1.0
0.5
0.0
−1.0
1 2 3 4 5 6 7 8 9 10 11 12 13 14 15 16 17 18 19 20 21 22 23 24
0.0e+00
5.0e+08
1.0e+09
1.5e+09
2.0e+09
2.5e+09
3.0e+09
Genomic Position (bp)

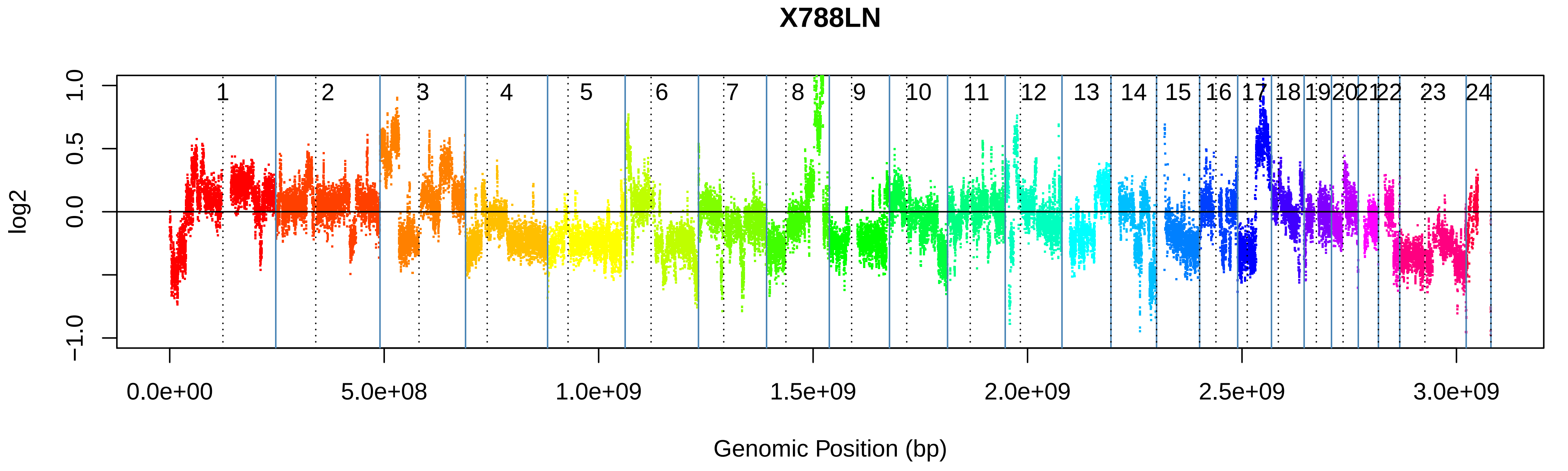
X788LN
log2
1.0
0.5
0.0
−1.0
1 2 3 4 5 6 7 8 9 10 11 12 13 14 15 16 17 18 19 20 21 22 23 24
0.0e+00
5.0e+08
1.0e+09
1.5e+09
2.0e+09
2.5e+09
3.0e+09
Genomic Position (bp)

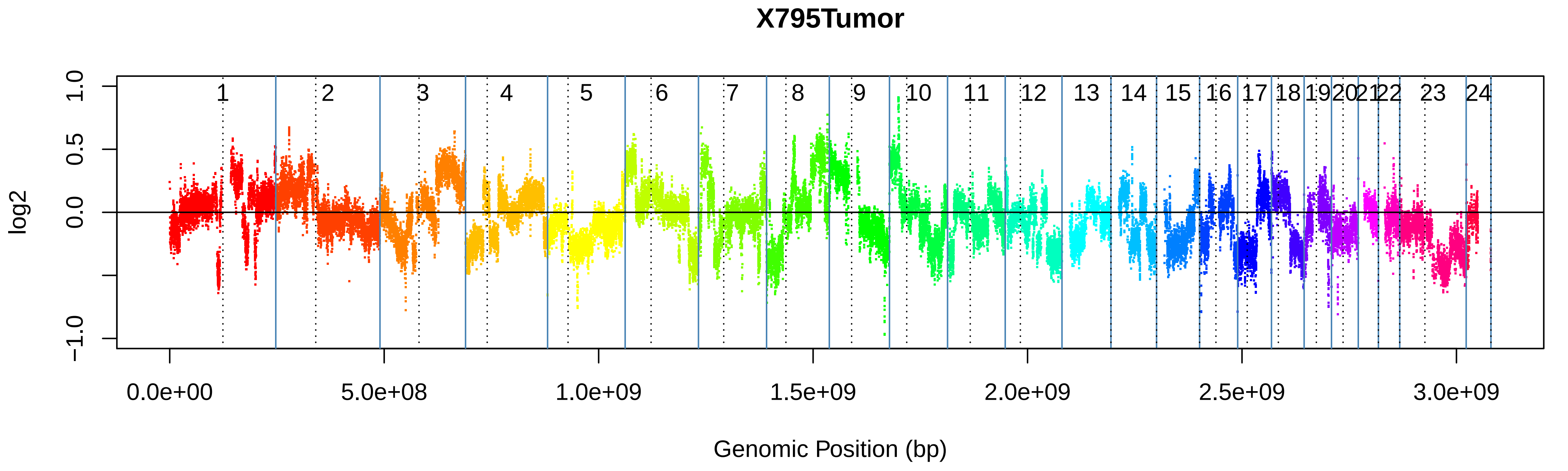

X795Tumor
1.0
0.5
0.0
−1.0
log2
1 2 3 4 5 6 7 8 9 10 11 12 13 14 15 16 17 18 19 20 21 22 23 24
0.0e+00
5.0e+08
1.0e+09
1.5e+09
2.0e+09
2.5e+09
3.0e+09
Genomic Position (bp)

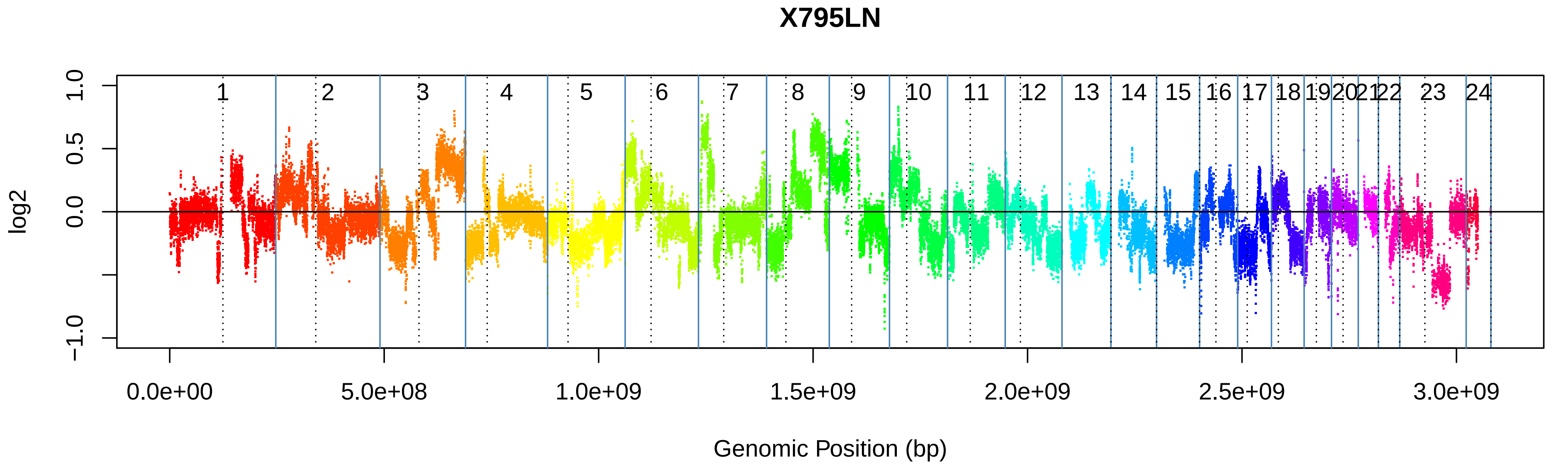

X795LN
1.0
0.5
0.0
−1.0
log2
1 2 3 4 5 6 7 8 9 10 11 12 13 14 15 16 17 18 19 20 21 22 23 24
0.0e+00
5.0e+08
1.0e+09
1.5e+09
2.0e+09
2.5e+09
3.0e+09
Genomic Position (bp)

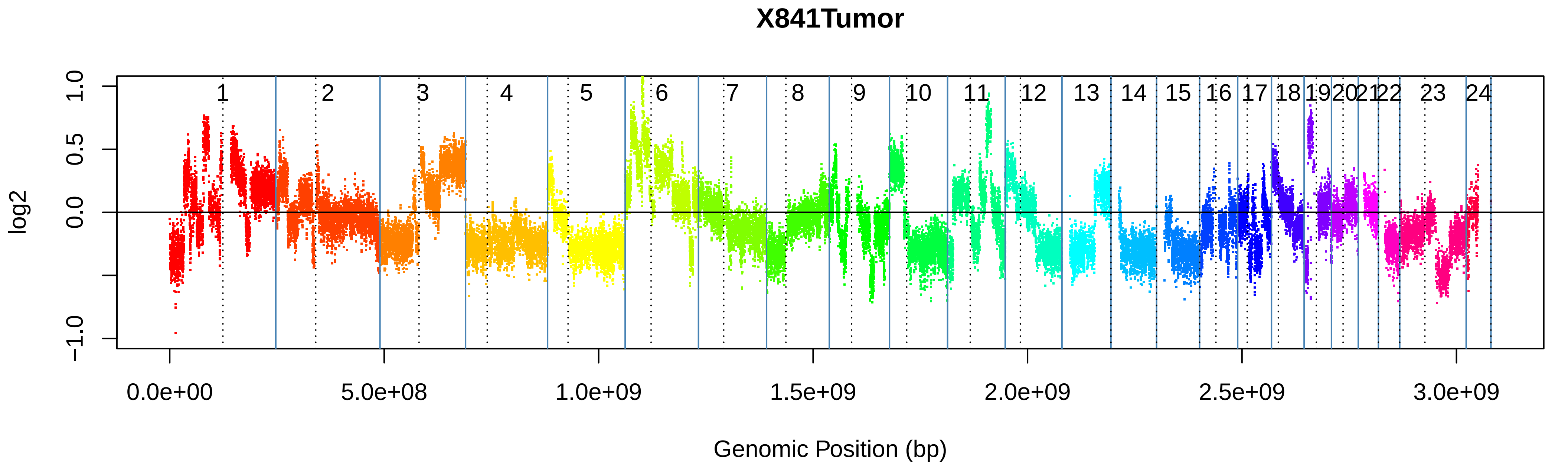
X841Tumor
log2
1.0
0.5
0.0
−1.0
1 2 3 4 5 6 7 8 9 10 11 12 13 14 15 16 17 18 19 20 21 22 23 24
0.0e+00
5.0e+08
1.0e+09
1.5e+09
2.0e+09
2.5e+09
3.0e+09
Genomic Position (bp)

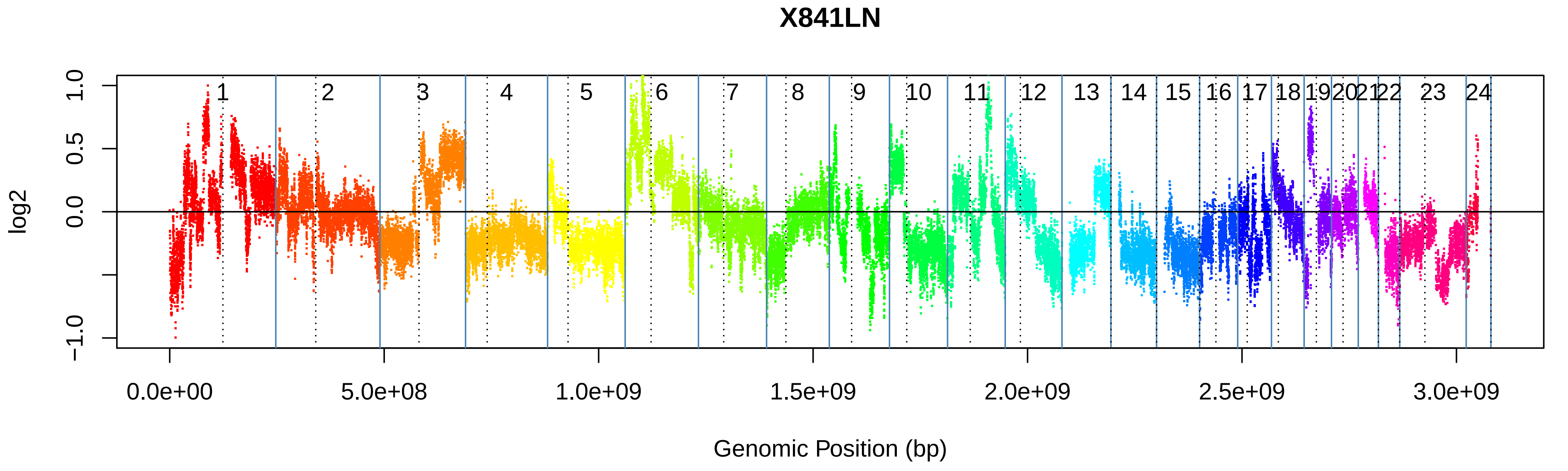
X841LN
log2
1.0
0.5
0.0
−1.0
1 2 3 4 5 6 7 8 9 10 11 12 13 14 15 16 17 18 19 20 21 22 23 24
0.0e+00
5.0e+08
1.0e+09
1.5e+09
2.0e+09
2.5e+09
3.0e+09
Genomic Position (bp)

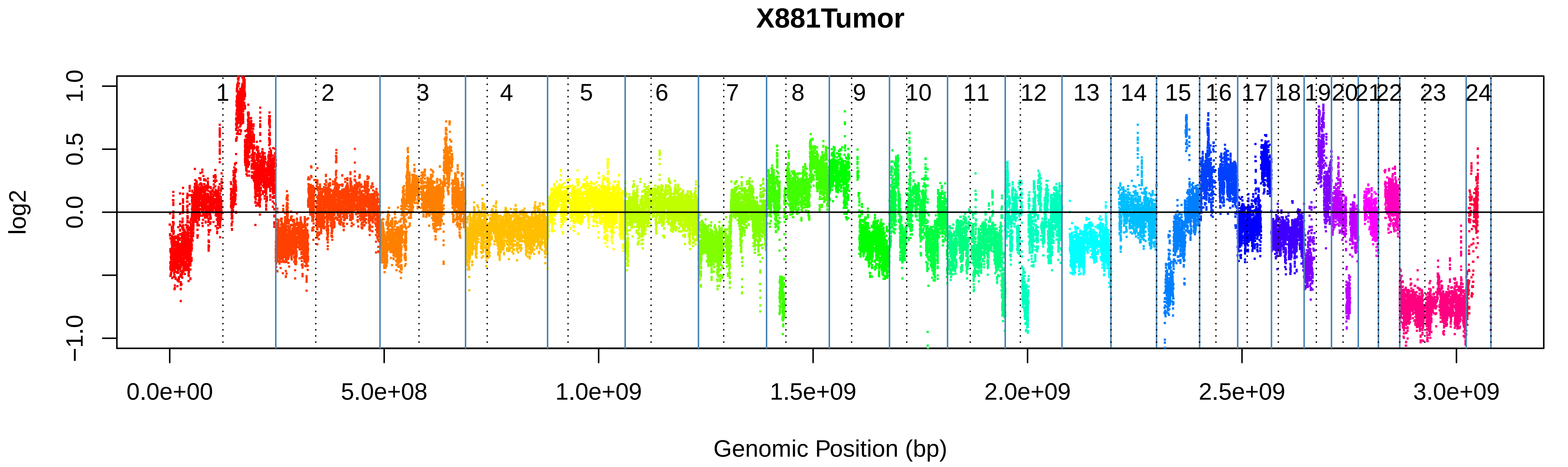
X881Tumor
log2
1.0
0.5
0.0
−1.0
1 2 3 4 5 6 7 8 9 10 11 12 13 14 15 16 17 18 19 20 21 22 23 24
0.0e+00
5.0e+08
1.0e+09
1.5e+09
2.0e+09
2.5e+09
3.0e+09
Genomic Position (bp)

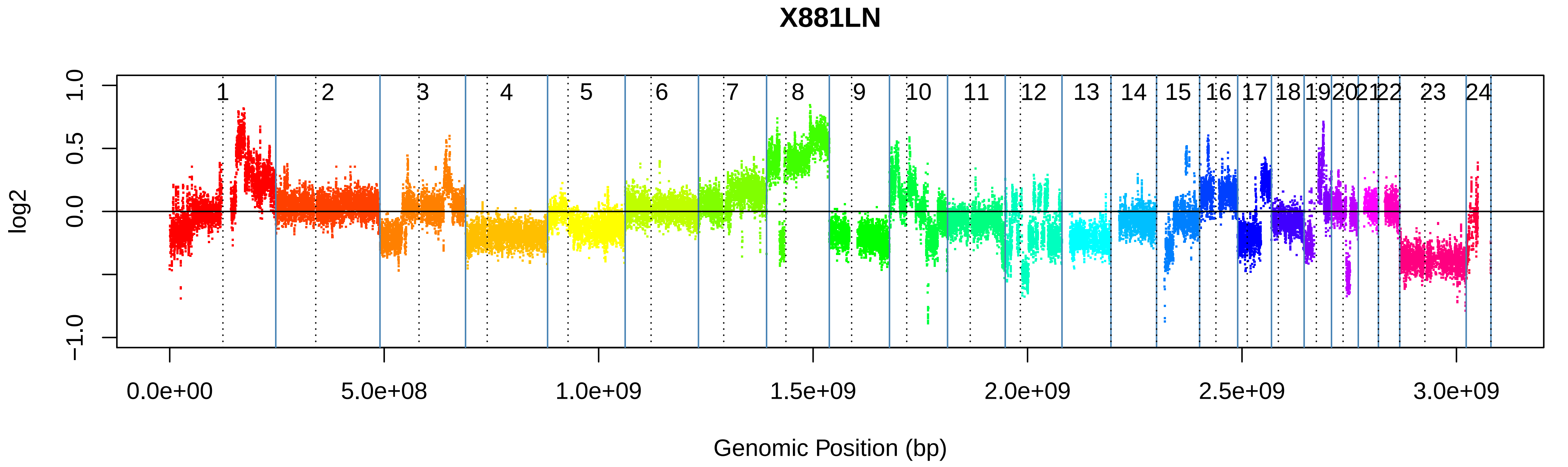
X881LN
log2
1.0
0.5
0.0
−1.0
1 2 3 4 5 6 7 8 9 10 11 12 13 14 15 16 17 18 19 20 21 22 23 24
0.0e+00
5.0e+08
1.0e+09
1.5e+09
2.0e+09
2.5e+09
3.0e+09
Genomic Position (bp)

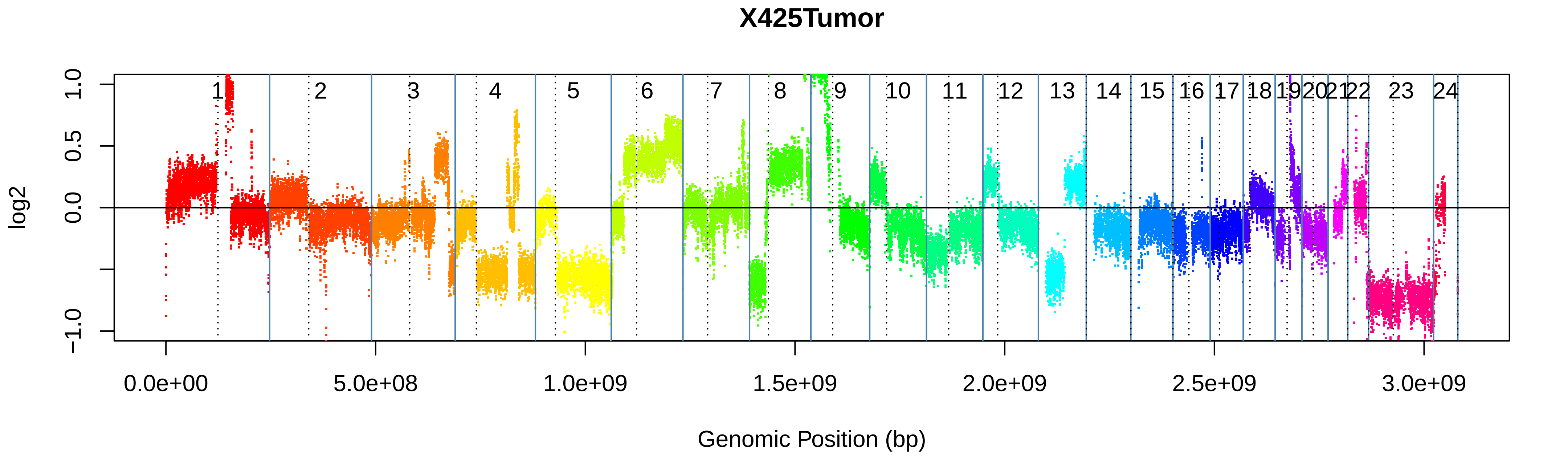
X425Tumor
log2
1.0
0.5
0.0
−1.0
1 2 3 4 5 6 7 8 9 10 11 12 13 14 15 16 17 18 19 20 21 22 23 24
0.0e+00
5.0e+08
1.0e+09
1.5e+09
2.0e+09
2.5e+09
3.0e+09
Genomic Position (bp)

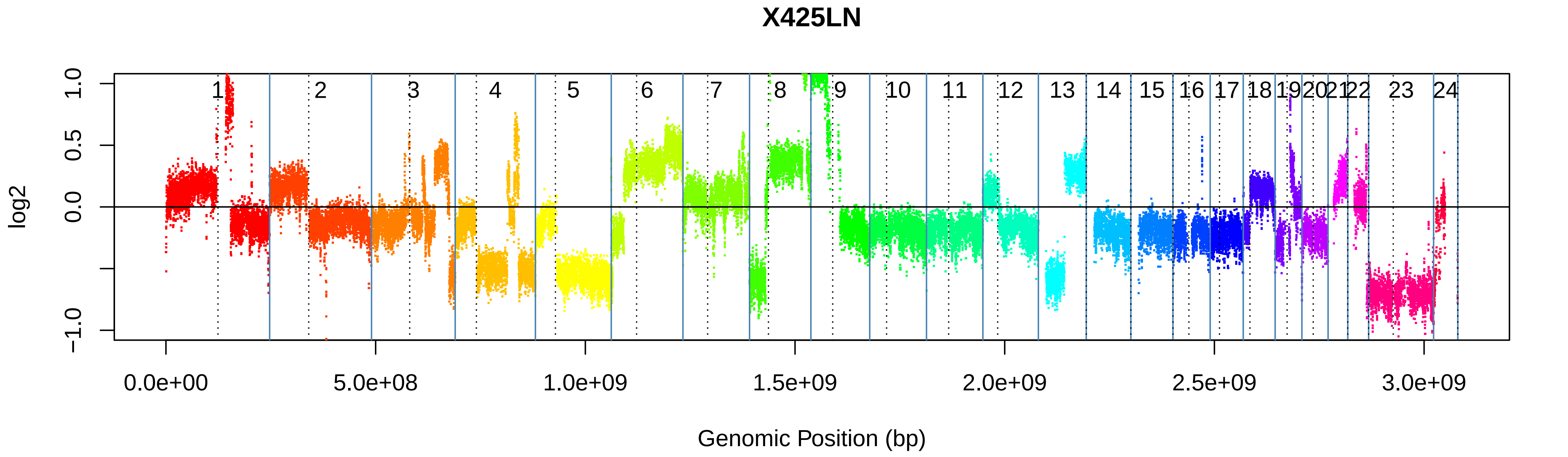
X425LN
log2
1.0
0.5
0.0
−1.0
1 2 3 4 5 6 7 8 9 10 11 12 13 14 15 16 17 18 19 20 21 22 23 24
0.0e+00
5.0e+08
1.0e+09
1.5e+09
2.0e+09
2.5e+09
3.0e+09
Genomic Position (bp)

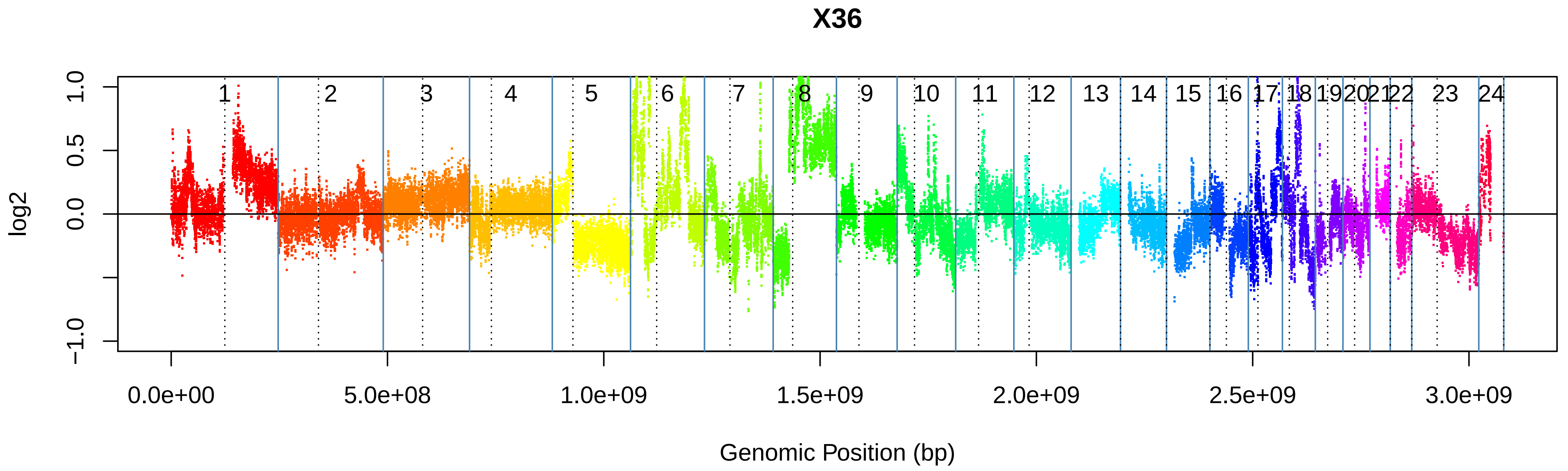
X36
log2
1.0
0.5
0.0
−1.0
1 2 3 4 5 6 7 8 9 10 11 12 13 14 15 16 17 18 19 20 21 22 23 24
0.0e+00
5.0e+08
1.0e+09
1.5e+09
2.0e+09
2.5e+09
3.0e+09
Genomic Position (bp)

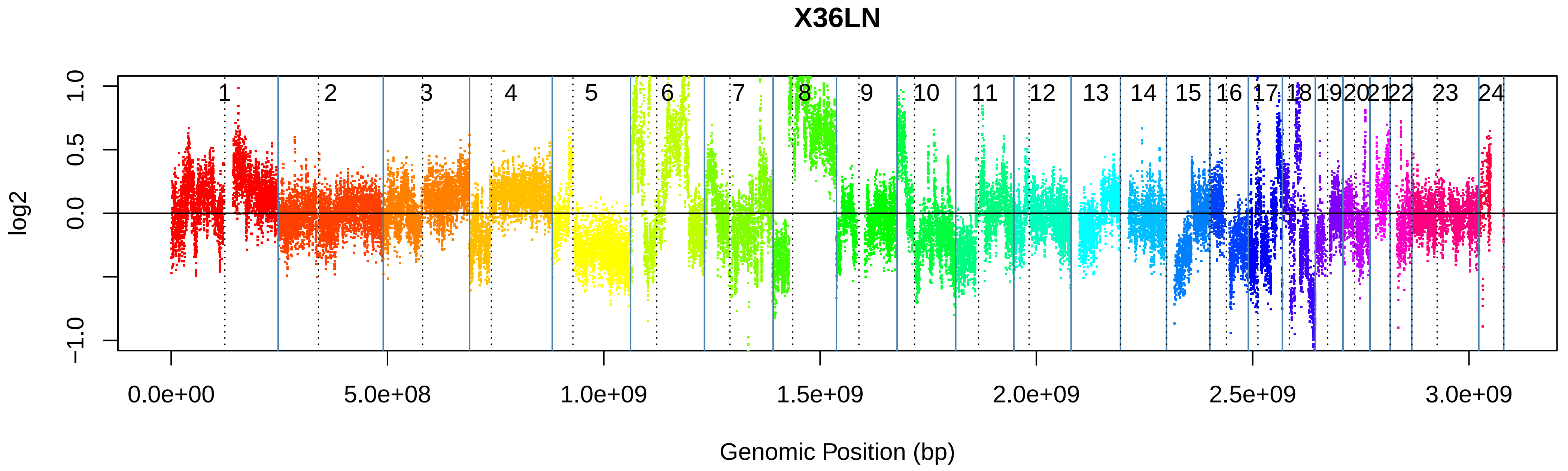
X36LN
log2
1.0
0.5
0.0
−1.0
1 2 3 4 5 6 7 8 9 10 11 12 13 14 15 16 17 18 19 20 21 22 23 24
0.0e+00
5.0e+08
1.0e+09
1.5e+09
2.0e+09
2.5e+09
3.0e+09
Genomic Position (bp)

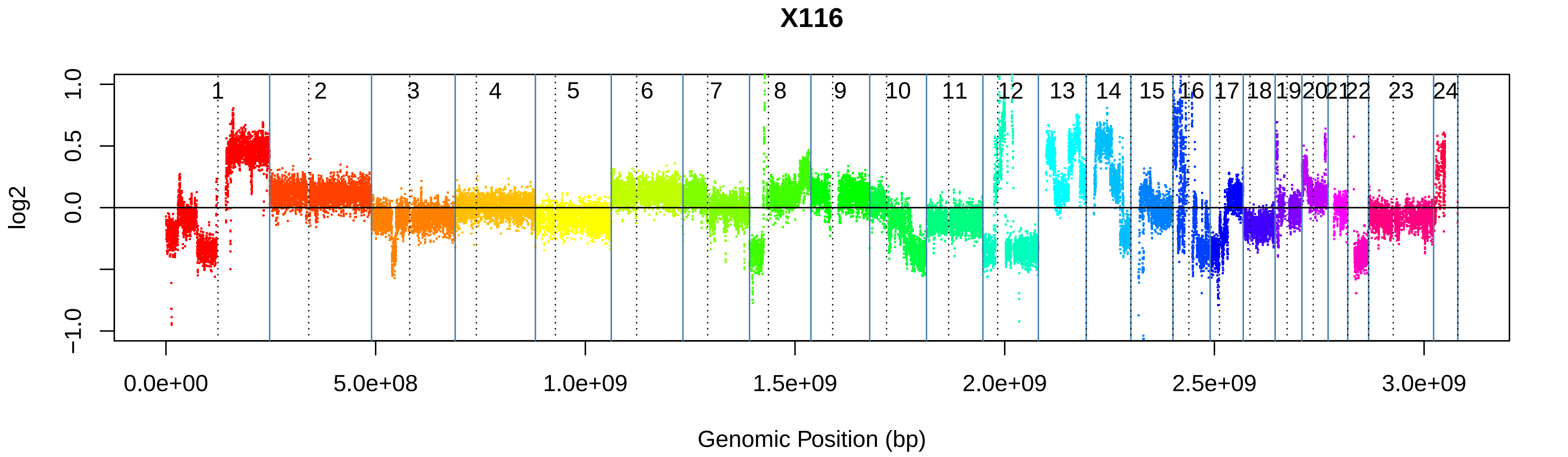
X116
log2
1.0
0.5
0.0
−1.0
1
2
3
4
5
6
7
8
9
10
11
12
13
14
15
16
17
18
19
20
21
22
23
24
0.0e+00
5.0e+08
1.0e+09
1.5e+09
2.0e+09
2.5e+09
3.0e+09
Genomic Position (bp)

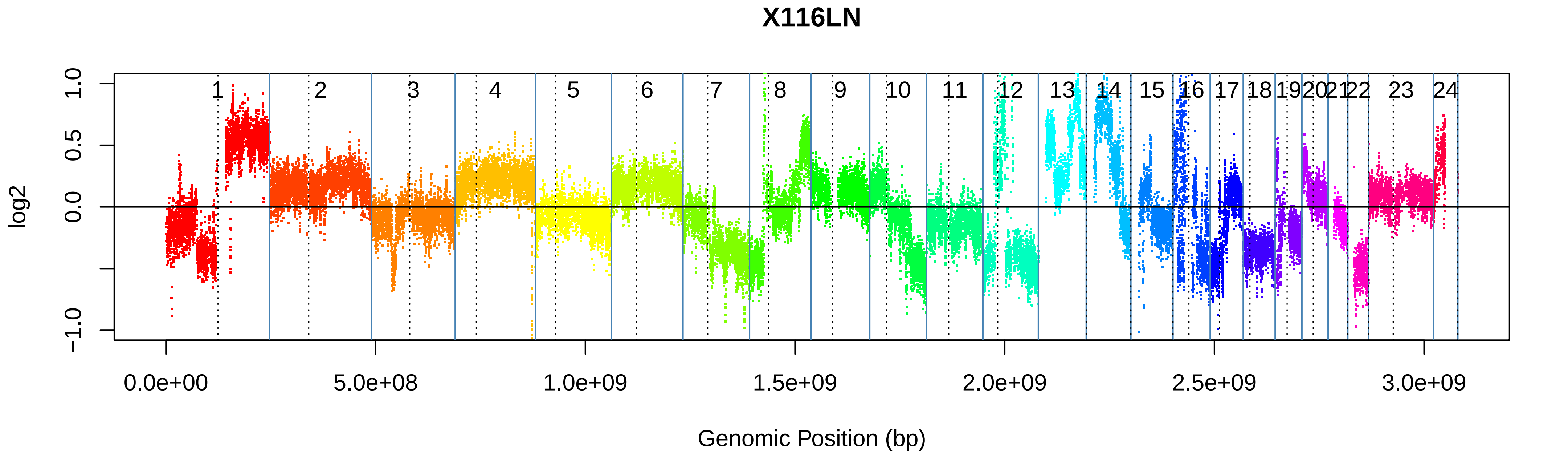
X116LN
log2
1.0
0.5
0.0
−1.0
1
2
3
4
5
6
7
8
9
10
11
12
13
14
15
16
17
18
19
20
21
22
23
24
0.0e+00
5.0e+08
1.0e+09
1.5e+09
2.0e+09
2.5e+09
3.0e+09
Genomic Position (bp)

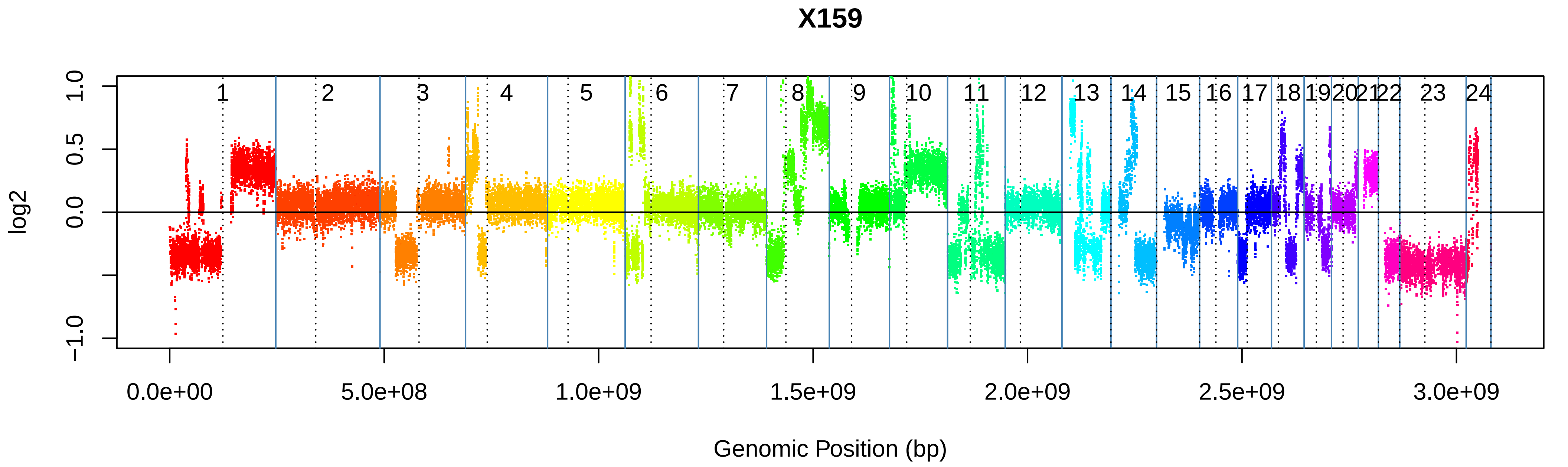
X159
log2
1.0
0.5
0.0
−1.0
1
2
3
4
5
6
7
8
9
10
11
12
13
14
15
16
17
18
19
20
21
22
23
24
0.0e+00
5.0e+08
1.0e+09
1.5e+09
2.0e+09
2.5e+09
3.0e+09
Genomic Position (bp)

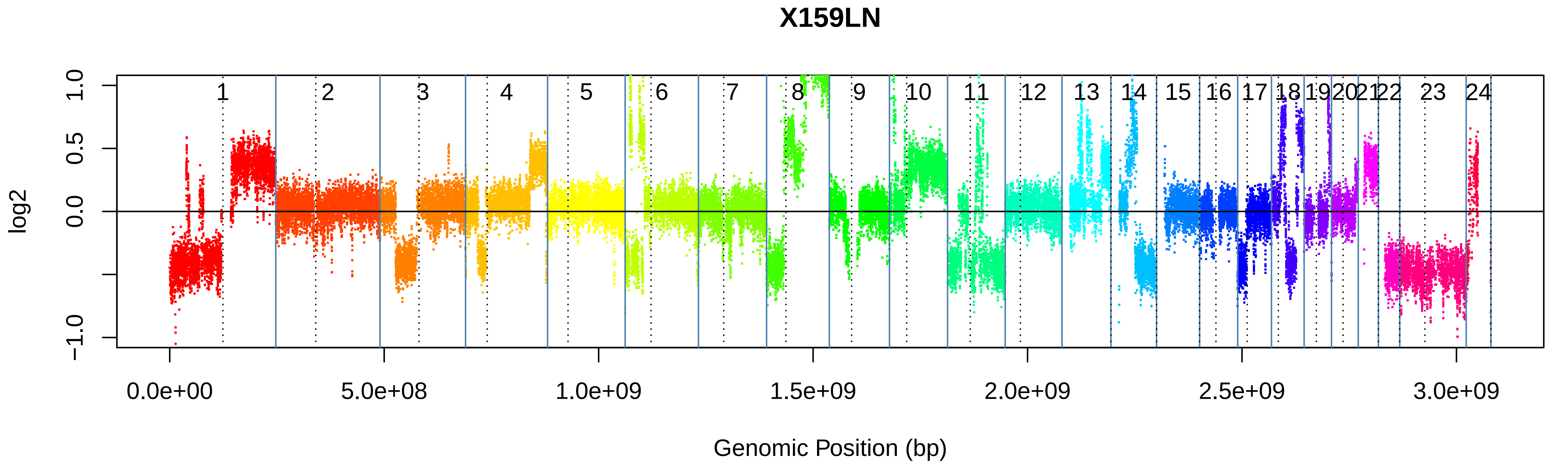
X159LN
log2
1.0
0.5
0.0
−1.0
1
2
3
4
5
6
7
8
9
10
11
12
13
14
15
16
17
18
19
20
21
22
23
24
0.0e+00
5.0e+08
1.0e+09
1.5e+09
2.0e+09
2.5e+09
3.0e+09
Genomic Position (bp)

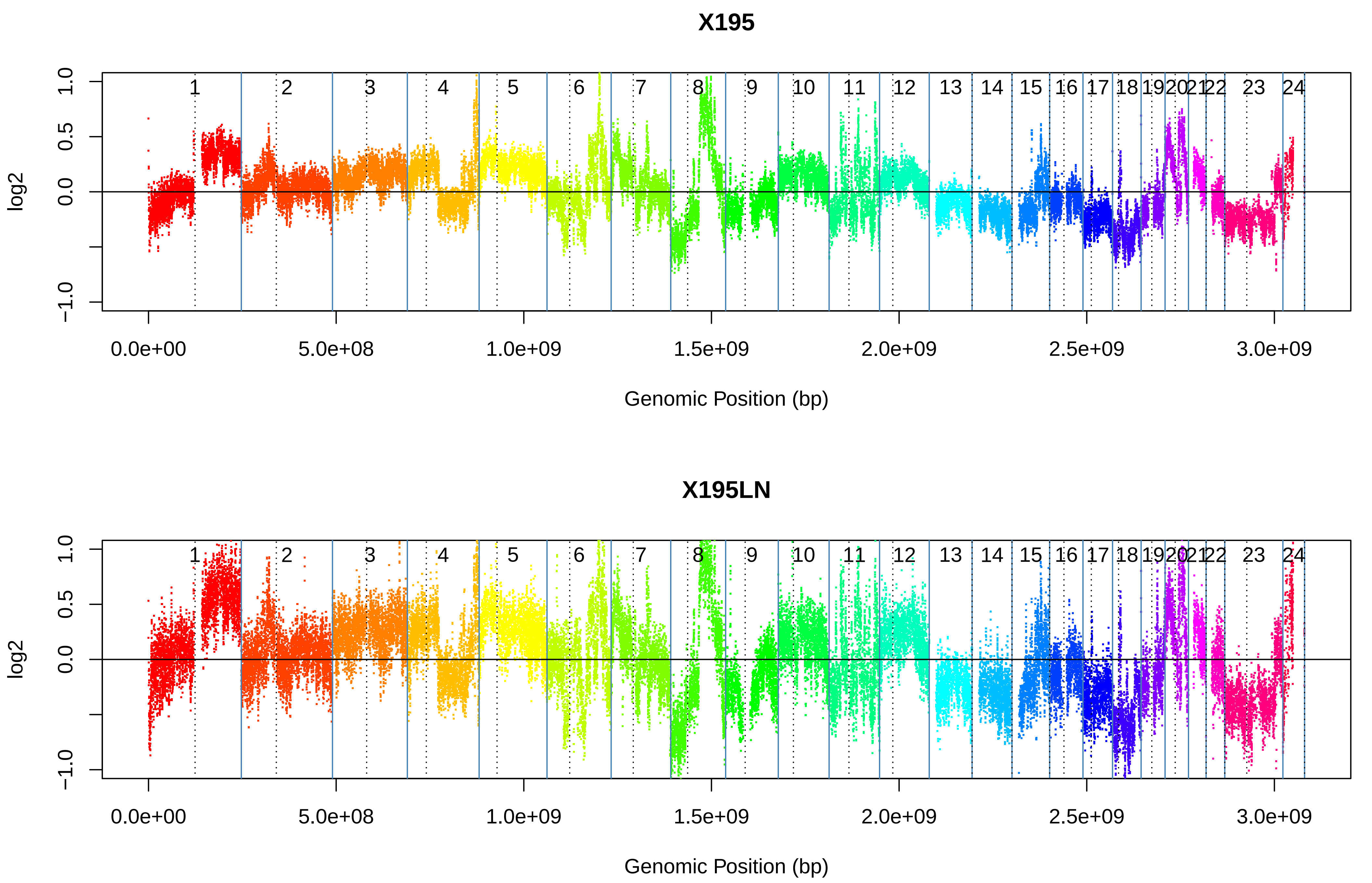
X195
log2
1.0
0.5
0.0
−1.0
1
2
3
4
5
6
7
8
9
10
11
12
13
14
15
16
17
18
19
20
21
22
23
24
0.0e+00
5.0e+08
1.0e+09
1.5e+09
2.0e+09
2.5e+09
3.0e+09
Genomic Position (bp)
X195LN
Genomic Position (bp)

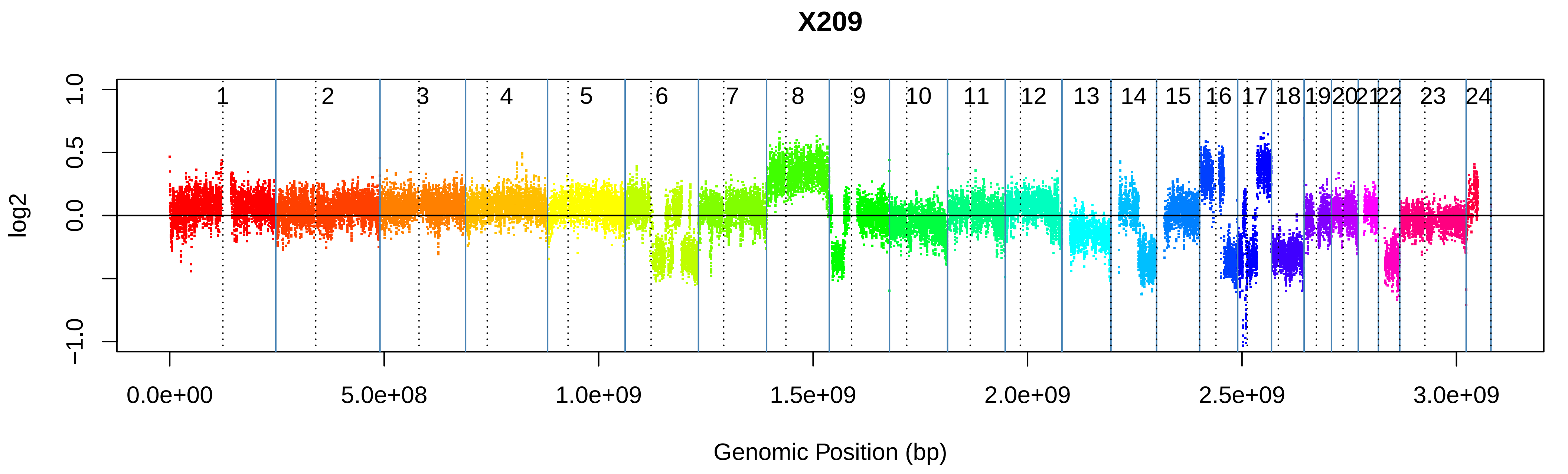

X209
1 2 3 4 5 6 7 8 9 10 11 12 13 14 15 16 17 18 19 20 21 22 23 24
log2
1.0
0.5
0.0
−1.0
0.0e+00
5.0e+08
1.0e+09
1.5e+09
2.0e+09
2.5e+09
3.0e+09
Genomic Position (bp)

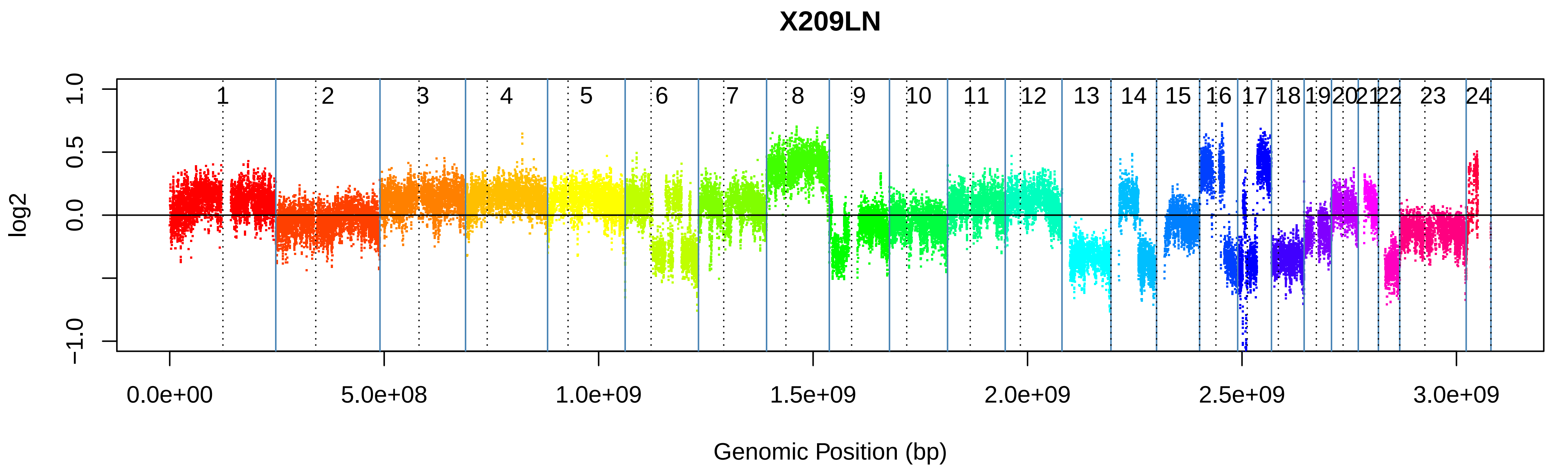

X209LN
1 2 3 4 5 6 7 8 9 10 11 12 13 14 15 16 17 18 19 20 21 22 23 24
log2
1.0
0.5
0.0
−1.0
0.0e+00
5.0e+08
1.0e+09
1.5e+09
2.0e+09
2.5e+09
3.0e+09
Genomic Position (bp)

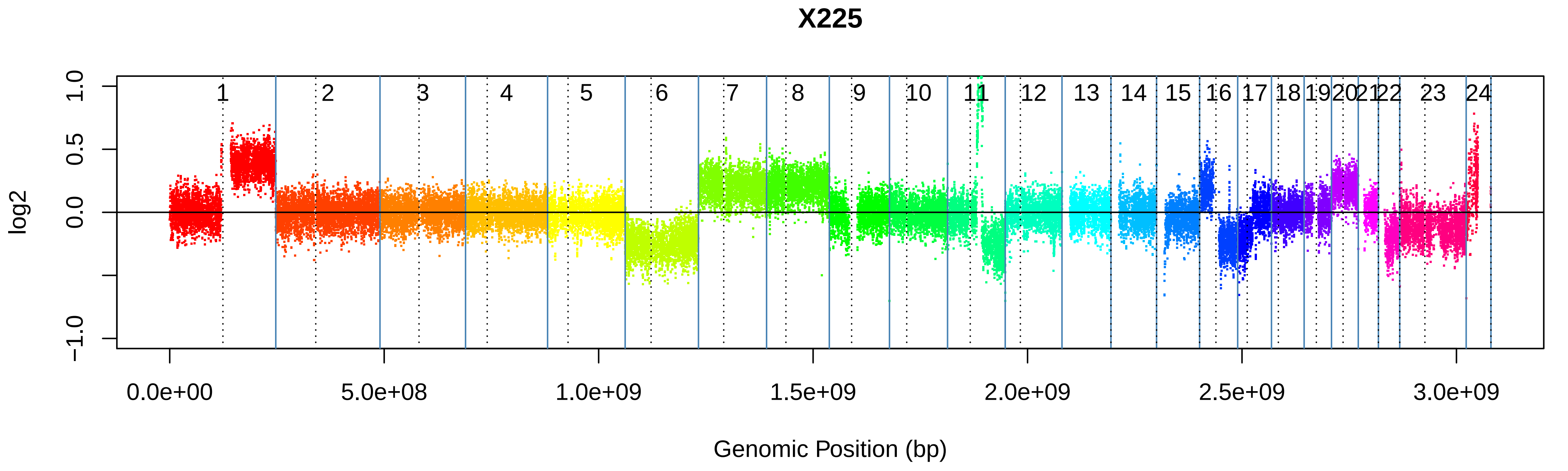

X225
1.0
0.5
0.0
−1.0
log2
1
2
3
4
5
6
7
8
9
10
11
12
13
14
15
16
17
18
19
20
21
22
23
24
0.0e+00
5.0e+08
1.0e+09
1.5e+09
2.0e+09
2.5e+09
3.0e+09
Genomic Position (bp)

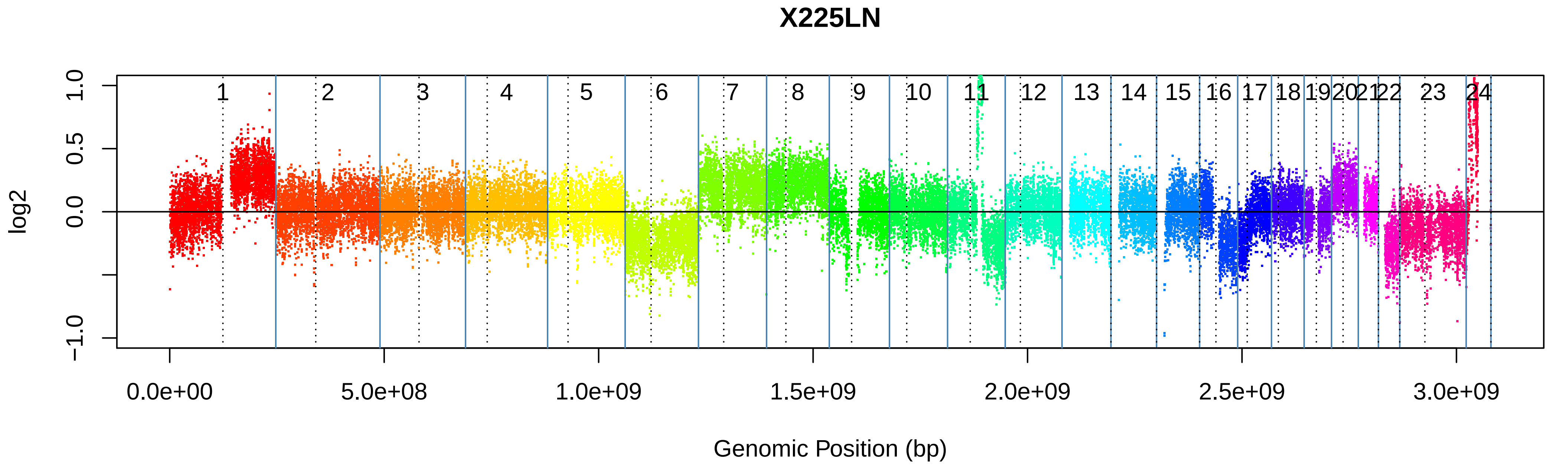

X225LN
1.0
0.5
0.0
−1.0
log2
1
2
3
4
5
6
7
8
9
10
11
12
13
14
15
16
17
18
19
20
21
22
23
24
0.0e+00
5.0e+08
1.0e+09
1.5e+09
2.0e+09
2.5e+09
3.0e+09
Genomic Position (bp)

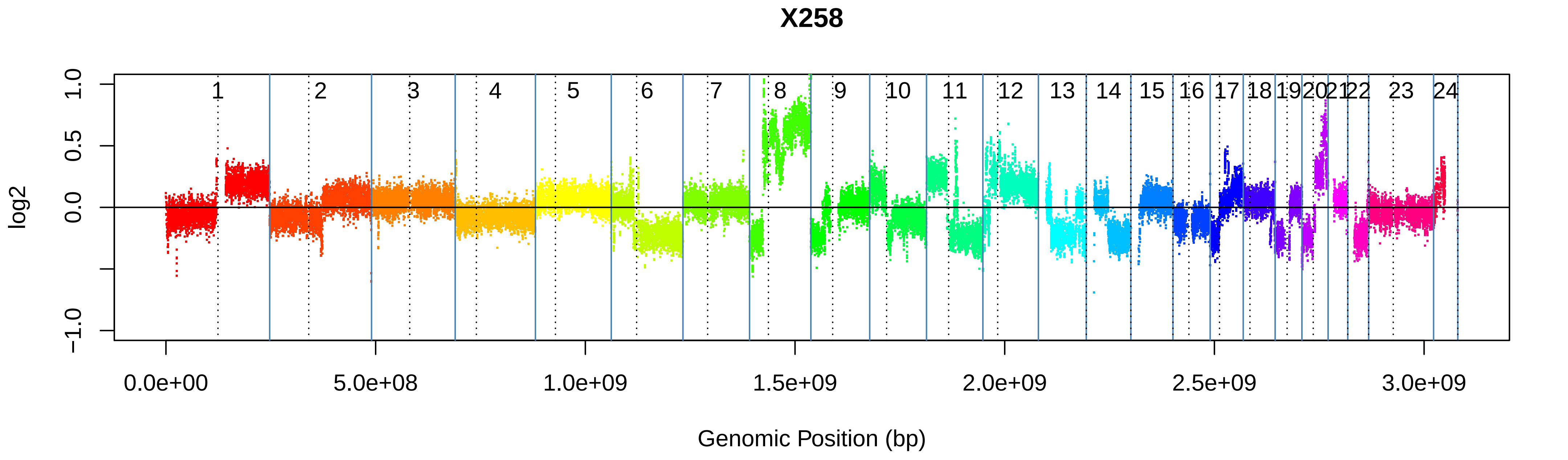
X258
log2
1.0
0.5
0.0
−1.0
1 2 3 4 5 6 7 8 9 10 11 12 13 14 15 16 17 18 19 20 21 22 23 24
0.0e+00
5.0e+08
1.0e+09
1.5e+09
2.0e+09
2.5e+09
3.0e+09
Genomic Position (bp)

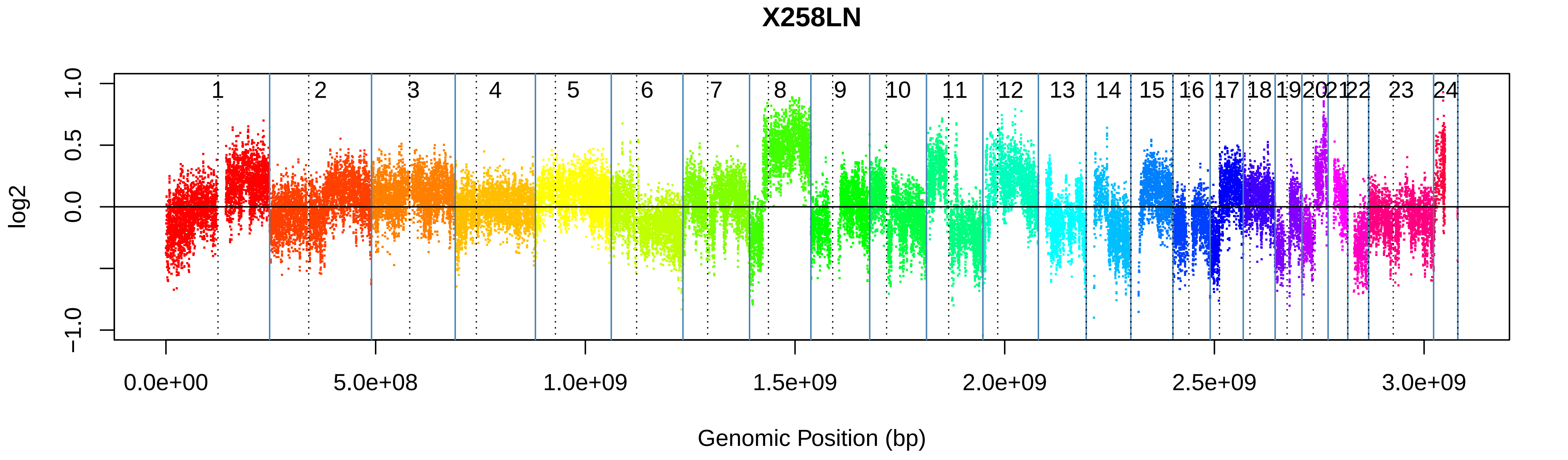
X258LN
log2
1.0
0.5
0.0
−1.0
1 2 3 4 5 6 7 8 9 10 11 12 13 14 15 16 17 18 19 20 21 22 23 24
0.0e+00
5.0e+08
1.0e+09
1.5e+09
2.0e+09
2.5e+09
3.0e+09
Genomic Position (bp)

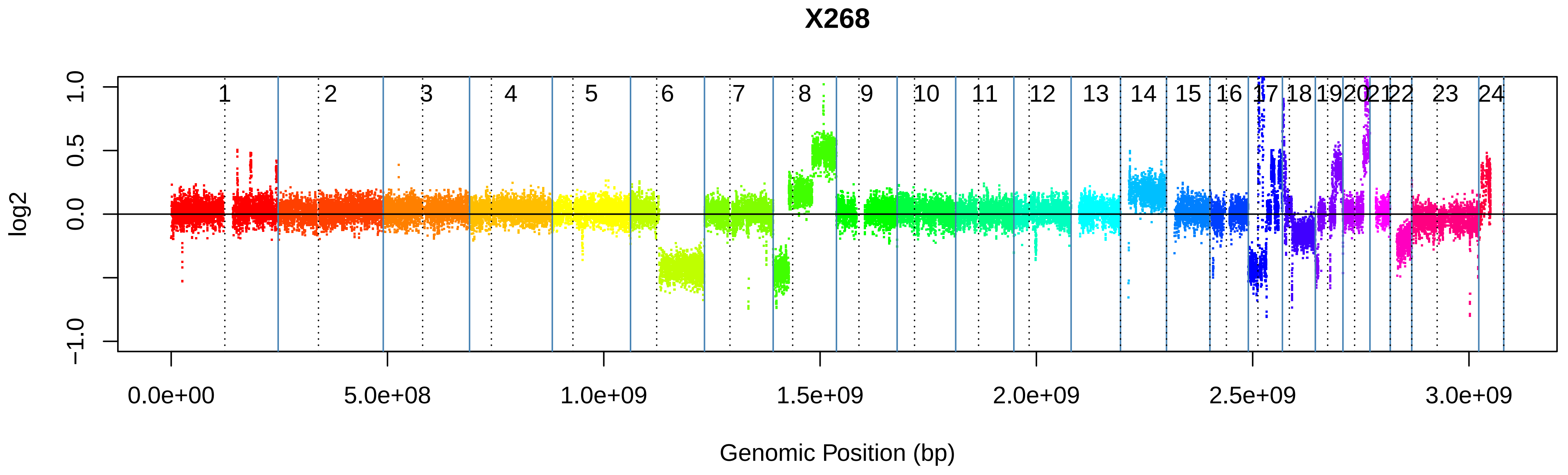
X268
log2
1.0
0.5
0.0
−1.0
1
2
3
4
5
6
7
8
9
10
11
12
13
14
15
16
17
18
19
20
21
22
23
24
0.0e+00
5.0e+08
1.0e+09
1.5e+09
2.0e+09
2.5e+09
3.0e+09
Genomic Position (bp)

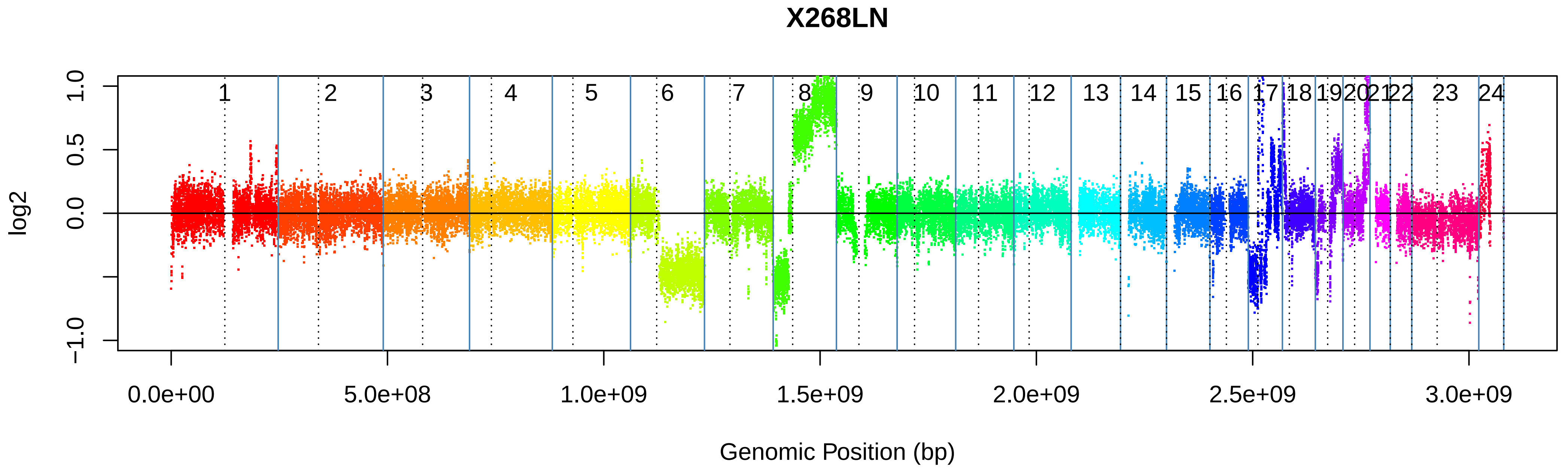
X268LN
log2
1.0
0.5
0.0
−1.0
1
2
3
4
5
6
7
8
9
10
11
12
13
14
15
16
17
18
19
20
21
22
23
24
0.0e+00
5.0e+08
1.0e+09
1.5e+09
2.0e+09
2.5e+09
3.0e+09
Genomic Position (bp)

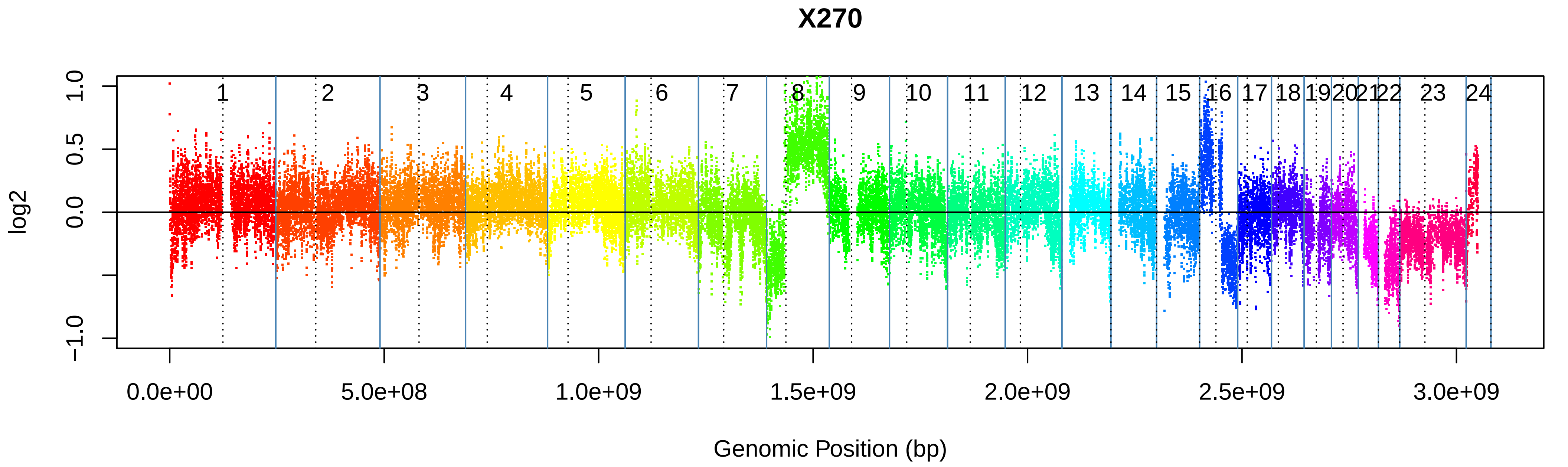
X270
log2
1.0
0.5
0.0
−1.0
1 2 3 4 5 6 7 8 9 10 11 12 13 14 15 16 17 18 19 20 21 22 23 24
0.0e+00
5.0e+08
1.0e+09
1.5e+09
2.0e+09
2.5e+09
3.0e+09
Genomic Position (bp)

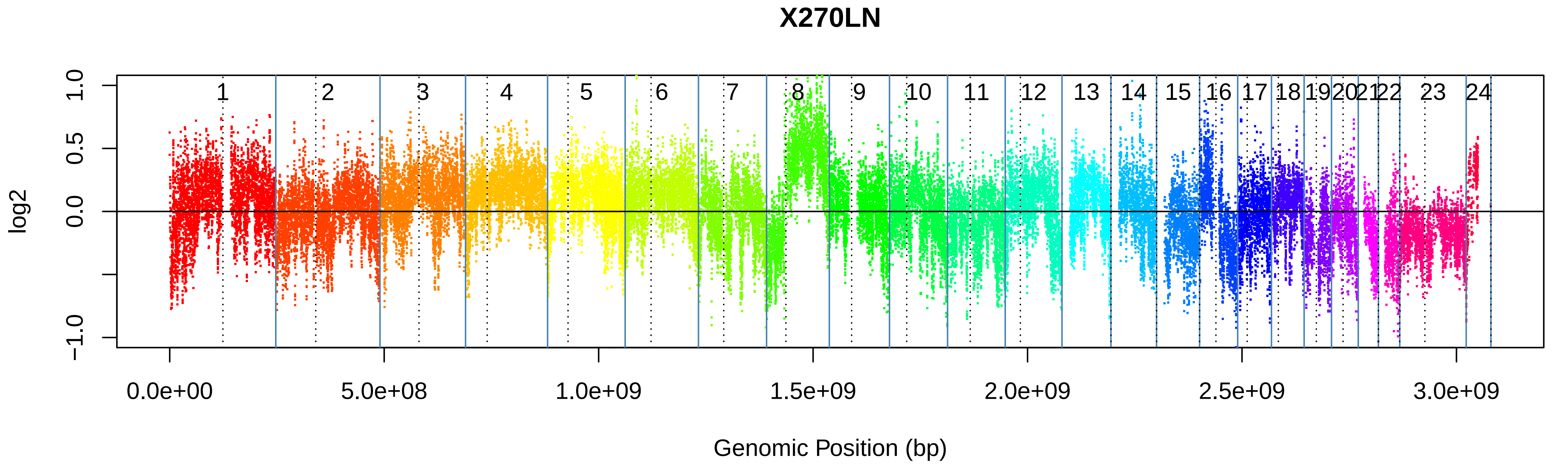
X270LN
log2
1.0
0.5
0.0
−1.0
1 2 3 4 5 6 7 8 9 10 11 12 13 14 15 16 17 18 19 20 21 22 23 24
0.0e+00
5.0e+08
1.0e+09
1.5e+09
2.0e+09
2.5e+09
3.0e+09
Genomic Position (bp)

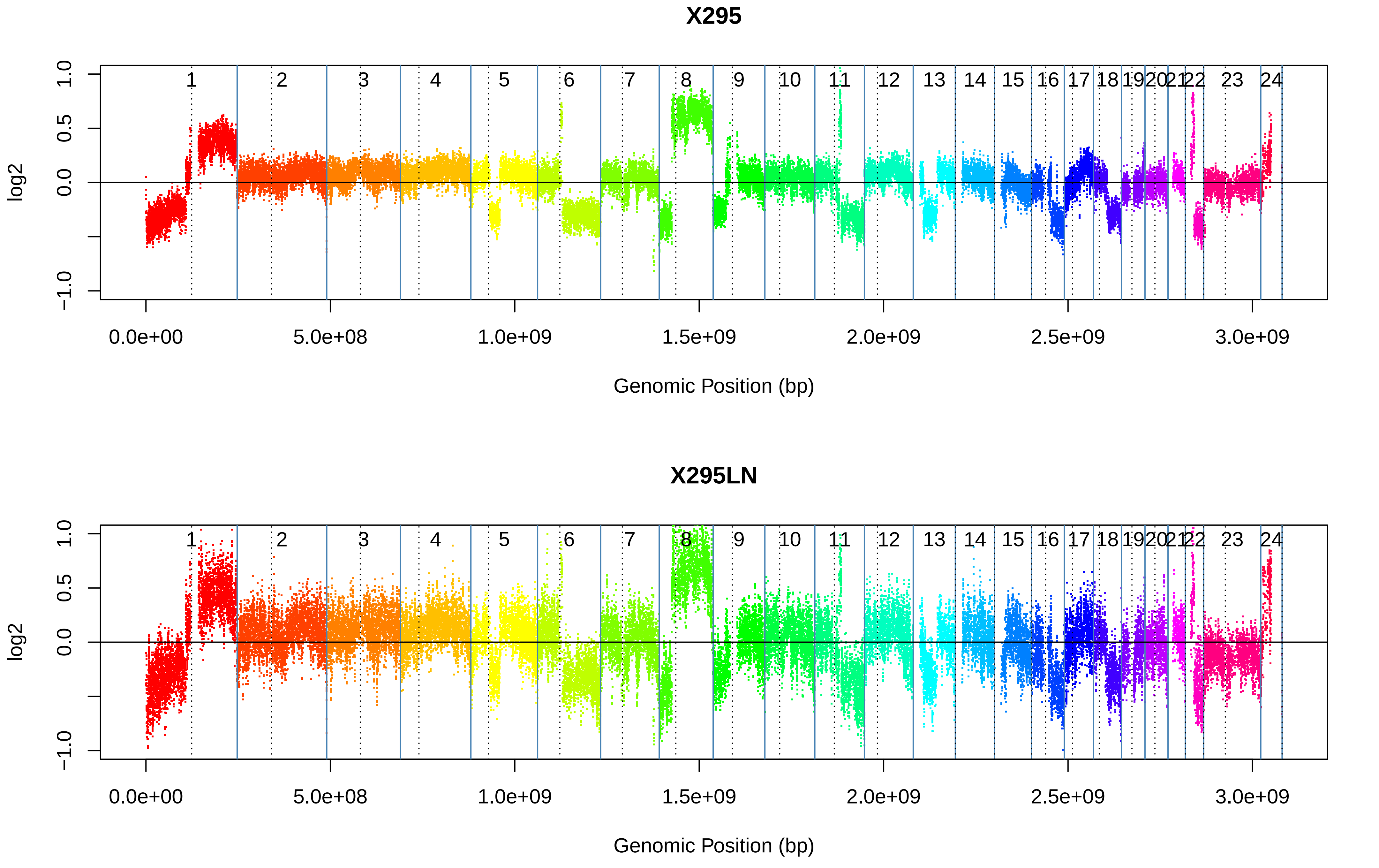
X295
log2
1.0
0.5
0.0
−1.0
1 2 3 4 5 6 7 8 9 10 11 12 13 14 15 16 17 18 19 20 21 22 23 24
0.0e+00
5.0e+08
1.0e+09
1.5e+09
2.0e+09
2.5e+09
3.0e+09
Genomic Position (bp)
X295LN
log2
1.0
0.5
0.0
−1.0
1 2 3 4 5 6 7 8 9 10 11 12 13 14 15 16 17 18 19 20 21 22 23 24
0.0e+00
5.0e+08
1.0e+09
1.5e+09
2.0e+09
2.5e+09
3.0e+09
Genomic Position (bp)

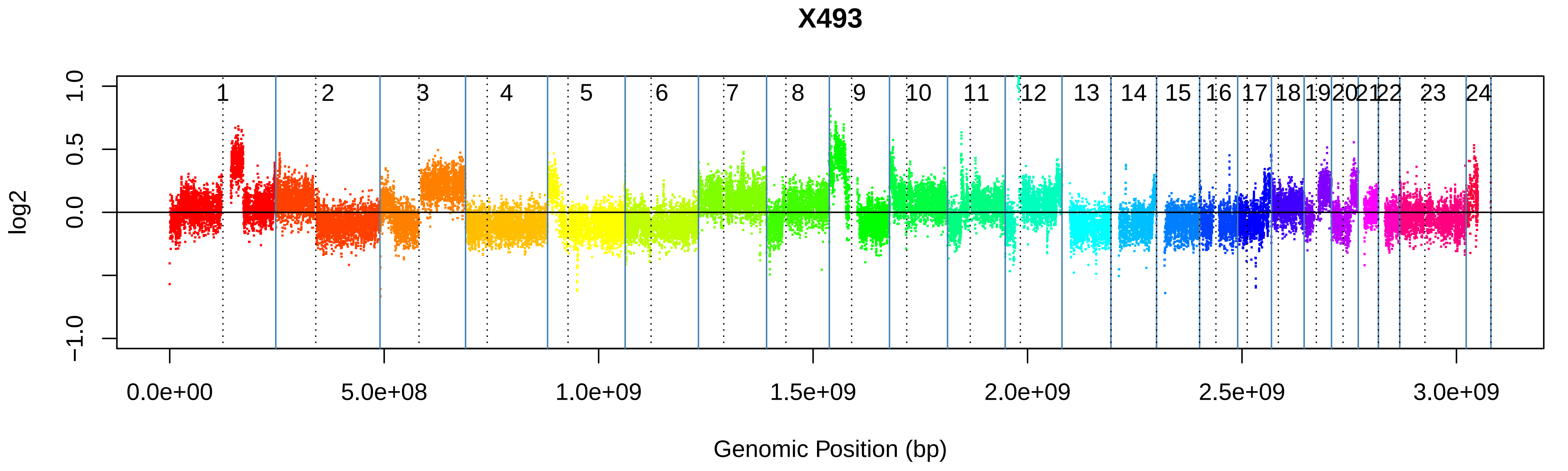
X493
log2
1.0
0.5
0.0
−1.0
1 2 3 4 5 6 7 8 9 10 11 12 13 14 15 16 17 18 19 20 21 22 23 24
0.0e+00
5.0e+08
1.0e+09
1.5e+09
2.0e+09
2.5e+09
3.0e+09
Genomic Position (bp)

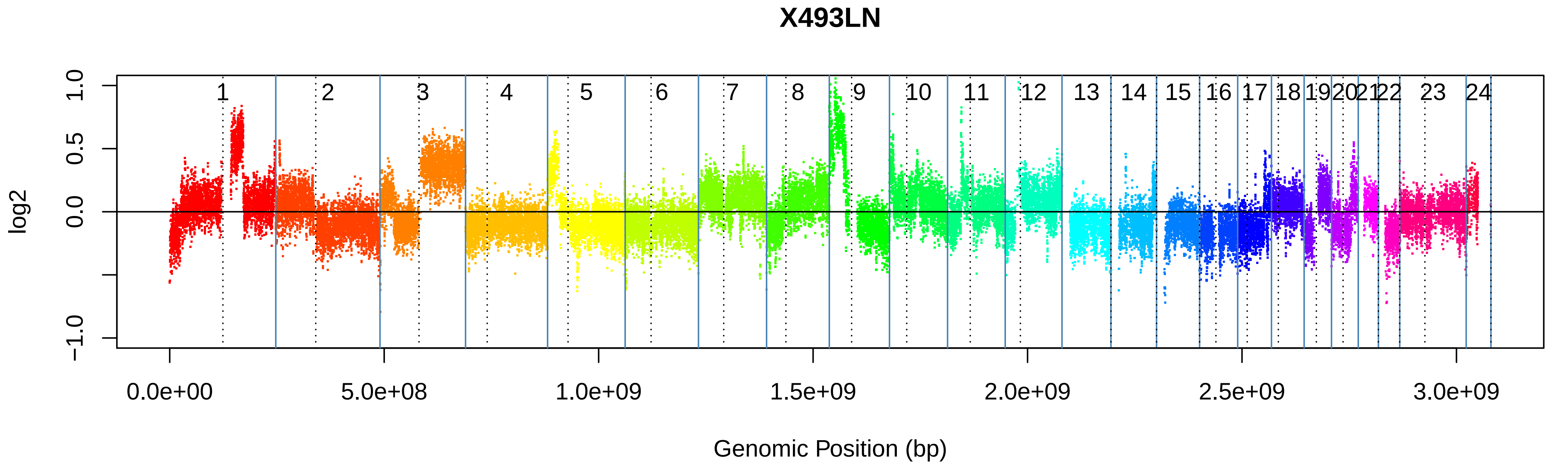
X493LN
log2
1.0
0.5
0.0
−1.0
1 2 3 4 5 6 7 8 9 10 11 12 13 14 15 16 17 18 19 20 21 22 23 24
0.0e+00
5.0e+08
1.0e+09
1.5e+09
2.0e+09
2.5e+09
3.0e+09
Genomic Position (bp)

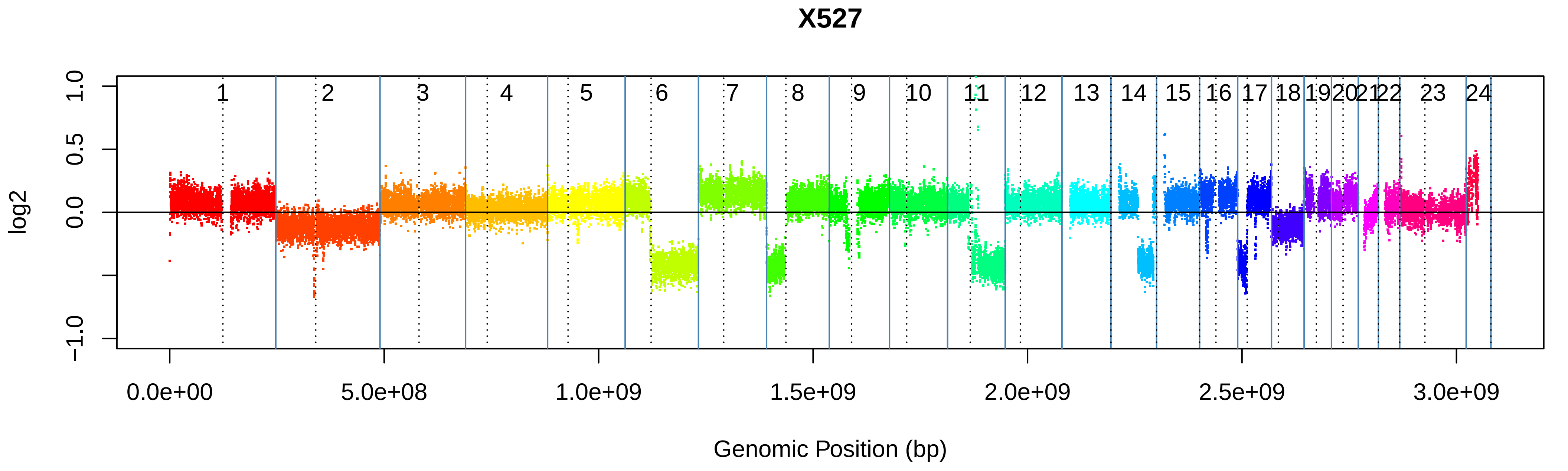
X527
log2
1.0
0.5
0.0
−1.0
1 2 3 4 5 6 7 8 9 10 11 12 13 14 15 16 17 18 19 20 21 22 23 24
0.0e+00
5.0e+08
1.0e+09
1.5e+09
2.0e+09
2.5e+09
3.0e+09
Genomic Position (bp)

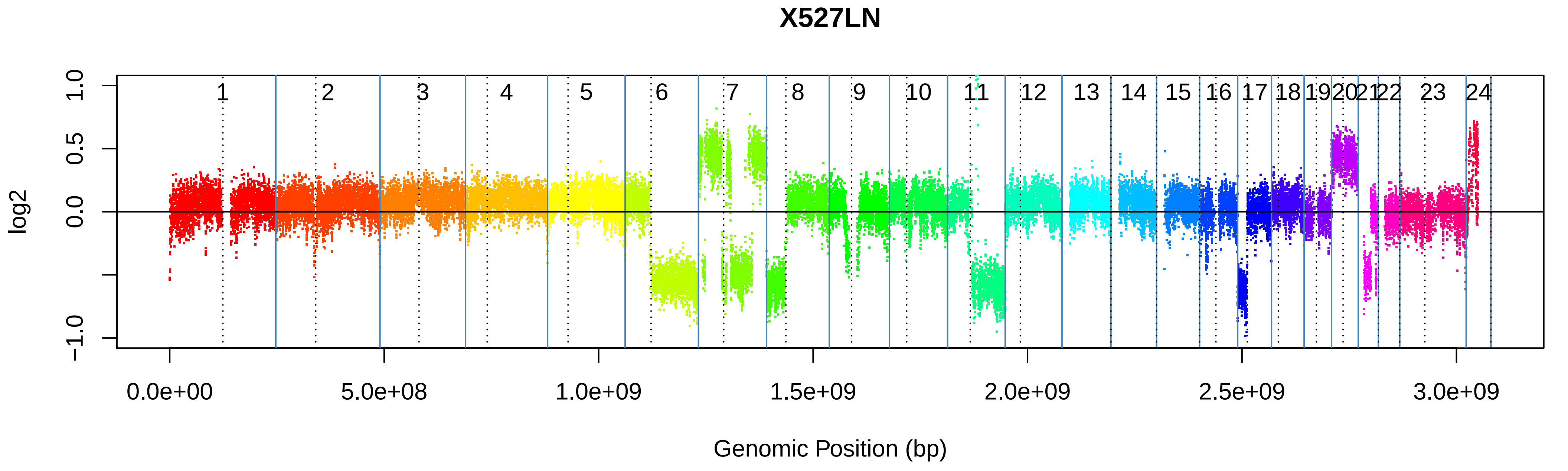
X527LN
log2
1.0
0.5
0.0
−1.0
1 2 3 4 5 6 7 8 9 10 11 12 13 14 15 16 17 18 19 20 21 22 23 24
0.0e+00
5.0e+08
1.0e+09
1.5e+09
2.0e+09
2.5e+09
3.0e+09
Genomic Position (bp)

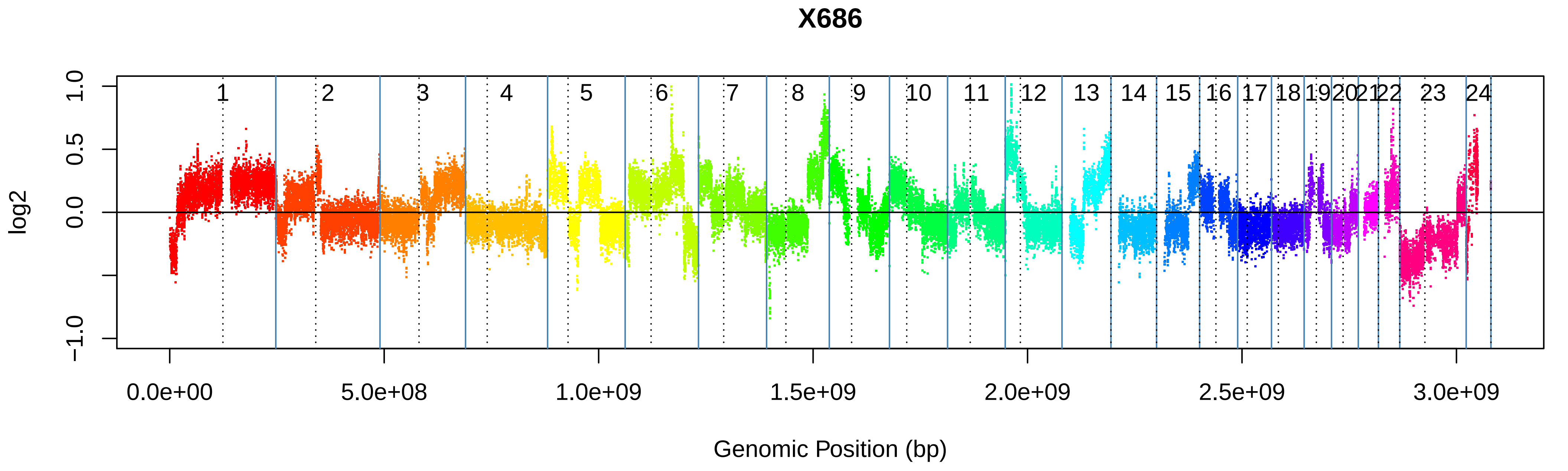
X686
log2
1.0
0.5
0.0
−1.0
1
2
3
4
5
6
7
8
9
10
11
12
13
14
15
16
17
18
19
20
21
22
23
24
0.0e+00
5.0e+08
1.0e+09
1.5e+09
2.0e+09
2.5e+09
3.0e+09
Genomic Position (bp)

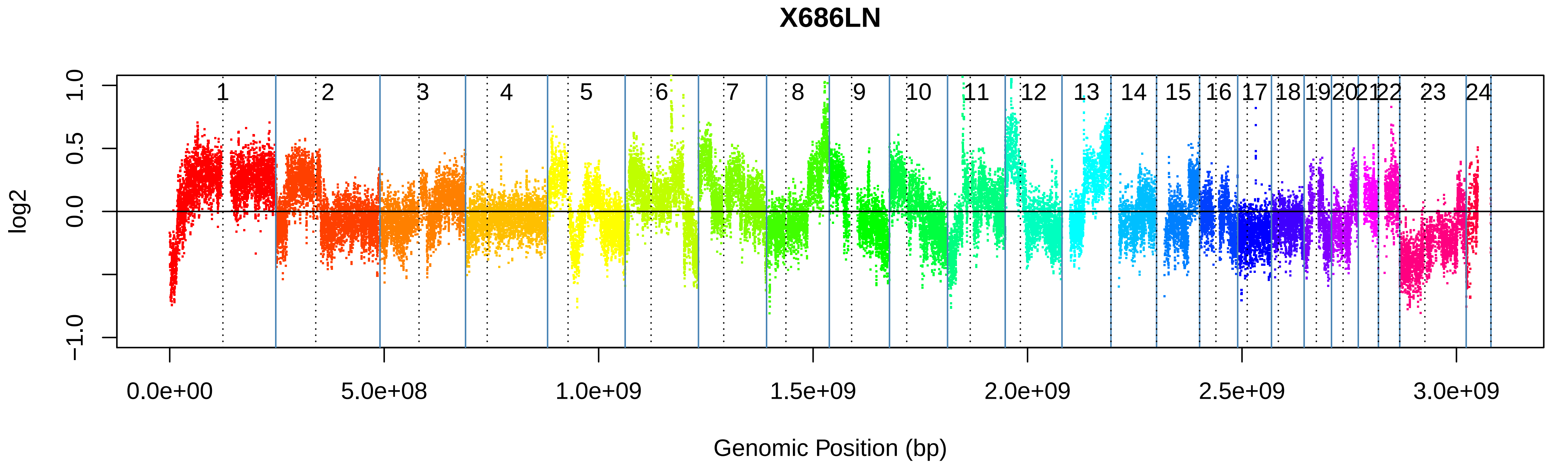
X686LN
log2
1.0
0.5
0.0
−1.0
1
2
3
4
5
6
7
8
9
10
11
12
13
14
15
16
17
18
19
20
21
22
23
24
0.0e+00
5.0e+08
1.0e+09
1.5e+09
2.0e+09
2.5e+09
3.0e+09
Genomic Position (bp)

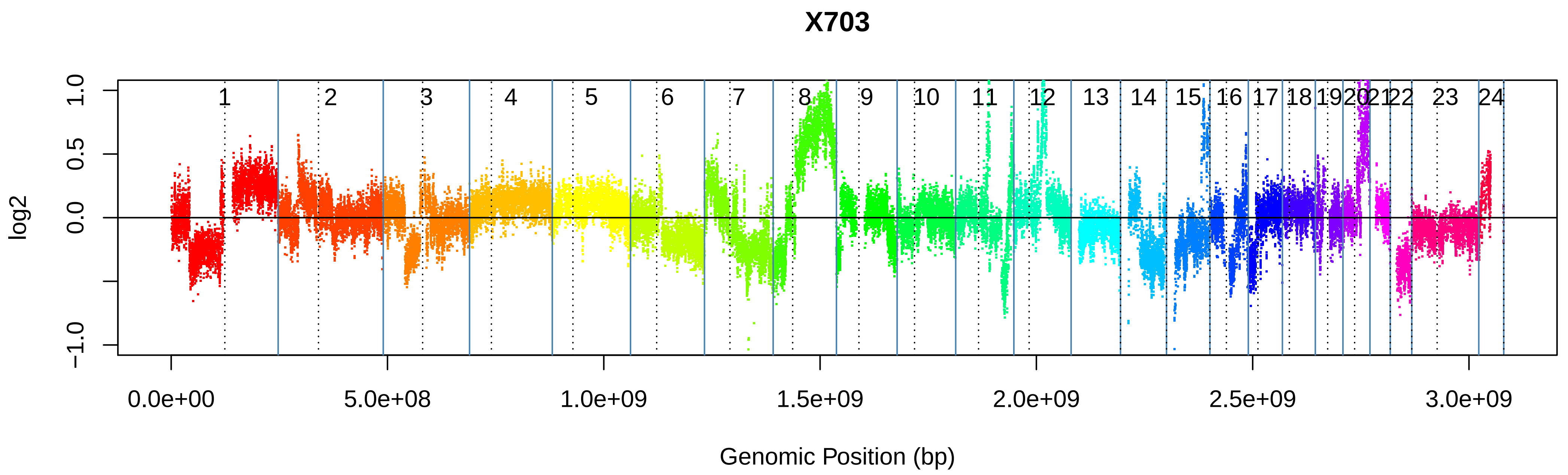
X703
1.0
0.5
0.0
−1.0
log2
1
2
3
4
5
6
7
8
9
10
11
12
13
14
15
16
17
18
19
20
21
22
23
24
0.0e+00
5.0e+08
1.0e+09
1.5e+09
2.0e+09
2.5e+09
3.0e+09
Genomic Position (bp)

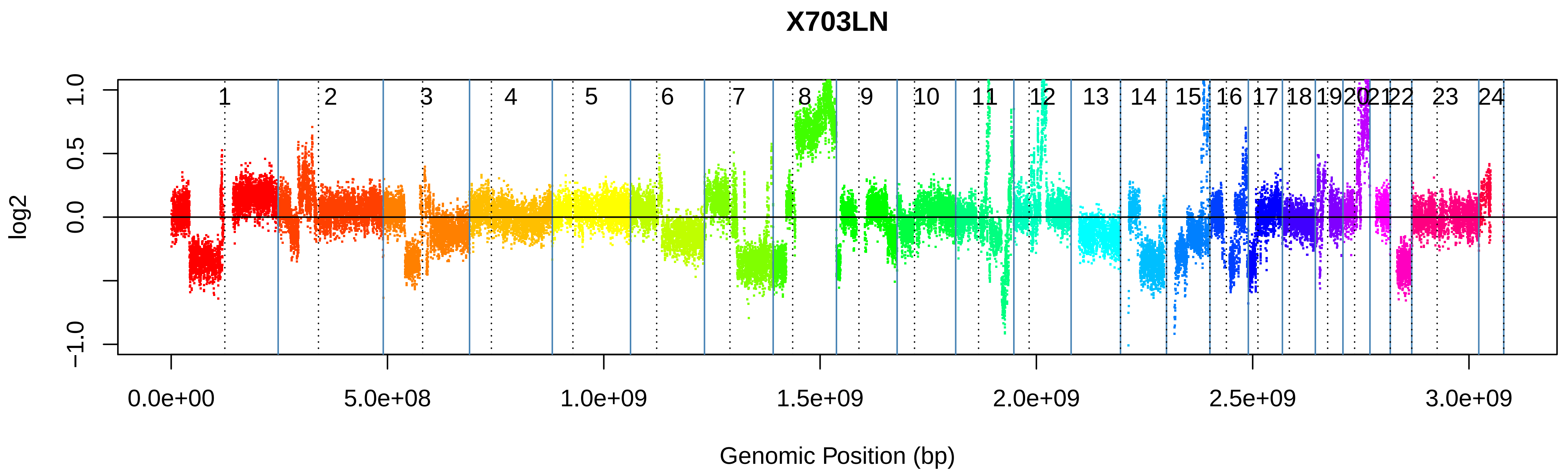
X703LN
1.0
0.5
0.0
−1.0
log2
1
2
3
4
5
6
7
8
9
10
11
12
13
14
15
16
17
18
19
20
21
22
23
24
0.0e+00
5.0e+08
1.0e+09
1.5e+09
2.0e+09
2.5e+09
3.0e+09
Genomic Position (bp)

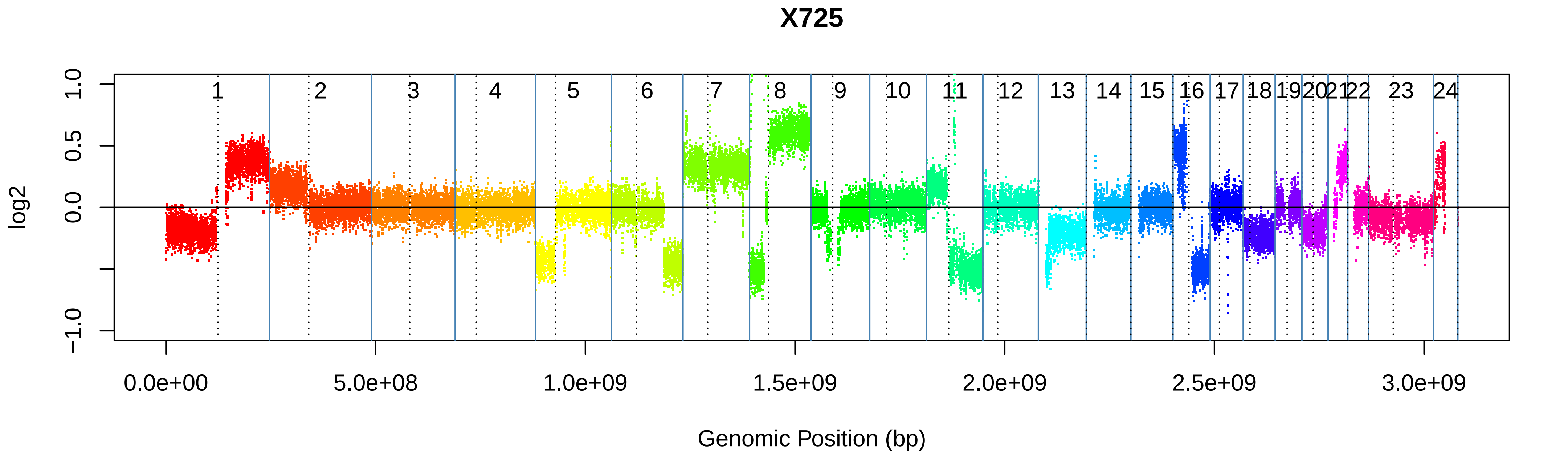
X725
log2
1.0
0.5
0.0
−1.0
1 2 3 4 5 6 7 8 9 10 11 12 13 14 15 16 17 18 19 20 21 22 23 24
0.0e+00
5.0e+08
1.0e+09
1.5e+09
2.0e+09
2.5e+09
3.0e+09
Genomic Position (bp)

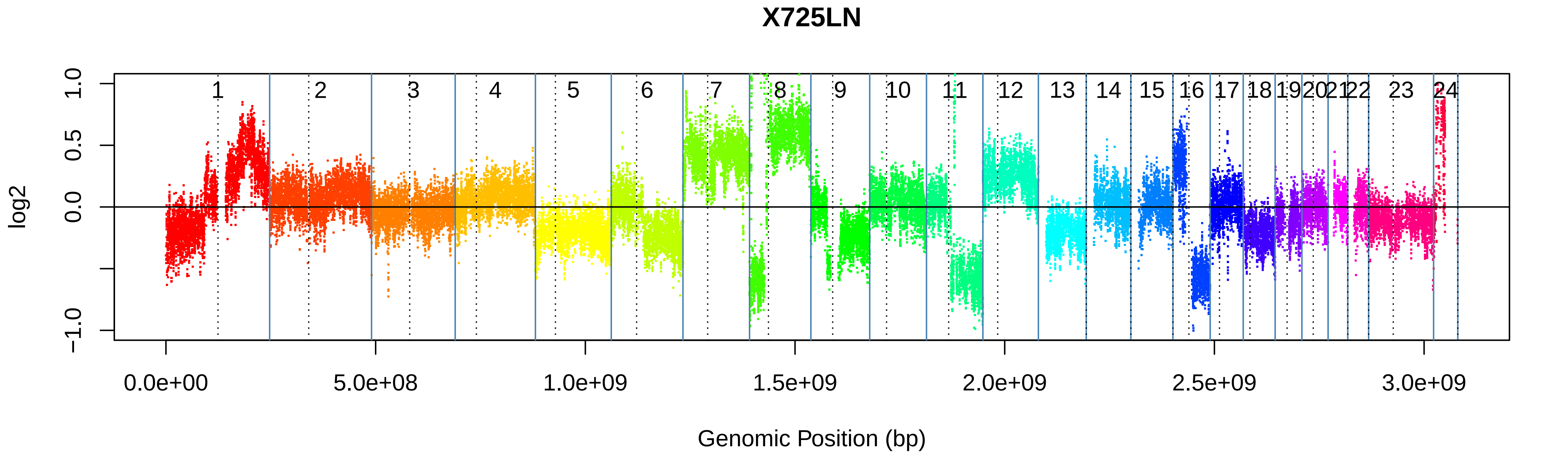
X725LN
log2
1.0
0.5
0.0
−1.0
1 2 3 4 5 6 7 8 9 10 11 12 13 14 15 16 17 18 19 20 21 22 23 24
0.0e+00
5.0e+08
1.0e+09
1.5e+09
2.0e+09
2.5e+09
3.0e+09
Genomic Position (bp)

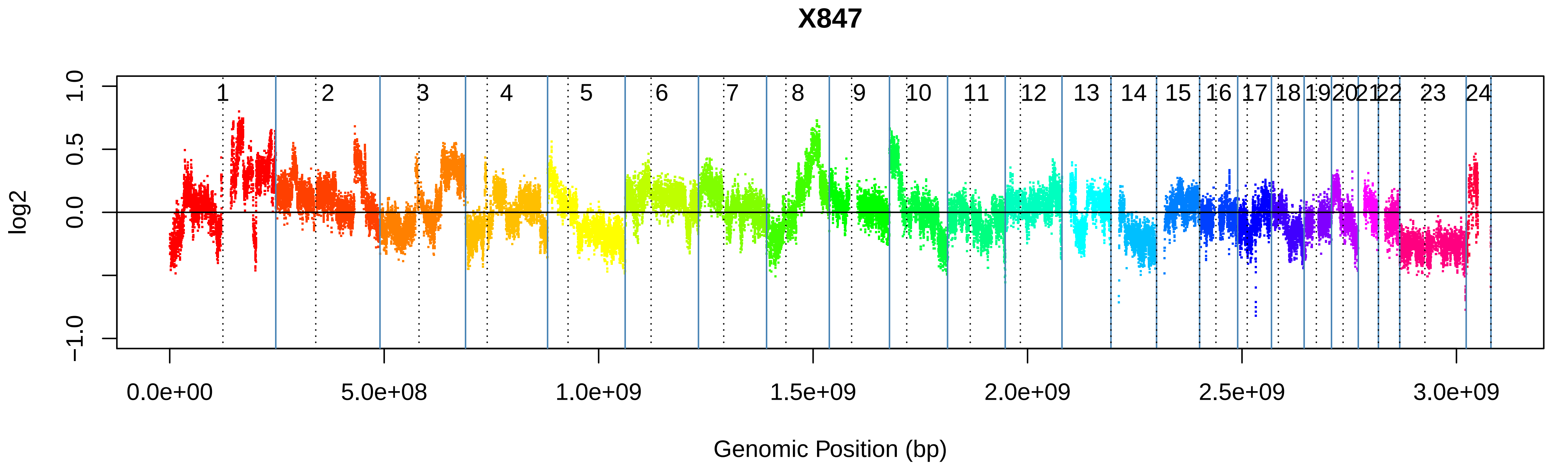
X847
log2
1.0
0.5
0.0
−1.0
1 2 3 4 5 6 7 8 9 10 11 12 13 14 15 16 17 18 19 20 21 22 23 24
0.0e+00
5.0e+08
1.0e+09
1.5e+09
2.0e+09
2.5e+09
3.0e+09
Genomic Position (bp)

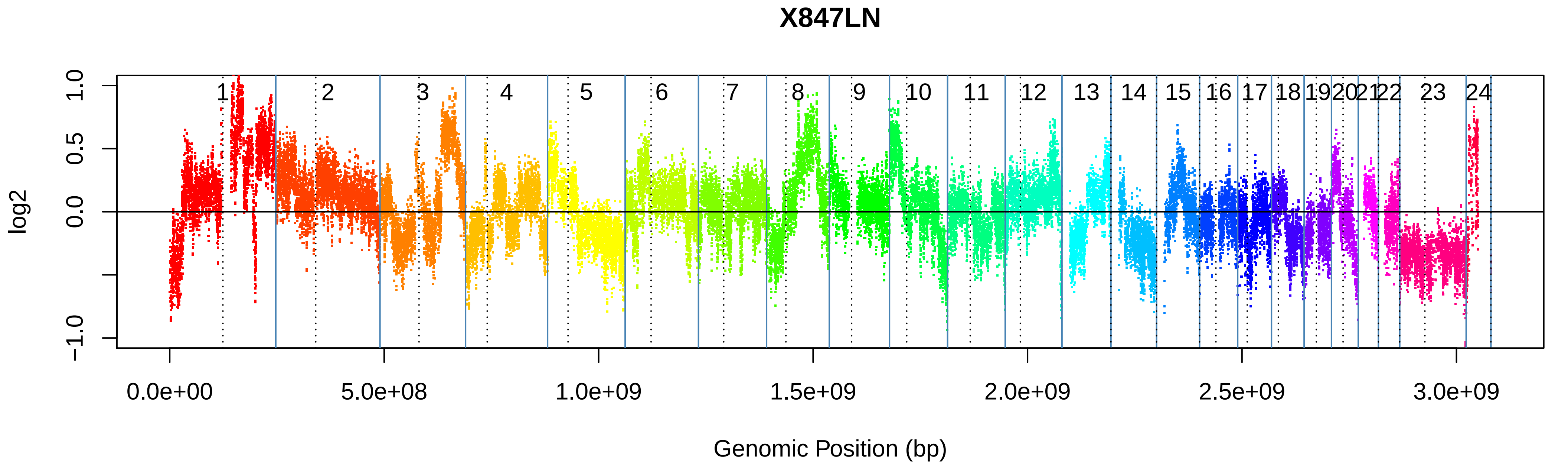
X847LN
log2
1.0
0.5
0.0
−1.0
1 2 3 4 5 6 7 8 9 10 11 12 13 14 15 16 17 18 19 20 21 22 23 24
0.0e+00
5.0e+08
1.0e+09
1.5e+09
2.0e+09
2.5e+09
3.0e+09
Genomic Position (bp)

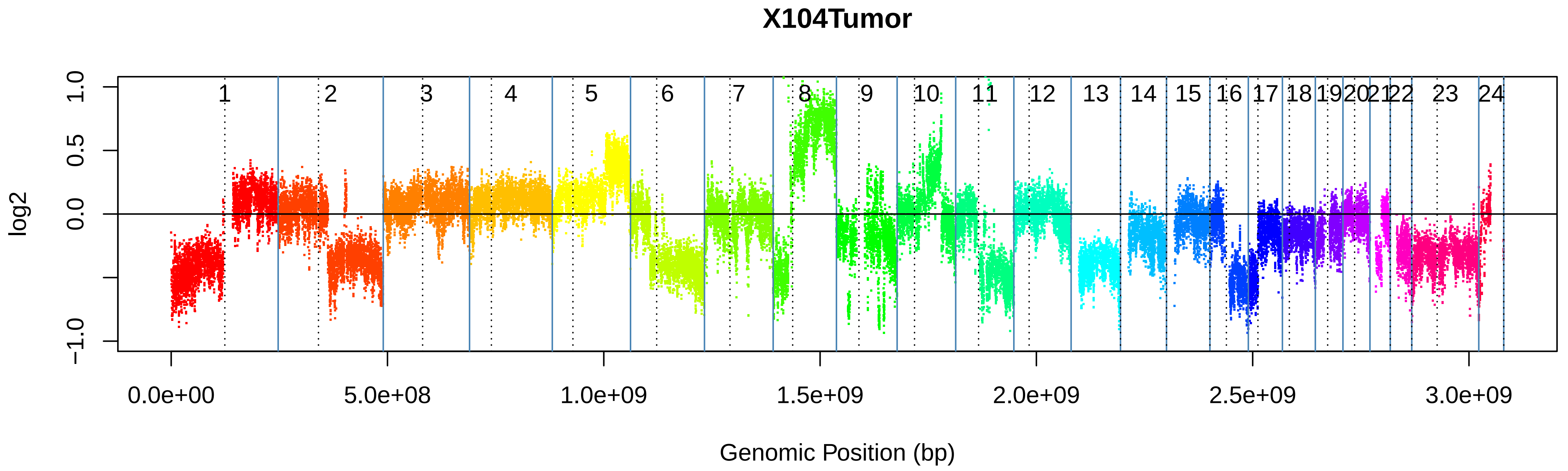
X104Tumor
log2
1.0
0.5
0.0
−1.0
1
2
3
4
5
6
7
8
9
10
11
12
13
14
15
16
17
18
19
20
21
22
23
24
0.0e+00
5.0e+08
1.0e+09
1.5e+09
2.0e+09
2.5e+09
3.0e+09
Genomic Position (bp)

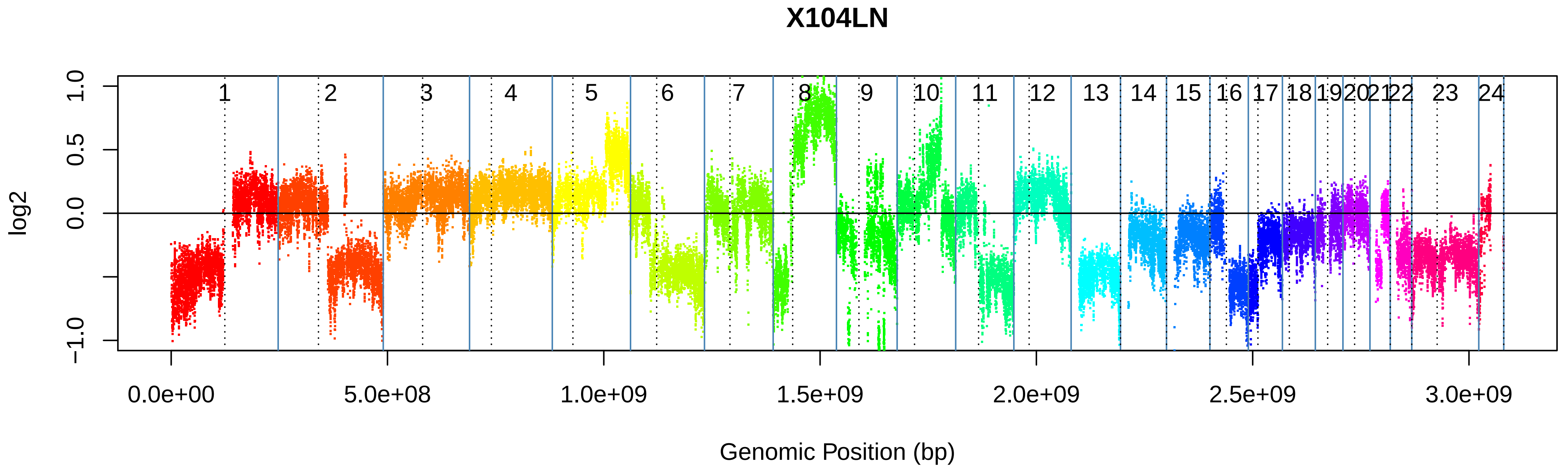
X104LN
log2
1.0
0.5
0.0
−1.0
1
2
3
4
5
6
7
8
9
10
11
12
13
14
15
16
17
18
19
20
21
22
23
24
0.0e+00
5.0e+08
1.0e+09
1.5e+09
2.0e+09
2.5e+09
3.0e+09
Genomic Position (bp)

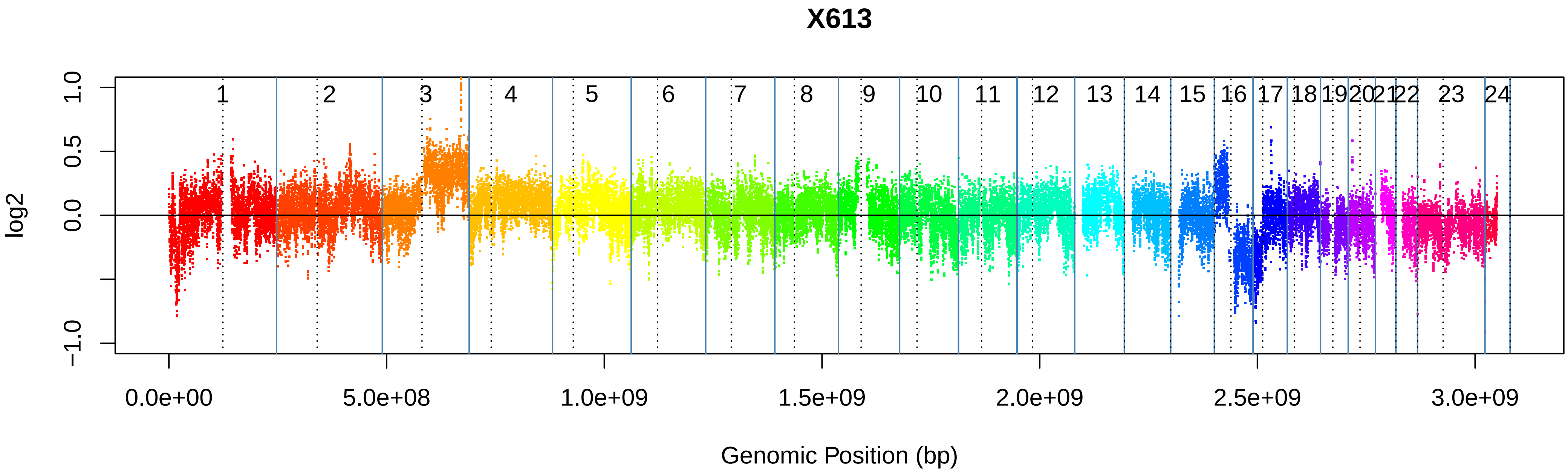
X613
log2
1.0
0.5
0.0
−1.0
1 2 3 4 5 6 7 8 9 10 11 12 13 14 15 16 17 18 19 20 21 22 23 24
0.0e+00
5.0e+08
1.0e+09
1.5e+09
2.0e+09
2.5e+09
3.0e+09
Genomic Position (bp)

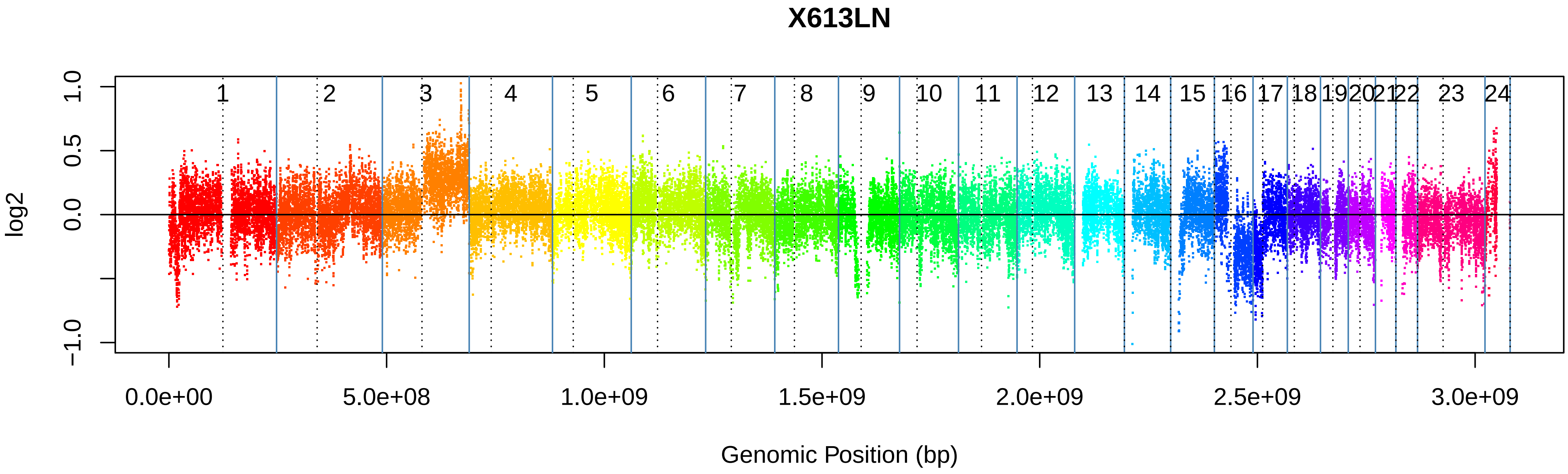
X613LN
log2
1.0
0.5
0.0
−1.0
1 2 3 4 5 6 7 8 9 10 11 12 13 14 15 16 17 18 19 20 21 22 23 24
0.0e+00
5.0e+08
1.0e+09
1.5e+09
2.0e+09
2.5e+09
3.0e+09
Genomic Position (bp)

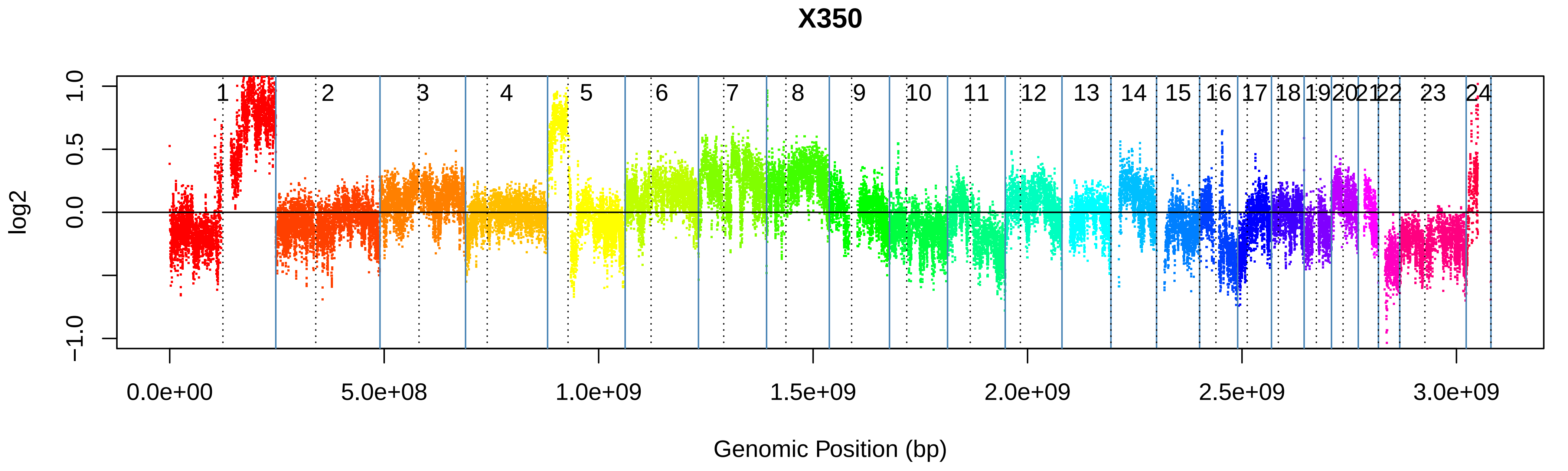
X350
log2
Genomic Position (bp)
1 2 3 4 5 6 7 8 9 10 11 12 13 14 15 16 17 18 19 20 21 22 23 24

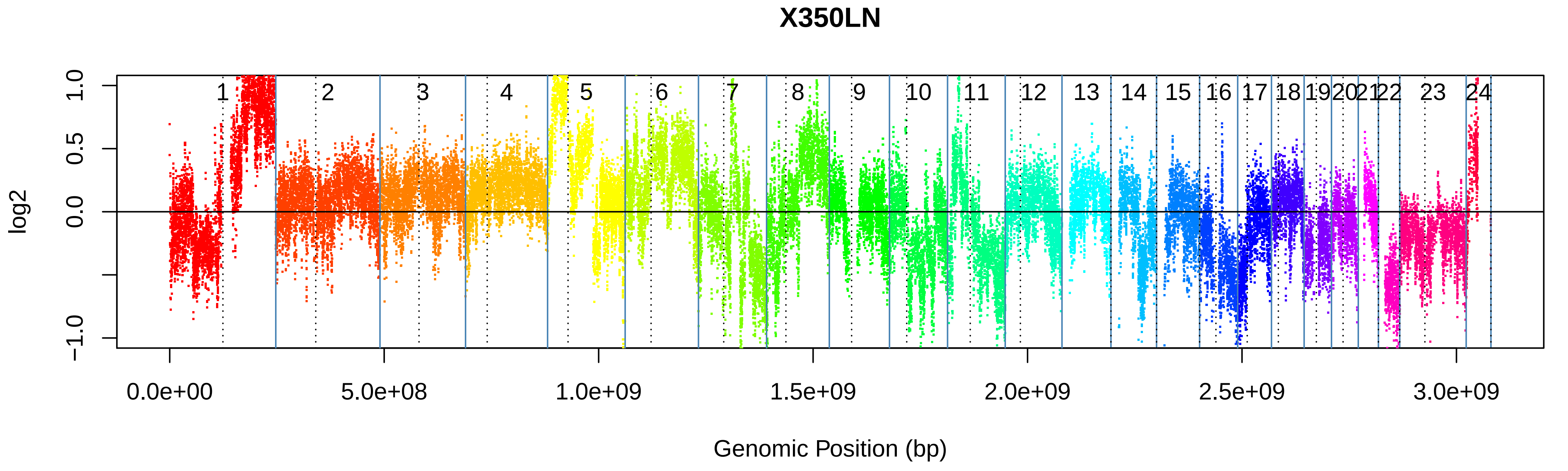
X350LN
log2
Genomic Position (bp)
1 2 3 4 5 6 7 8 9 10 11 12 13 14 15 16 17 18 19 20 21 22 23 24

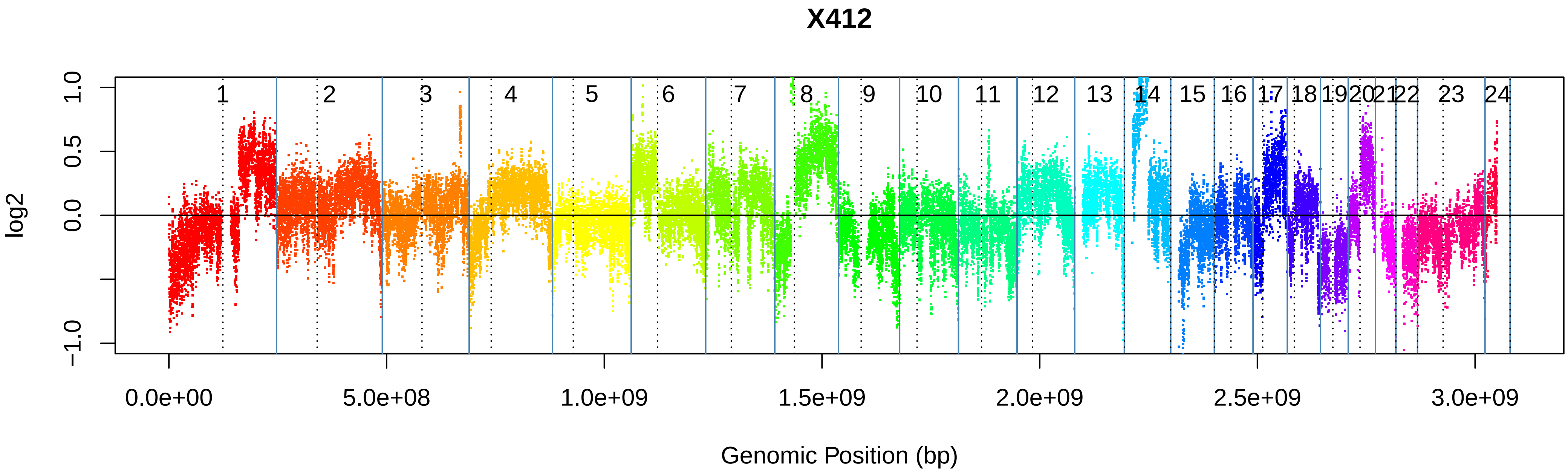
X412
log2
1.0
0.5
0.0
−1.0
1
2
3
4
5
6
7
8
9
10
11
12
13
14
15
16
17
18
19
20
21
22
23
24
0.0e+00
5.0e+08
1.0e+09
1.5e+09
2.0e+09
2.5e+09
3.0e+09
Genomic Position (bp)

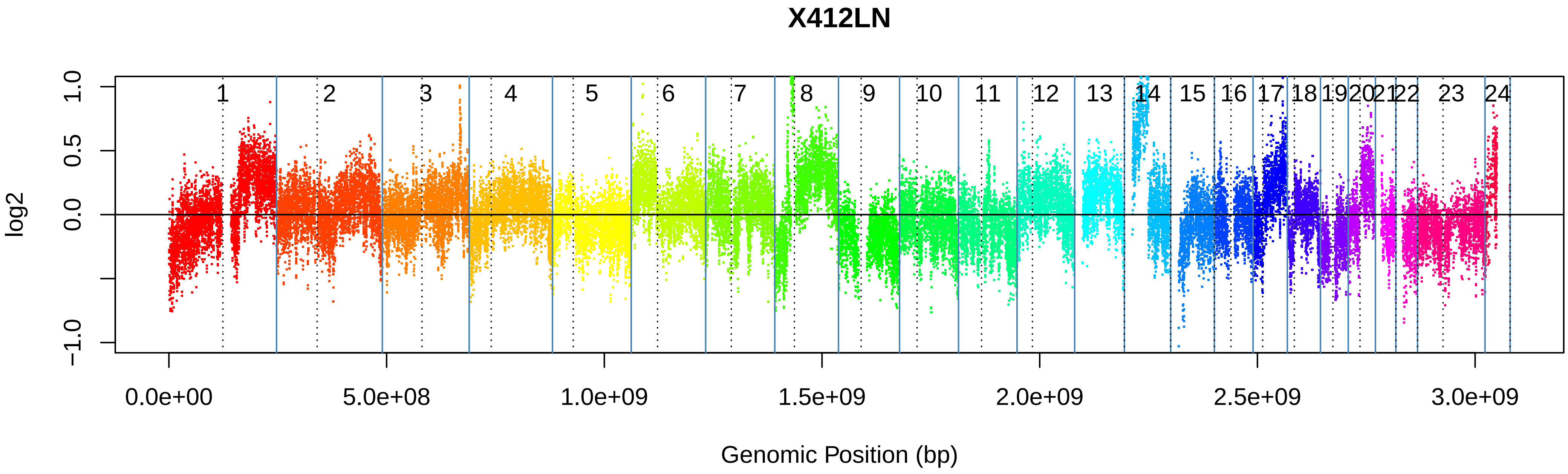
X412LN
log2
1.0
0.5
0.0
−1.0
1
2
3
4
5
6
7
8
9
10
11
12
13
14
15
16
17
18
19
20
21
22
23
24
0.0e+00
5.0e+08
1.0e+09
1.5e+09
2.0e+09
2.5e+09
3.0e+09
Genomic Position (bp)

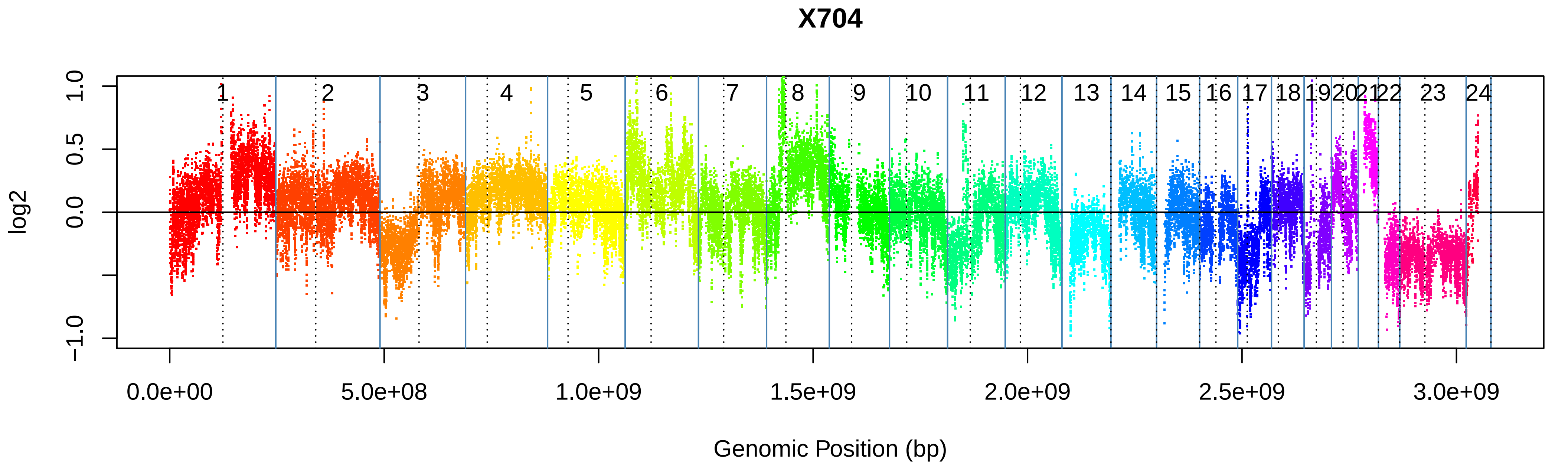
X704
log2
1.0
0.5
0.0
−1.0
1 2 3 4 5 6 7 8 9 10 11 12 13 14 15 16 17 18 19 20 21 22 23 24
0.0e+00
5.0e+08
1.0e+09
1.5e+09
2.0e+09
2.5e+09
3.0e+09
Genomic Position (bp)

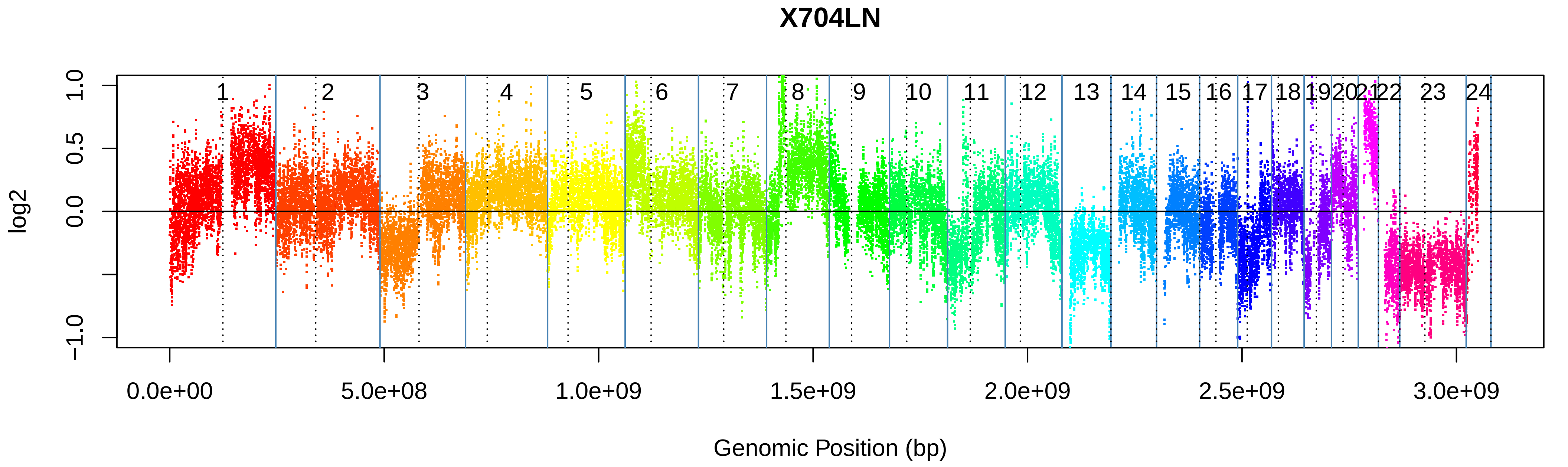
X704LN
log2
1.0
0.5
0.0
−1.0
1 2 3 4 5 6 7 8 9 10 11 12 13 14 15 16 17 18 19 20 21 22 23 24
0.0e+00
5.0e+08
1.0e+09
1.5e+09
2.0e+09
2.5e+09
3.0e+09
Genomic Position (bp)

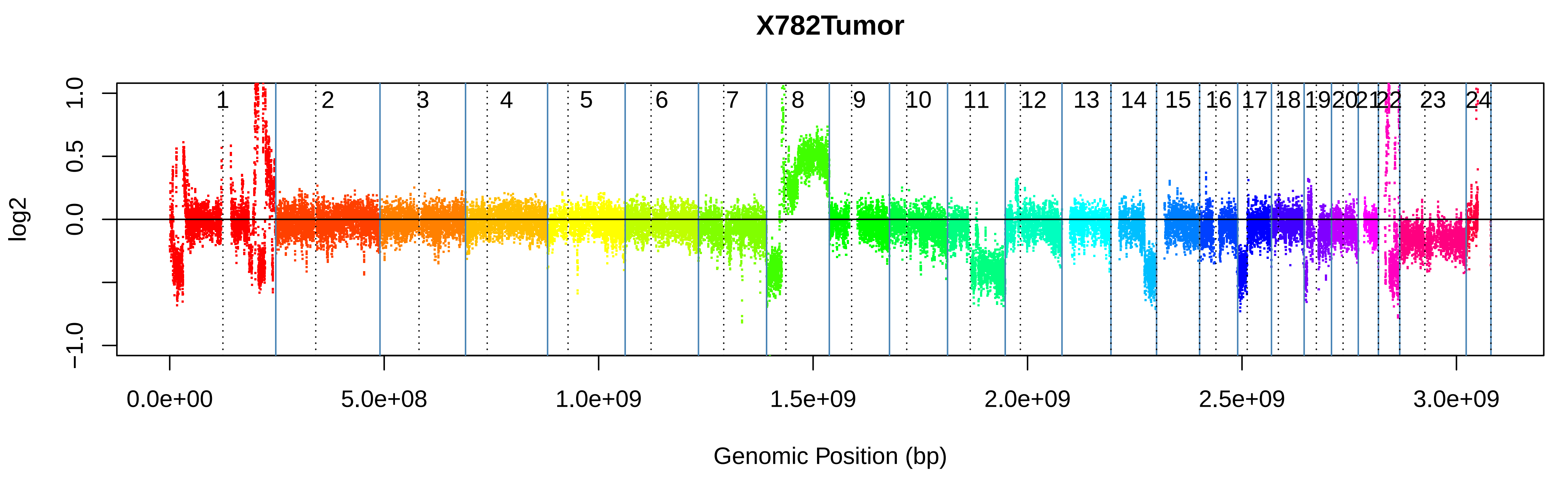
X782Tumor
log2
1.0
0.5
0.0
−1.0
1
2
3
4
5
6
7
8
9
10
11
12
13
14
15
16
17
18
19
20
21
22
23
24
0.0e+00
5.0e+08
1.0e+09
1.5e+09
2.0e+09
2.5e+09
3.0e+09
Genomic Position (bp)

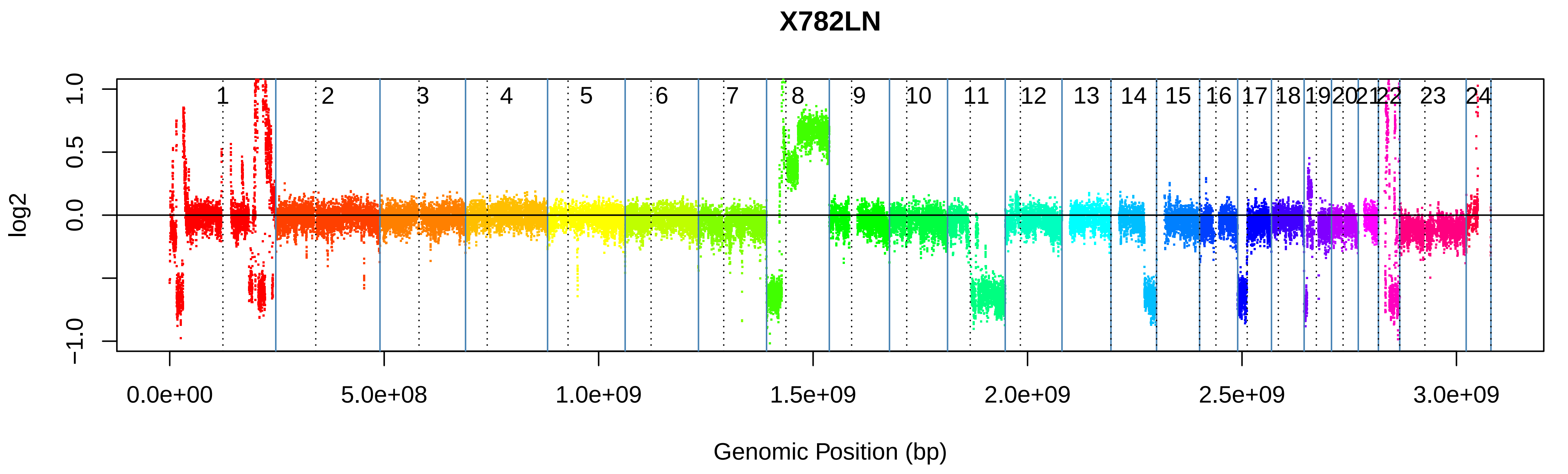
X782LN
log2
1.0
0.5
0.0
−1.0
1
2
3
4
5
6
7
8
9
10
11
12
13
14
15
16
17
18
19
20
21
22
23
24
0.0e+00
5.0e+08
1.0e+09
1.5e+09
2.0e+09
2.5e+09
3.0e+09
Genomic Position (bp)

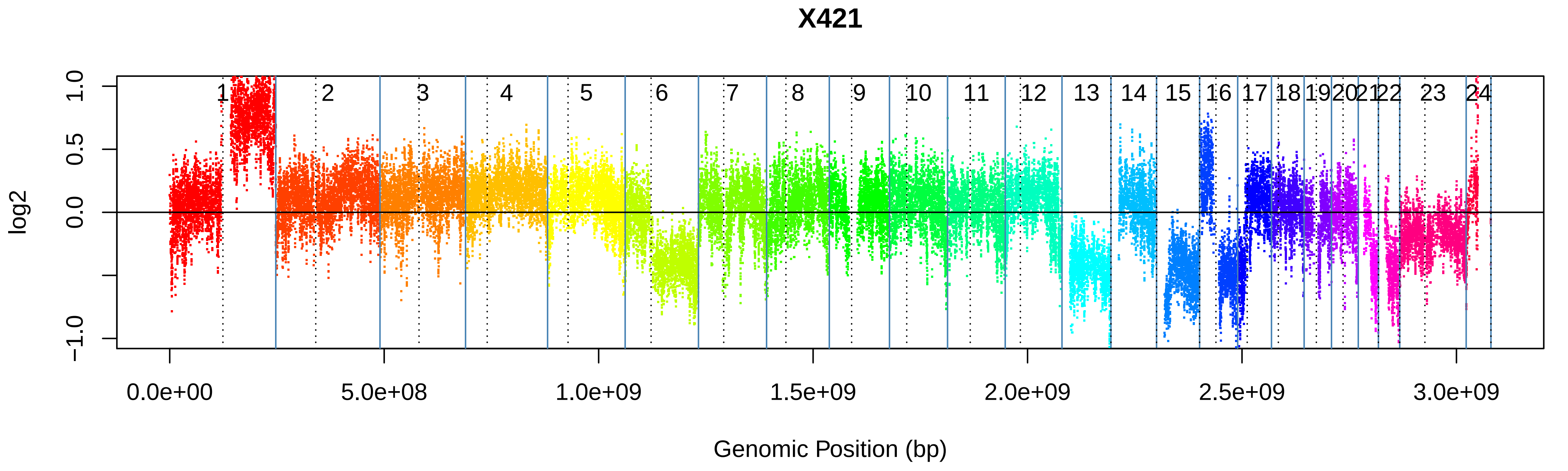
X421
log2
1.0
0.5
0.0
−1.0
1
2
3
4
5
6
7
8
9
10
11
12
13
14
15
16
17
18
19
20
21
22
23
24
0.0e+00
5.0e+08
1.0e+09
1.5e+09
2.0e+09
2.5e+09
3.0e+09
Genomic Position (bp)

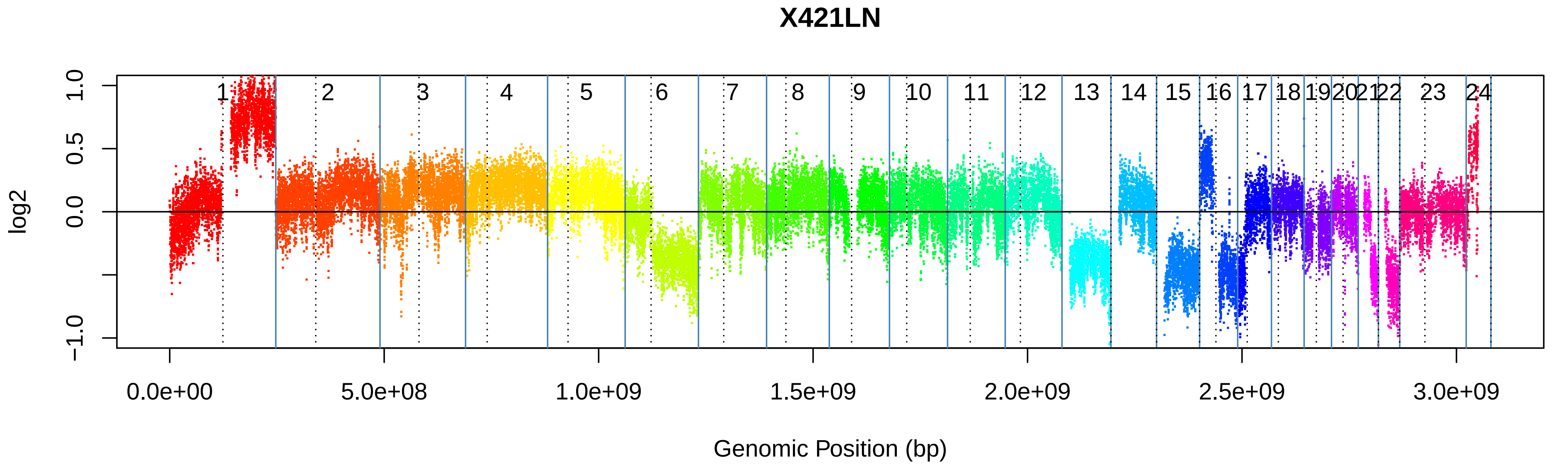
X421LN
log2
1.0
0.5
0.0
−1.0
1
2
3
4
5
6
7
8
9
10
11
12
13
14
15
16
17
18
19
20
21
22
23
24
0.0e+00
5.0e+08
1.0e+09
1.5e+09
2.0e+09
2.5e+09
3.0e+09
Genomic Position (bp)

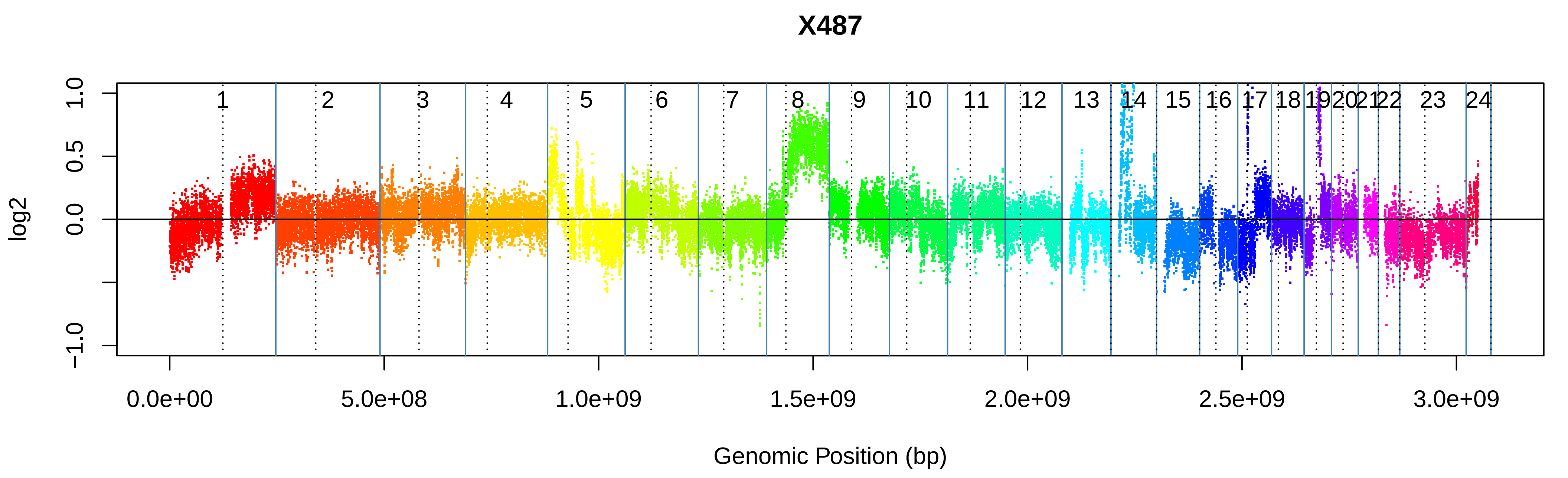
X487
log2
1.0
0.5
0.0
−1.0
1
2
3
4
5
6
7
8
9
10
11
12
13
14
15
16
17
18
19
20
21
22
23
24
0.0e+00
5.0e+08
1.0e+09
1.5e+09
2.0e+09
2.5e+09
3.0e+09
Genomic Position (bp)

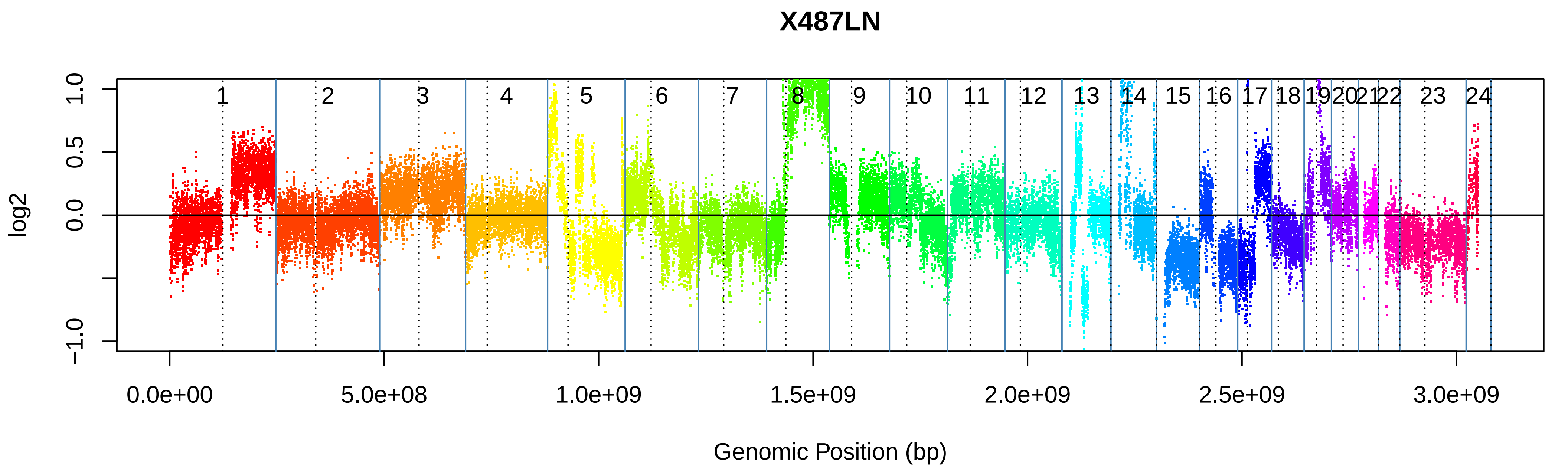
X487LN
log2
1.0
0.5
0.0
−1.0
1
2
3
4
5
6
7
8
9
10
11
12
13
14
15
16
17
18
19
20
21
22
23
24
0.0e+00
5.0e+08
1.0e+09
1.5e+09
2.0e+09
2.5e+09
3.0e+09
Genomic Position (bp)

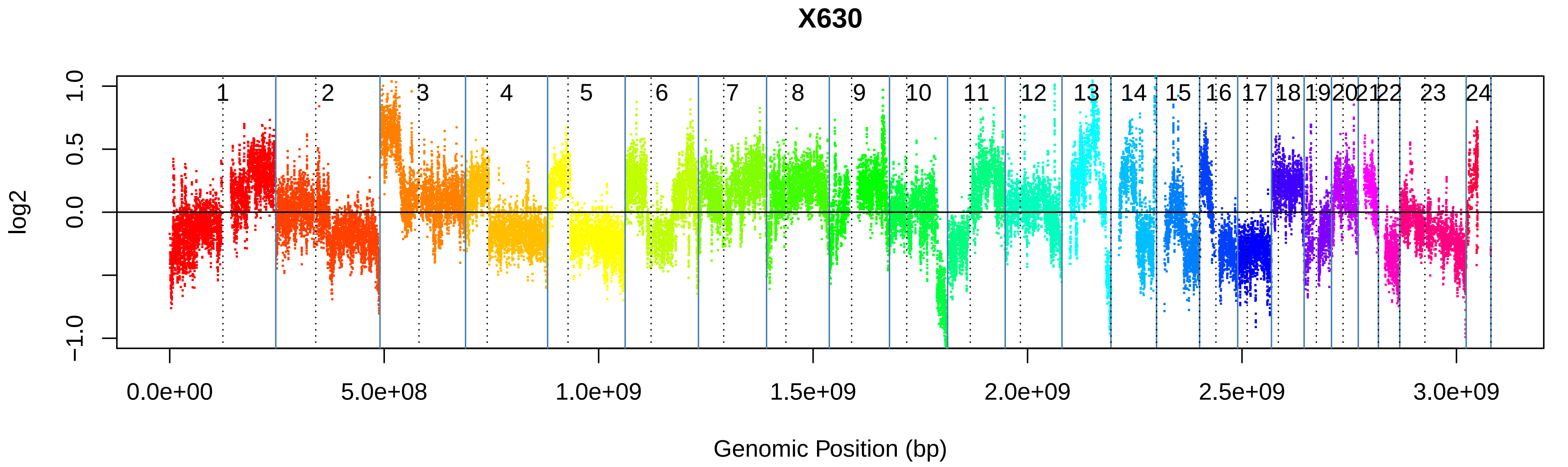
X630
log2
1.0
0.5
0.0
−1.0
1 2 3 4 5 6 7 8 9 10 11 12 13 14 15 16 17 18 19 20 21 22 23 24
0.0e+00
5.0e+08
1.0e+09
1.5e+09
2.0e+09
2.5e+09
3.0e+09
Genomic Position (bp)

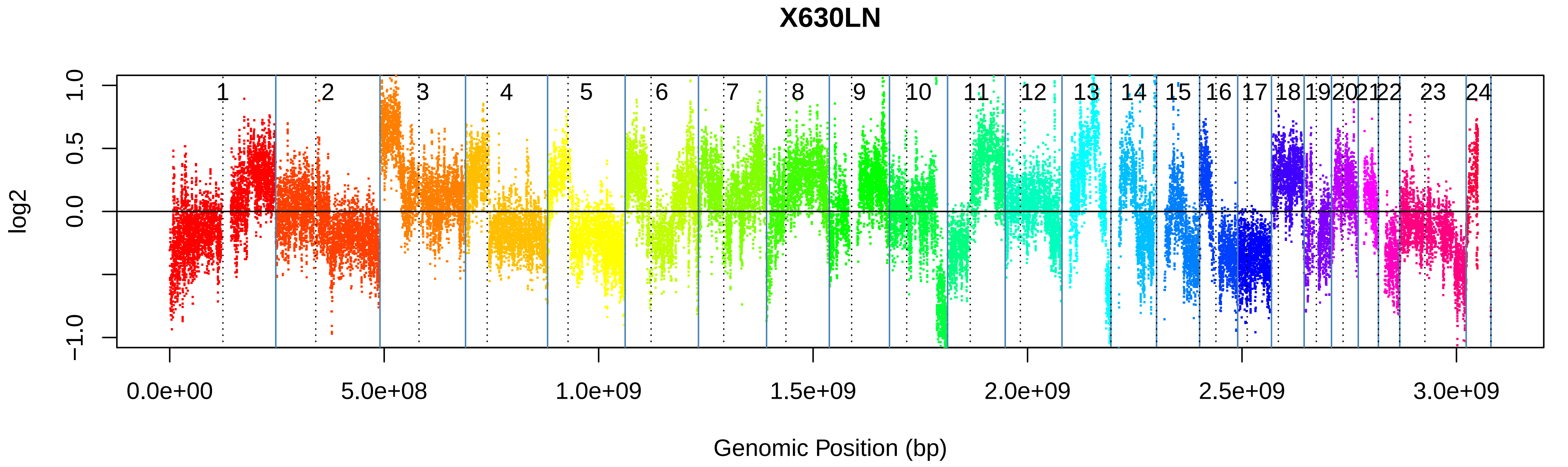
X630LN
log2
1.0
0.5
0.0
−1.0
1 2 3 4 5 6 7 8 9 10 11 12 13 14 15 16 17 18 19 20 21 22 23 24
0.0e+00
5.0e+08
1.0e+09
1.5e+09
2.0e+09
2.5e+09
3.0e+09
Genomic Position (bp)

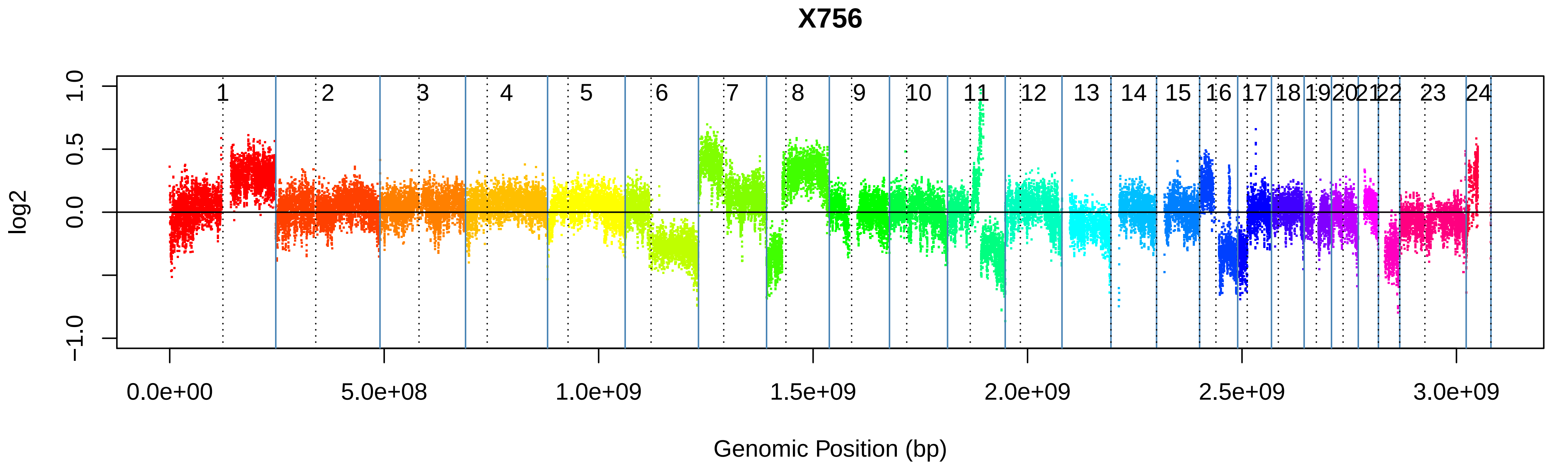
X756
log2
1.0
0.5
0.0
−1.0
1
2
3
4
5
6
7
8
9
10
11
12
13
14
15
16
17
18
19
20
21
22
23
24
0.0e+00
5.0e+08
1.0e+09
1.5e+09
2.0e+09
2.5e+09
3.0e+09
Genomic Position (bp)

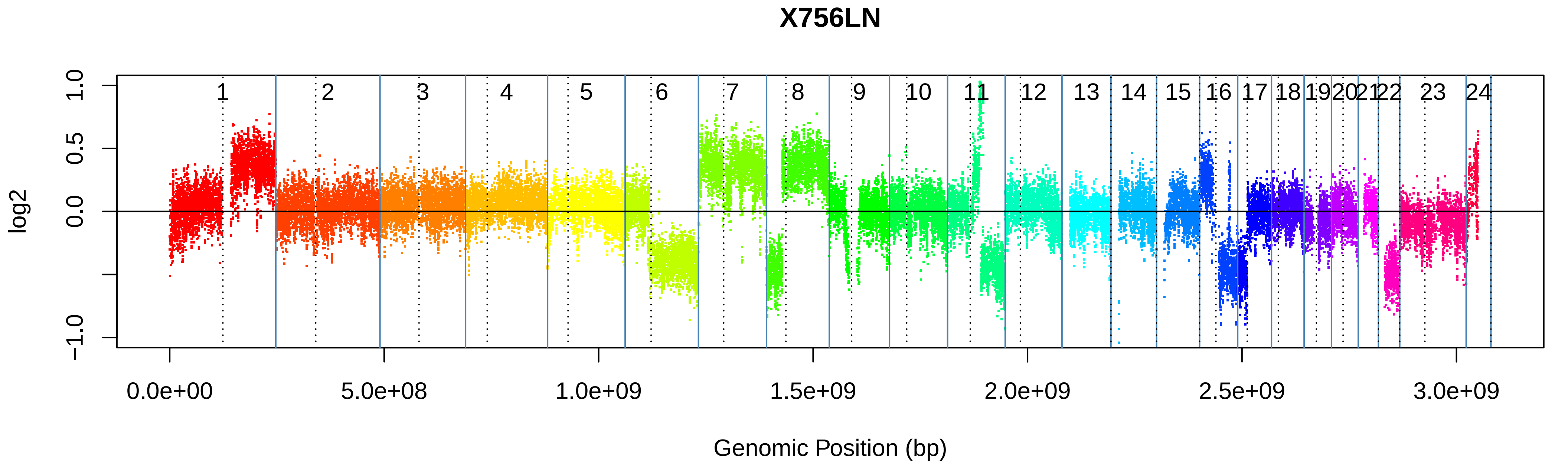
X756LN
log2
1.0
0.5
0.0
−1.0
1
2
3
4
5
6
7
8
9
10
11
12
13
14
15
16
17
18
19
20
21
22
23
24
0.0e+00
5.0e+08
1.0e+09
1.5e+09
2.0e+09
2.5e+09
3.0e+09
Genomic Position (bp)

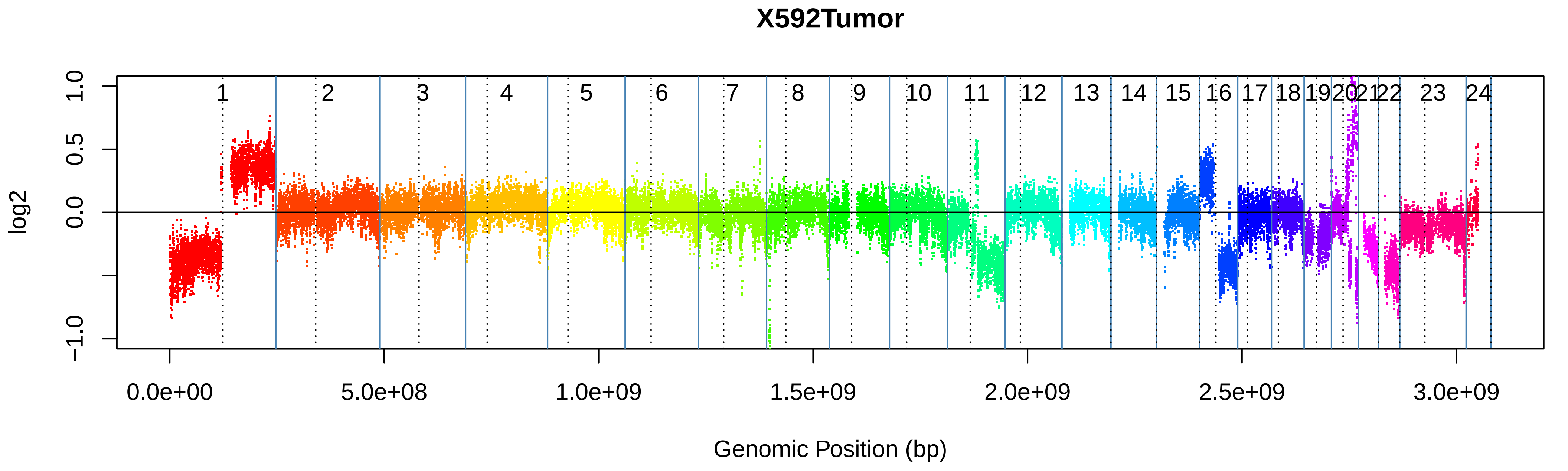
X592Tumor
log2
1.0
0.5
0.0
−1.0
1
2
3
4
5
6
7
8
9
10
11
12
13
14
15
16
17
18
19
20
21
22
23
24
0.0e+00
5.0e+08
1.0e+09
1.5e+09
2.0e+09
2.5e+09
3.0e+09
Genomic Position (bp)

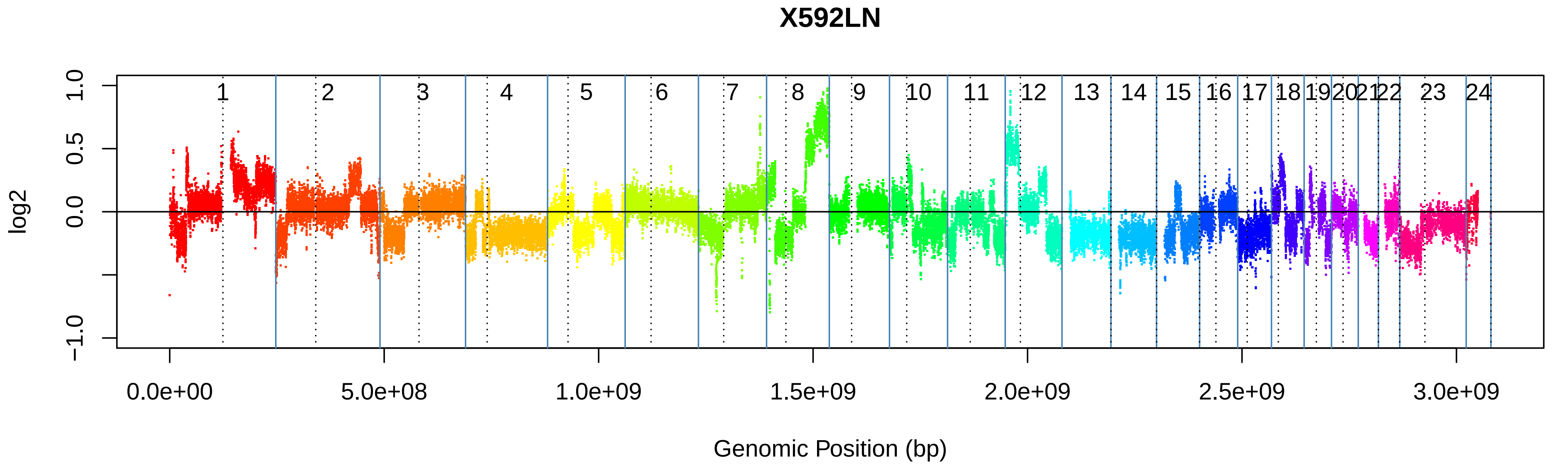
X592LN
log2
1.0
0.5
0.0
−1.0
1
2
3
4
5
6
7
8
9
10
11
12
13
14
15
16
17
18
19
20
21
22
23
24
0.0e+00
5.0e+08
1.0e+09
1.5e+09
2.0e+09
2.5e+09
3.0e+09
Genomic Position (bp)

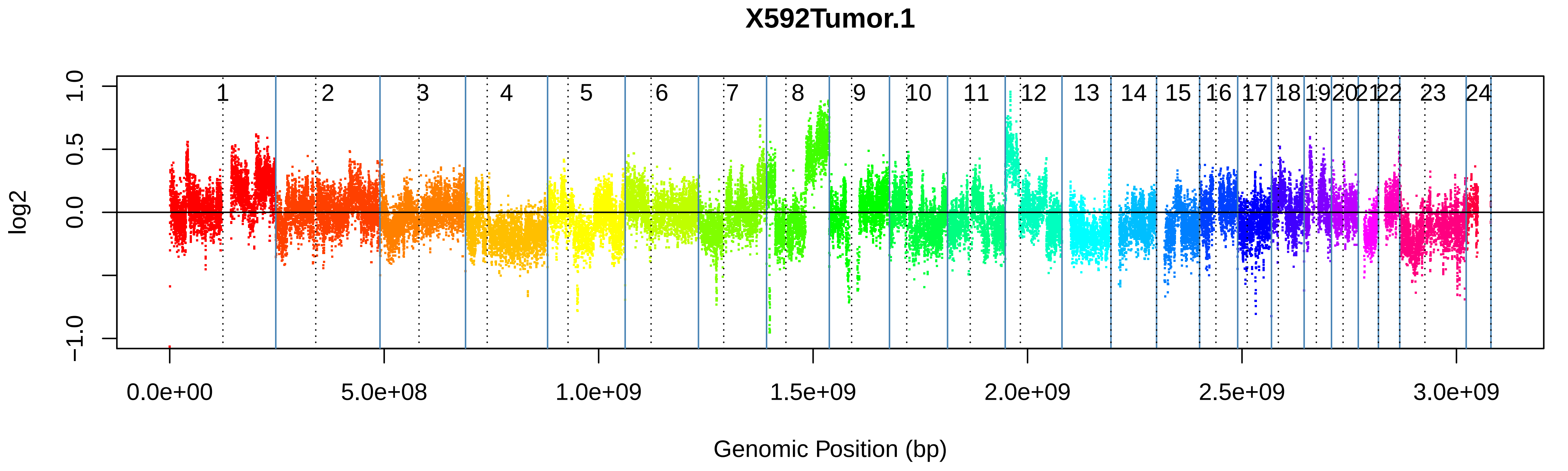
X592Tumor.1
log2
1.0
0.5
0.0
−1.0
1 2 3 4 5 6 7 8 9 10 11 12 13 14 15 16 17 18 19 20 21 22 23 24
0.0e+00
5.0e+08
1.0e+09
1.5e+09
2.0e+09
2.5e+09
3.0e+09
Genomic Position (bp)

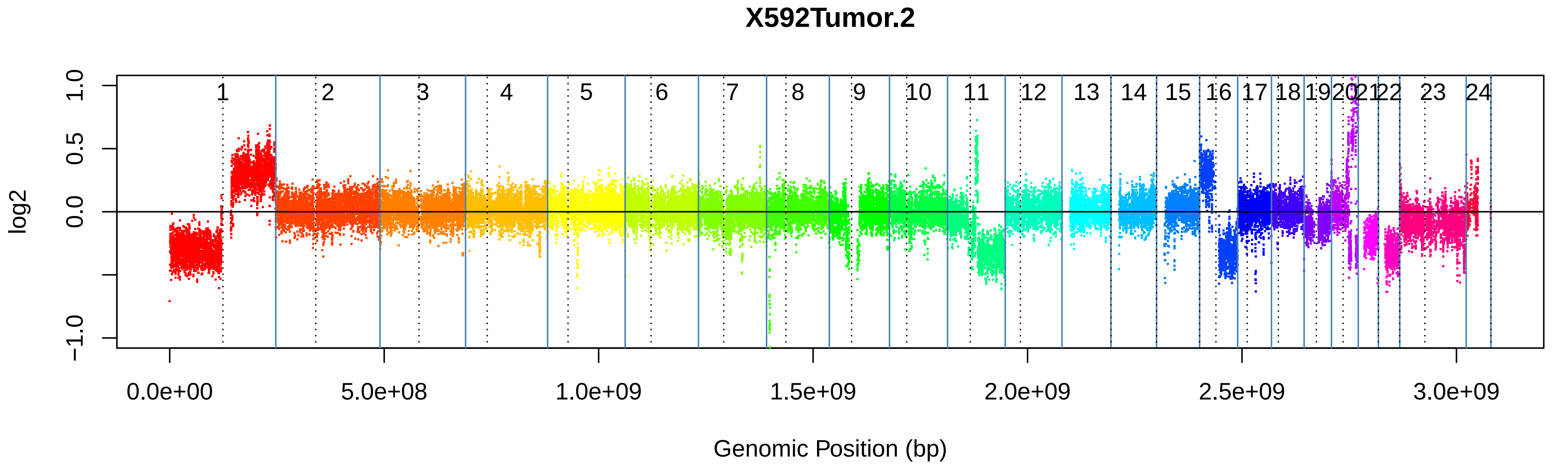
X592Tumor.2
log2
1.0
0.5
0.0
−1.0
1 2 3 4 5 6 7 8 9 10 11 12 13 14 15 16 17 18 19 20 21 22 23 24
0.0e+00
5.0e+08
1.0e+09
1.5e+09
2.0e+09
2.5e+09
3.0e+09
Genomic Position (bp)
