# Supplementary material for: Lack of Genomic Heterogeneity at High-Resolution aCGH between Primary Breast Cancers and Their Paired Lymph Node Metastases
Source: PLoS One. 2014 Aug 1;9(8):e103177. doi: 10.1371/journal.pone.0103177 (PMC4118860; doi:10.1371/journal.pone.0103177)
Supplement: File S4 — Tables S1-S3. Table S1, Patient characteristics of patients with 720 K aCGH data available. Table S2, Overview of all overlapping delta segments. Table S3, Patient characteristics of all patients. (DOCX) [file pone.0103177.s006.docx]

**Supplementary Table 1. Patient characteristics**

| **Variable** |  | **No. of patients with luminal tumours** | **No. of patients with TNBC** | **p-value** |
| --- | --- | --- | --- | --- |
|  |  |  |  |  |
| **Age** |  |  |  |  |
| < 40 years |  | 3 | 3 |  |
| 40 - 49 years |  | 7 | 4 | 0.51* |
| > 50 years |  | 0 | 3 |  |
|  |  |  |  |  |
| **Pathological tumour classification** |  |  |  |  |
| T1 |  | 1 | 2 |  |
| T2 |  | 7 | 6 | 1.00* |
| T3 |  | 2 | 2 |  |
|  |  |  |  |  |
| **No. of positive lymph nodes** |  |  |  |  |
| 4-9 |  | 9 | 6 | 0.30 |
| > 10 |  | 1 | 4 |  |
|  |  |  |  |  |
| **Histologic grade** |  |  |  |  |
| I |  | 2 | 0 |  |
| II |  | 5 | 2 | 0.04* |
| III |  | 3 | 7 |  |
| Not determined |  | 0 | 1 |  |
|  |  |  |  |  |
| **Estrogen receptor status** |  |  |  |  |
| Negative (<10%) |  | 0 | 10 | nr |
| Positive (>10%) |  | 10 | 0 |  |
|  |  |  |  |  |
| **Progesterone receptor status** |  |  |  |  |
| Negative (<10%) |  | 2 | 10 | nr |
| Positive (>10%) |  | 8 | 0 |  |
|  |  |  |  |  |
| **P53 status** |  |  |  |  |
| < 10% |  | 6 | 2 |  |
| 10 – 50% |  | 3 | 1 | 0.06* |
| > 50% |  | 1 | 5 |  |
| Unknown |  | 0 | 2 |  |
|  |  |  |  |  |
| p values: patients with unknown values were omitted. p values were calculated using the Fisher’s exact test, except for * Chi square test for trend. *Abbreviations:* no. number; nr, not relevant selection on these variables | | | | |

**Supplementary Table 2: Overview of all overlapping delta segments.**

| **Patient nr** | **chrom** | **chrom start** | **chrom end** | **num.mark** | **seg.mean** | **subtype** | **Tumour state** | **LN state** | **combined state** |
| --- | --- | --- | --- | --- | --- | --- | --- | --- | --- |
| 355 | 1 | 39679781 | 47438957 | 2019 | 0.2541 | TNBC | none | gain | none->gain |
| 881 | 1 | 45854854 | 46069799 | 49 | 0.3261 | TNBC | none | none | none->none |
| 355 | 1 | 47615749 | 53435570 | 1623 | 0.2267 | TNBC | none | gain | none->gain |
| 565 | 1 | 49669820 | 50287337 | 152 | -0.5004 | ER+ | none | loss | none->loss |
| 565 | 1 | 50583807 | 50722208 | 40 | -0.4561 | ER+ | none | loss | none->loss |
| 565 | 1 | 51123165 | 51458679 | 69 | -0.4916 | ER+ | none | loss | none->loss |
| 881 | 1 | 158175215 | 187127014 | 7617 | -0.2519 | TNBC | gain | gain | gain->gain |
| 396 | 1 | 160415206 | 160521574 | 44 | -0.3742 | TNBC | gain | gain | gain->gain |
| 322 | 1 | 171421653 | 172717046 | 281 | -0.3455 | TNBC | gain | gain | gain->gain |
| 122 | 1 | 172061131 | 172105207 | 11 | 0.2882 | ER+ | gain | gain | gain->gain |
| 881 | 2 | 19799 | 75357291 | 19716 | 0.2314 | TNBC | none | none | none->none |
| 841 | 2 | 9997778 | 10039866 | 10 | 0.246 | TNBC | gain | gain | gain->gain |
| 355 | 2 | 19519744 | 20741756 | 328 | 0.3051 | TNBC | loss | none | loss->none |
| 524 | 3 | 65164956 | 65194349 | 11 | 0.4146 | ER+ | loss | loss | loss->loss |
| 355 | 3 | 65164956 | 65188166 | 10 | -0.2444 | TNBC | loss | loss | loss->loss |
| 881 | 4 | 132802986 | 133122935 | 23 | -0.3888 | TNBC | gain | none | gain->none |
| 122 | 4 | 132805248 | 133077170 | 18 | -0.3762 | ER+ | gain | none | gain->none |
| 61 | 5 | 150120655 | 180814162 | 9355 | -0.2229 | TNBC | gain | none | gain->none |
| 355 | 5 | 177296269 | 180344711 | 718 | 0.2825 | TNBC | loss | none | loss->none |
| 881 | 5 | 180275126 | 180362377 | 11 | -0.3516 | TNBC | loss | loss | loss->loss |
| 322 | 5 | 180293675 | 180718374 | 66 | -0.4276 | TNBC | gain | gain | gain->gain |
| 355 | 5 | 180362377 | 180814162 | 62 | 0.2717 | TNBC | loss | none | loss->none |
| 881 | 7 | 35249507 | 57575557 | 6083 | 0.2795 | TNBC | loss | none | loss->none |
| 782 | 7 | 38281394 | 38326725 | 14 | 0.3444 | ER+ | none | gain | none->gain |
| 355 | 7 | 118942521 | 158816034 | 10700 | -0.2083 | TNBC | gain | none | gain->none |
| 44 | 7 | 158306644 | 158816034 | 117 | -0.2824 | ER+ | none | loss | none->loss |
| 881 | 8 | 7219480 | 137748739 | 32716 | 0.2968 | TNBC | gain | gain | gain->gain |
| 122 | 8 | 8442371 | 9047328 | 159 | 0.2402 | ER+ | gain | gain | gain->gain |
| 122 | 8 | 67605281 | 67856039 | 63 | 0.3198 | ER+ | gain | gain | gain->gain |
| 355 | 8 | 117303752 | 117333429 | 15 | 0.2793 | TNBC | gain | gain | gain->gain |
| 322 | 8 | 132681101 | 146264232 | 3519 | -0.2726 | TNBC | gain | none | gain->none |
| 881 | 8 | 137984221 | 146264232 | 1876 | 0.3458 | TNBC | gain | gain | gain->gain |
| 355 | 10 | 128680 | 2982309 | 837 | -0.2681 | TNBC | none | loss | none->loss |
| 425 | 10 | 128680 | 38553648 | 10018 | -0.2595 | TNBC | gain | none | gain->none |
| 355 | 10 | 84587705 | 89380669 | 1143 | -0.2498 | TNBC | none | none | none->none |
| 122 | 10 | 89038683 | 89248990 | 41 | -0.2747 | ER+ | none | none | none->none |
| 322 | 11 | 66612 | 58026741 | 12984 | -0.2275 | TNBC | none | loss | none->loss |
| 565 | 11 | 3217293 | 3326093 | 22 | -0.3249 | ER+ | gain | gain | gain->gain |
| 841 | 11 | 12649765 | 12671448 | 11 | 0.25 | TNBC | loss | none | loss->none |
| 841 | 11 | 57285610 | 57325807 | 12 | 0.278 | TNBC | none | gain | none->gain |
| 322 | 11 | 58027476 | 82380367 | 5835 | 0.3028 | TNBC | none | gain | none->gain |
| 104 | 11 | 60728061 | 60774450 | 12 | -0.2091 | ER+ | loss | loss | loss->loss |
| 841 | 11 | 64943448 | 65048463 | 24 | 0.2366 | TNBC | none | gain | none->gain |
| 355 | 11 | 76894678 | 134444816 | 15428 | -0.2282 | TNBC | gain | none | gain->none |
| 122 | 11 | 125795630 | 125853430 | 17 | 0.3797 | ER+ | loss | none | loss->none |
| 881 | 12 | 3853197 | 5978832 | 659 | -0.6829 | TNBC | gain | loss | gain->loss |
| 355 | 12 | 4399991 | 4431887 | 12 | -0.5666 | TNBC | gain | gain | gain->gain |
| 355 | 12 | 4446083 | 8200963 | 942 | -0.2739 | TNBC | gain | gain | gain->gain |
| 881 | 12 | 5990965 | 8200963 | 459 | -0.3743 | TNBC | none | loss | none->loss |
| 881 | 12 | 8495584 | 10031215 | 251 | -0.4175 | TNBC | none | loss | none->loss |
| 355 | 12 | 8495584 | 14822550 | 1361 | -0.2617 | TNBC | gain | gain | gain->gain |
| 881 | 12 | 13172917 | 13359121 | 64 | -0.3948 | TNBC | loss | loss | loss->loss |
| 122 | 14 | 18144040 | 19309732 | 55 | -0.2613 | ER+ | none | none | none->none |
| 425 | 14 | 18144040 | 19270761 | 51 | 0.2501 | TNBC | loss | loss | loss->loss |
| 122 | 14 | 66868322 | 68623996 | 533 | 0.2931 | ER+ | loss | none | loss->none |
| 788 | 14 | 67621362 | 68162212 | 170 | -0.4298 | TNBC | loss | loss | loss->loss |
| 788 | 14 | 89712531 | 106342076 | 4523 | -0.2268 | TNBC | loss | loss | loss->loss |
| 104 | 14 | 105920708 | 106015985 | 15 | -0.2215 | ER+ | loss | loss | loss->loss |
| 245 | 15 | 18420959 | 100296299 | 20478 | -0.2134 | ER+ | gain | none | gain->none |
| 104 | 15 | 85629520 | 85679279 | 12 | -0.283 | ER+ | loss | loss | loss->loss |
| 122 | 15 | 91141932 | 91271477 | 27 | 0.3847 | ER+ | gain | gain | gain->gain |
| 322 | 15 | 96644380 | 97874183 | 339 | -0.3225 | TNBC | gain | none | gain->none |
| 322 | 15 | 99302092 | 100296299 | 253 | -0.3093 | TNBC | gain | none | gain->none |
| 322 | 17 | 54833495 | 55604525 | 161 | -0.5894 | TNBC | gain | gain | gain->gain |
| 841 | 17 | 55191837 | 55290343 | 12 | 0.2712 | TNBC | none | none | none->none |
| 355 | 17 | 55218888 | 55333850 | 16 | 0.2932 | TNBC | gain | gain | gain->gain |
| 795 | 20 | 15928 | 32627779 | 7504 | 0.2222 | TNBC | none | none | none->none |
| 322 | 20 | 13176553 | 13594409 | 109 | -0.5331 | TNBC | gain | gain | gain->gain |
| 322 | 20 | 15945815 | 16107094 | 46 | -0.5627 | TNBC | gain | gain | gain->gain |
| 524 | 22 | 14434579 | 49568253 | 8898 | 0.2147 | ER+ | loss | loss | loss->loss |
| 881 | 22 | 14434579 | 15535092 | 37 | -0.2014 | TNBC | gain | none | gain->none |
| 565 | 22 | 25766514 | 26701689 | 344 | -0.446 | ER+ | none | loss | none->loss |
| 565 | 22 | 27009001 | 27058008 | 14 | -0.5082 | ER+ | none | loss | none->loss |
| 565 | 22 | 27995406 | 28319989 | 99 | -0.4624 | ER+ | none | loss | none->loss |
| 565 | 22 | 28551441 | 28603176 | 24 | -0.5078 | ER+ | none | loss | none->loss |
| 565 | 22 | 28651960 | 28740011 | 22 | -0.4262 | ER+ | none | loss | none->loss |
| 565 | 22 | 29117499 | 29306821 | 48 | -0.4308 | ER+ | none | loss | none->loss |
| 565 | 22 | 29853956 | 35510186 | 1809 | -0.4701 | ER+ | none | loss | none->loss |
| 565 | 22 | 35723318 | 35809021 | 23 | -0.4355 | ER+ | none | loss | none->loss |
| 565 | 22 | 35868455 | 35972684 | 27 | -0.4073 | ER+ | none | loss | none->loss |
| 565 | 22 | 36419455 | 37109004 | 162 | -0.4493 | ER+ | none | loss | none->loss |
| 782 | 22 | 36924170 | 37039453 | 30 | 0.254 | ER+ | none | none | none->none |
| 565 | 22 | 37128927 | 41456123 | 1144 | -0.4598 | ER+ | none | loss | none->loss |
| 565 | 22 | 41573513 | 41897062 | 76 | -0.4411 | ER+ | none | loss | none->loss |
| 565 | 22 | 42023735 | 42071537 | 16 | -0.4206 | ER+ | none | loss | none->loss |
| 782 | 22 | 42988464 | 43251496 | 55 | 0.3099 | ER+ | none | none | none->none |
| 795 | 23 | 146809526 | 154895334 | 2316 | 0.3669 | TNBC | loss | none | loss->none |
| 396 | 23 | 153075386 | 153153323 | 24 | -0.3291 | TNBC | gain | none | gain->none |
| 44 | 24 | 57458252 | 57758621 | 86 | 0.2915 | ER+ | none | gain | none->gain |
| 795 | 24 | 57490740 | 57758621 | 81 | 0.3164 | TNBC | loss | none | loss->none |
| *Abbreviations:* nr, number; chrom, chromosome; seg, segment. Samples below dashed lines also share a regions with tumours above this line.  Tumor state and LN state are defined as gain for a log2 segmentation mean > 0.2 and < -0.2 respectively and are called on the segmentation of the individual tumor and LN samples. | | | | | | | | | |

**Supplementary Table 3. Patient characteristics of all patients**

| **Variable** |  | **No. of patients with ER+ tumors** | **No. of patients with TN tumors** | **p-value** |
| --- | --- | --- | --- | --- |
|  |  |  |  |  |
| **Age** |  |  |  |  |
| < 40 years |  | 9 | 3 |  |
| 40 - 49 years |  | 19 | 6 | 0.12* |
| > 50 years |  | 4 | 6 |  |
|  |  |  |  |  |
| **Pathological tumour classification** |  |  |  |  |
| T1 |  | 3 | 3 |  |
| T2 |  | 24 | 9 | 1.00* |
| T3 |  | 4 | 3 |  |
| Unknown |  | 1 |  |  |
|  |  |  |  |  |
| **No. of positive lymph nodes** |  |  |  |  |
| 4-9 |  | 22 | 8 | 0.34 |
| > 10 |  | 10 | 7 |  |
|  |  |  |  |  |
| **Histologic grade** |  |  |  |  |
| I |  | 9 | 0 |  |
| II |  | 16 | 4 | < 0.001* |
| III |  | 7 | 10 |  |
| Not determined |  | 0 | 1 |  |
|  |  |  |  |  |
| **Estrogen receptor status** |  |  |  |  |
| Negative (<10%) |  | 0 | 15 | nr |
| Positive (>10%) |  | 32 | 0 |  |
|  |  |  |  |  |
| **Progesterone receptor status** |  |  |  |  |
| Negative (<10%) |  | 6 | 15 | nr |
| Positive (>10%) |  | 26 | 0 |  |
|  |  |  |  |  |
| **P53 status** |  |  |  |  |
| < 10% |  | 22 | 5 |  |
| 10 – 50% |  | 7 | 1 | 0.02* |
| > 50% |  | 3 | 7 |  |
| Unknown |  | 0 | 2 |  |
|  |  |  |  |  |
| p values: patients with unknown values were omitted. p values were calculated using the Fisher’s exact test, except for * Chi square test for trend. *Abbreviations:* no. number; nr, not relevant selection on these variables | | | | |
